# The Role of Viral Introductions in Sustaining Community-Based HIV Epidemics in Rural Uganda: Evidence from Spatial Clustering, Phylogenetics, and Egocentric Transmission Models

**DOI:** 10.1371/journal.pmed.1001610

**Published:** 2014-03-04

**Authors:** Mary K. Grabowski, Justin Lessler, Andrew D. Redd, Joseph Kagaayi, Oliver Laeyendecker, Anthony Ndyanabo, Martha I. Nelson, Derek A. T. Cummings, John Baptiste Bwanika, Amy C. Mueller, Steven J. Reynolds, Supriya Munshaw, Stuart C. Ray, Tom Lutalo, Jordyn Manucci, Aaron A. R. Tobian, Larry W. Chang, Chris Beyrer, Jacky M. Jennings, Fred Nalugoda, David Serwadda, Maria J. Wawer, Thomas C. Quinn, Ronald H. Gray, the Rakai Health Sciences Program

**Affiliations:** 1 Department of Epidemiology, Bloomberg School of Public Health, Johns Hopkins University, Baltimore, Maryland, United States of America; 2 Laboratory of Immunoregulation, Division of Intramural Research, National Institute of Allergy and Infectious Diseases, National Institutes of Health, Bethesda, Maryland, United States of America; 3 Rakai Health Sciences Program, Kalisizo, Uganda; 4 School of Medicine, Johns Hopkins University, Baltimore, Maryland, United States of America; 5 Division of International Epidemiology and Population Studies, Fogarty International Center, National Institutes of Health, Bethesda, Maryland, United States of America; 6 School of Public Health, College of Medicine, Makerere University, Kampala, Uganda; Imperial College London, United Kingdom

## Abstract

**Background:**

It is often assumed that local sexual networks play a dominant role in HIV spread in sub-Saharan Africa. The aim of this study was to determine the extent to which continued HIV transmission in rural communities—home to two-thirds of the African population—is driven by intra-community sexual networks versus viral introductions from outside of communities.

**Methods and Findings:**

We analyzed the spatial dynamics of HIV transmission in rural Rakai District, Uganda, using data from a cohort of 14,594 individuals within 46 communities. We applied spatial clustering statistics, viral phylogenetics, and probabilistic transmission models to quantify the relative contribution of viral introductions into communities versus community- and household-based transmission to HIV incidence. Individuals living in households with HIV-incident (*n* = 189) or HIV-prevalent (*n* = 1,597) persons were 3.2 (95% CI: 2.7–3.7) times more likely to be HIV infected themselves compared to the population in general, but spatial clustering outside of households was relatively weak and was confined to distances <500 m. Phylogenetic analyses of *gag* and *env* genes suggest that chains of transmission frequently cross community boundaries. A total of 95 phylogenetic clusters were identified, of which 44% (42/95) were two individuals sharing a household. Among the remaining clusters, 72% (38/53) crossed community boundaries. Using the locations of self-reported sexual partners, we estimate that 39% (95% CI: 34%–42%) of new viral transmissions occur within stable household partnerships, and that among those infected by extra-household sexual partners, 62% (95% CI: 55%–70%) are infected by sexual partners from outside their community. These results rely on the representativeness of the sample and the quality of self-reported partnership data and may not reflect HIV transmission patterns outside of Rakai.

**Conclusions:**

Our findings suggest that HIV introductions into communities are common and account for a significant proportion of new HIV infections acquired outside of households in rural Uganda, though the extent to which this is true elsewhere in Africa remains unknown. Our results also suggest that HIV prevention efforts should be implemented at spatial scales broader than the community and should target key populations likely responsible for introductions into communities.

*Please see later in the article for the Editors' Summary*

## Abstract 2

Using different approaches to investigate HIV transmission patterns, Justin Lessler and colleagues find that extra-community HIV introductions are frequent and likely play a role in sustaining the epidemic in the Rakai community.

*Please see later in the article for the Editors' Summary*

## Editors' Summary

**Background:**

About 35 million people (25 million of whom live in sub-Saharan Africa) are currently infected with HIV, the virus that causes AIDS, and about 2.3 million people become newly infected every year. HIV destroys immune system cells, leaving infected individuals susceptible to other infections. HIV infection can be controlled by taking antiretroviral drugs (antiretroviral therapy, or ART) daily throughout life. Although originally available only to people living in wealthy countries, recent political efforts mean that 9.7 million people in low- and middle-income countries now have access to ART. However, ART does not cure HIV infection, so prevention of viral transmission remains extremely important. Because HIV is usually transmitted through unprotected sex with an infected partner, individuals can reduce their risk of infection by abstaining from sex, by having one or a few partners, and by using condoms. Male circumcision also reduces HIV transmission. In addition to reducing illness and death among HIV-positive people, ART also reduces HIV transmission.

**Why Was This Study Done?:**

Effective HIV control requires an understanding of how HIV spreads through sexual networks. These networks include sexual partnerships between individuals in households, between community members in different households, and between individuals from different communities. Local sexual networks (household and intra-community sexual partnerships) are sometimes assumed to be the dominant driving force in HIV spread in sub-Saharan Africa, but are viral introductions from sexual partnerships with individuals outside the community also important? This question needs answering because the effectiveness of interventions such as ART as prevention partly depends on how many new infections in an intervention area are attributable to infection from partners residing in that area and how many are attributable to infection from partners living elsewhere. Here, the researchers use three analytical methods—spatial clustering statistics, viral phylogenetics, and egocentric transmission modeling—to ask whether HIV transmission in rural Uganda is driven predominantly by intra-community sexual networks. Spatial clustering analysis uses the geographical coordinates of households to measure the tendency of HIV-infected people to cluster spatially at scales consistent with community transmission. Viral phylogenetic analysis examines the genetic relatedness of viruses; if transmission is through local networks, viruses in newly infected individuals should more closely resemble viruses in other community members than those in people outside the community. Egocentric transmission modelling uses information on the locations of recent sexual partners to estimate the proportions of new transmissions from household, intra-community, and extra-community partners.

**What Did the Researchers Do and Find?:**

The researchers applied their three analytical methods to data collected from 14,594 individuals living in 46 communities (governmental administrative units) in Rakai District, Uganda. Spatial clustering analysis indicated that individuals who lived in households with individuals with incident HIV (newly diagnosed) or prevalent HIV (previously diagnosed) were 3.2 times more likely than the general population to be HIV-positive themselves. Spatial clustering outside households was relatively weak, however, and was confined to distances of less than half a kilometer. Viral phylogenetic analysis indicated that 44% of phylogenetic clusters (viruses with related genetic sequences found in more than one individual) were within households, but that 40% of clusters crossed community borders. Finally, analysis of the locations of self-reported sexual partners indicated that 39% of new viral transmissions occurred within stable household partnerships, but that among people newly infected by extra-household partners, nearly two-thirds were infected by partners from outside their community.

**What Do These Findings Mean?:**

The results of all three analyses suggest that HIV introductions into communities are frequent and are likely to play an important role in sustaining HIV transmission in the Rakai District. Specifically, within this rural HIV-endemic region (a region where HIV infection is always present), viral introductions combined with intra-household transmission account for the majority of new infections, although community-based sexual networks also play a critical role in HIV transmission. These findings may not be generalizable to the broader Ugandan population or to other regions of Africa, and their accuracy is likely to be limited by the use of self-reported sexual partner data. Nevertheless, these findings indicate that the dynamics of HIV transmission in rural Uganda (and probably elsewhere) are complex. Consequently, to halt the spread of HIV, prevention efforts will need to be implemented at spatial scales broader than individual communities, and key populations that are likely to introduce HIV into communities will need to be targeted.

**Additional Information:**

Please access these websites via the online version of this summary at http://dx.doi.org/10.1371/journal.pmed.1001610.

- Information is available from the US National Institute of Allergy and Infectious Diseases on HIV infection and AIDS
- NAM/aidsmap provides basic information about HIV/AIDS, and summaries of recent research findings on HIV care and treatment
- Information is available from Avert, an international AIDS charity, on many aspects of HIV/AIDS, including information on HIV and AIDS in Uganda and on HIV prevention strategies (in English and Spanish)
- The UNAIDS Report on the Global AIDS Epidemic 2013 provides up-to-date information about the AIDS epidemic and efforts to halt it
- The Center for AIDS Prevention Studies (University of California, San Francisco) has a fact sheet about sexual networks and HIV prevention
- Wikipedia provides information on spatial clustering analysis (note that Wikipedia is a free online encyclopedia that anyone can edit; available in several languages)
- A *PLOS Computational Biology* Topic Page (a review article that is a published copy of record of a dynamic version of the article as found in Wikipedia) about viral phylodynamics is available
- Personal stories about living with HIV/AIDS are available through Avert, NAM/aidsmap, and Healthtalkonline

## Introduction

Effective prevention and control of the human immunodeficiency virus (HIV) builds upon an understanding of the dynamics that sustain viral transmission within sexual networks [1],[2]. These networks are comprised of sexual partnerships between individuals within households, between community members not sharing a household, and between individuals in different communities. While sufficiently large intra-community sexual networks can potentially maintain local HIV epidemics, virus introduced from sources external to the community may also sustain incidence [3],[4]. The effectiveness of interventions designed to prevent HIV transmission within a given community or any other geographic unit depends in part upon the attributable fraction of new cases infected through partners residing within the targeted area and those infected from partners residing outside of that area [4]–[7]. These proportions are particularly relevant to population-based antiretroviral therapy (ART) strategies for HIV prevention that aim to benefit individuals who do not themselves receive the treatment by reducing their risk of infection.

In 2011, ART was established as a highly effective tool for HIV prevention in the landmark HPTN 052 clinical trial [8], which showed that ART almost universally prevents HIV transmission within HIV-discordant couples [8],[9]. The concept of ART for HIV prevention (“treatment as prevention”) is now widely accepted, and in 2012, it was adopted by the US President's Emergency Program for AIDS Relief as a key strategy for population-based HIV control [10]. Despite the widely heralded success of HPTN 052, it is unknown whether ART can be scaled to levels necessary to interrupt community-level HIV transmission. Uncertainty remains, in part, because the treated population in HPTN 052 represented a unique subset of the total HIV-infected population: participants were in the chronic stages of HIV infection, receiving care for their disease, and in a stable sexual partnership [8]. Transmission in the broader population occurs along a complex sexual network in which virus is transmitted by infected individuals in early and chronic stages of HIV infection and between individuals who may or may not be in stable sexual partnerships. These complexities have motivated large community-randomized controlled trials (CRCTs) of ART for HIV prevention in African populations, including the HPTN 071 study in Zambia and South Africa [11] and the Mochudi Prevention Project in Botswana [12]. By virtue of their community-randomized design, these CRCTs presume that the preponderance of viral transmissions occur between partners residing within the same communities of randomization [13]; however, it is unknown what fraction of HIV transmissions in Africa occur within communities versus across community boundaries.

The empirical study of HIV transmission outside of stable couples is challenging, but new approaches to epidemiological inference and evolutionary biology provide unprecedented opportunities to understand the spatial scale of HIV transmission networks. Here we test the hypothesis that extra-household HIV transmission is predominately sustained through intra-community sexual networks using population-based cohort data from 14,594 individuals, including 189 individuals with incident HIV residing within 46 communities in the Rakai District, Uganda. Rakai, bordered by Tanzania to the south and Lake Victoria to the east, is rural and represents one of the earliest epicenters of the HIV/AIDS epidemic in east Africa [14]. Presently, HIV transmission in Rakai is endemic, with circulation of HIV-1 subtypes A, D, and C, and multiple recombinant viruses [15].

Our study consists of three primary analyses, in all of which the primary geographic unit of interest was the community. In the first analysis we used the geographic coordinates of participant households and measured the tendency of HIV-seropositive persons to spatially cluster within and outside of communities. If local transmission dynamics dominate, we expect infected persons to spatially cluster at geographic distances consistent with intra-community transmission. In the second analysis we examined the genetic relatedness of infecting viruses within communities. If transmission is sustained through local sexual networks, viruses within newly infected persons should be more similar to viruses of other HIV-infected persons within the community than to those of individuals outside the community. Finally, we used egocentric network information on the geographic locations of recent sexual partners to estimate the proportions of new transmissions occurring between household, community, and extra-community partners. In this third analysis we also estimated the proportion of household transmissions occurring within 1 y of an index household infection. Each of these three independent, yet complementary, analyses has its own strengths and weaknesses, and together they are a powerful set of inferential tools for understanding the spatial scale and structure of HIV transmission networks.

## Methods

### Ethics Statement

The study was independently reviewed and approved by Ugandan (Ugandan Virus Research Institute Security and Ethics Committee; Protocol GC/127/13/01/16) and US (Western Institutional Review Board; Protocol 200313317) institutional review boards. All study participants provided written informed consent at baseline and follow-up visits using institutional review board–approved forms.

### Study Population and Setting

**Figure 1:**
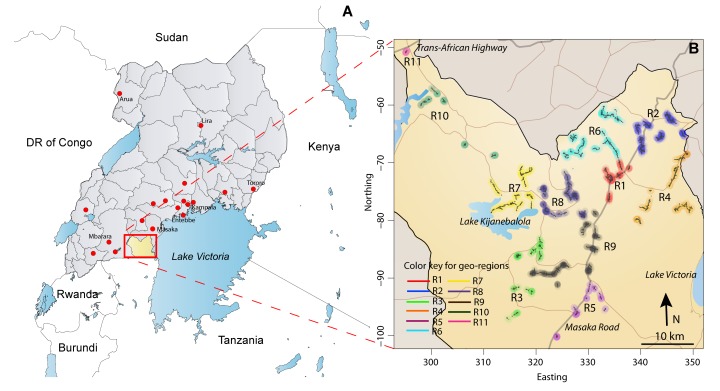
Rakai District, Uganda. (A) Rakai (∼2,200 km^2^), a rural district in southwest Uganda, with population ∼450,000 (∼700 communities). RCCS R13 study participants (*n* = 1,085) reported 1,169 sexual partners with primary residence outside the Rakai District, but within Uganda (where disclosed, residential locations of sexual partners are indicated with red dots on the map). Only three sexual partners were reported to be living outside Uganda (two in Tanzania and one in the United Kingdom, not shown). (B) The Rakai district at a higher resolution, with the 11 geographic regions surveyed in RCCS R13 indicated in color. There are two primary highways (Masaka Road to Tanzania and the Trans-African National Highway to Rwanda and the Democratic Republic of the Congo [DR of Congo]) and numerous secondary roads that extend throughout the district.

The Rakai Community Cohort Study (RCCS) is a well-characterized population-based HIV surveillance cohort in the Rakai District, Uganda (Figure 1A). Methods for the RCCS have been described in detail elsewhere [6]. Briefly, the RCCS enrolls all consenting persons aged 15–49 y residing in 50 village communities. The RCCS defines households as a group of persons who sleep under one roof and eat out of a common pot, and a community as an administrative unit whose boundaries are determined by the Ugandan government (Local Council 1 and Local Council 2 units, the two smallest political units in Uganda). Eleven larger community groupings (2–8 communities each), referred to as geographic regions, were previously designated by the RCCS based upon geographic proximity and the frequency of cross-community contact (Figure 1B) [6].

Study participants are administered a detailed questionnaire at visits occurring every 12–18 mo and provide a serological sample at each visit. HIV serostatus is assessed by two enzyme immunoassays (Vironostika HIV-1, BioMerieux, and Recombigen, Cambridge Biotech), with Western blot confirmation of discordant enzyme immunoassays and for all HIV seroconverters (HIV-1 WB, BioMerieux-Vitek). RCCS participation rates are ∼90% of persons present at time of survey, and follow-up rates between successive visits are ∼75%.

In this study, we used data from RCCS survey round 13 (RCCS R13) for all data analyses (spatial clustering, viral phylogenetics, and egocentric transmission models). RCCS R13 was conducted between June 17, 2008, and December 7, 2009, within 46 of the 50 RCCS communities. It included surveys of 14,594 participants residing in 8,899 households, the collection of household GPS coordinates (8,156/8,899, or 91.6% of study households; resolution ∼3–5 m), and viral sequencing for ART-naïve HIV-seropositive individuals. Participants who were HIV seropositive upon entry into RCCS R13 were defined as HIV seroprevalent in all analyses. The average maximum distance between any two households within a community (i.e., the community size) was ∼3 km (Figure S1). Though our three primary analyses use data drawn from the same study population (RCCS R13), each analysis was conducted independently of the others.

### Spatial Clustering Analysis

Using the geographic coordinates of participant households in RCCS R13 the spatial relatedness between HIV-seropositive individuals was characterized by τ(*d*_1_,*d*_2_), defined as the relative probability that a participant A residing within a distance range, *d*_1_ to *d*_2_, from an HIV-seropositive participant B was also HIV seropositive versus the probability that any RCCS participant was HIV seropositive, regardless of spatial location [16]. It is estimated as:
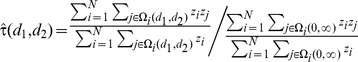
(1)where Ω*_i_*(*d*_1_,*d*_2_) is the set of points in spatial range (*d*_1_,*d*_2_) of point *i*, and *Z_i_* indicates seropositivity. We also measured the spatial clustering of seroincident cases with other seroincident cases, and of seroincident cases with HIV-seroprevalent persons on and off ART. Values of τ(*d*_1_,*d*_2_) were calculated at 0 m (household) and for 250-m wide windows centered from 125 m to 30 km in 50-m increments. Where spatial clustering exists, τ(*d*_1_,*d*_2_) will be greater than 1. The significance of clustering was assessed by bootstrapping (1,000 iterations), where pairs of individuals were sampled with replacement. Instead of resampling individuals, samples were drawn from all possible pairs of individuals in the study to ensure no comparisons occurred between an individual and him/herself in bootstrapped samples.

### Viral Extractions and HIV-1 Subtype Assignment

Viral RNA extractions were performed on sera of all ART-naïve HIV-seropositive participants in RCCS R13 (*n* = 1,434) using the QiAmp Viral Mini Kit (Qiagen). Extracted RNA was amplified by reverse transcription PCR and an additional nested PCR in two separate assays for partial *gag* (HXB2 nucleotides 1249 to 1704) and *env* (HBX2 nucleotides 7858 to 8260) sequences, as previously described [15],[17]. RNA extractions and PCR assays were conducted in separate designated laboratory spaces for quality control. HIV amplicons were sequenced using direct Sanger methods on the Applied Biosystems 373xl DNA Analyzer. Results were examined immediately for contamination and batch effects. We also repeated testing for a subset of specimens (extraction through sequencing). Sequential samples from the same individual always clustered together when compared using phylogenetic methods (Figure S2).

HIV-1 subtype assignments were made using the US National Center for Biotechnology Information genotyping database and then confirmed phylogenetically with reference sequences from the Los Alamos National Laboratory HIV Sequence Database (HIVDB). Sequences were aligned with MUSCLE v3.7 and manually edited in Bioedit v7.1.3 [18]. Ambiguous regions in sequence alignments were removed using GBLOCKS v0.91b [19]. Final alignments were ∼564 bp in the *gag* gene and ∼467 bp in the *env* gene. Sequences were scanned with all available methods in the Recombination Detection Program v3.44 [20]. Within-gene recombination events identified in one or more analyses were verified using jumping hidden Markov models [21]. Intra-gene recombinant sequences were excluded from additional phylogenetic analyses (*gag*, *n* = 17; *env*, *n* = 8).

### Phylogenetic Analysis

Maximum likelihood (ML) methods under an HKY-85 model of nucleotide substitution were used to estimate genetic pairwise distances and reconstruct phylogenetic trees for *gag* and *env* genes and for HIV-1 A, D, and C subtypes separately (six datasets in total). African reference sequences (one per individual reference ID) were selected from the Los Alamos National Laboratory HIV Sequence Database for analyses. Using HKY-85 genetic pairwise distance, the three Los Alamos National Laboratory HIV Sequence Database reference sequences most similar to each participant's sequence were identified, and the unique subset of these sequences was defined as the reference set for RCCS R13 (Table S1). The reference set included viral sequences from all major geographic regions in sub-Saharan Africa.

ML phylogenetic trees were reconstructed under two models of nucleotide substitution, the HKY-85 model and the general time reversible model with gamma distributed rate heterogeneity and a proportion of invariable sites (GTR+I+G) [22],[23]. In the GTR+I+G model all possible nucleotide substitution rates are estimated, whereas in the HKY-85 model only transition and transversion rates are estimated (six versus two substitution rate parameters). We defined a cluster of related HIV cases as two or more participants whose sequences were contained within a monophyletic group in ML trees in either one or both gene regions (*gag* or *env*) at a bootstrap threshold of 90% or greater (1,000 replications). Clusters also met intra-cluster median genetic distance thresholds, where thresholds were defined using RCCS genetic data from epidemiologically linked HIV-incident couples (i.e., where at least one of the partners was an incident case). Specifically, genetic distance thresholds for each gene region were defined as the 95% quantile of the distribution of ML branch length distances between epidemiologically linked sexual partners (i.e., known couples) where at least one of the partners was an incident case and the partner sequences were contained within a monophyletic cluster with moderately high clade support (≥70%; Figure S3). Distance thresholds estimated for *gag* and *env* genes were 1.3% and 2.6%, respectively.

ML clusters were confirmed using Bayesian phylogenetics, where confirmation was established if the same sequences clustered together in the Bayesian tree with posterior probability equal to one. The ML tree topologies obtained using the more parameter-rich GTR+I+G model were similar to those obtained under the HKY-85 model, and so Bayesian confirmation of clusters was conducted using the HKY-85 model only. Bayesian analyses were conducted using MrBayes v3.2 [24], where trees were obtained through separate unconstrained phylogenetic analyses (i.e., no molecular clock) and each codon position was allowed to have its own site-specific rate. Four independent runs were performed for 3×10^8^ generations, and a burn-in of 25% was used for final analyses. Effective sample sizes for all parameters exceeded 200.

We assessed the sensitivity of our cluster definition using alternate cluster definitions in the ML analysis: 70%, 80%, 90%, and 99% bootstrap thresholds with and without genetic distance thresholds for HKY-85 and GTR+I+G models of substitution. We present the ML radial and square phylogenetic trees estimated under the HKY-85 model as figures in this article and in the Supporting Information. Community and household labels used in the square trees were blinded (i.e., true RCCS identification numbers were not used), and the exact community locations were not labeled on geographic maps to ensure the privacy of our study participants. The ML phylogenetic trees constructed under the GTR+I+G model and the Bayesian phylogenetic trees are available from the authors upon request.

### Egocentric Transmission Model

Study participants in RCCS R13 were asked about their most recent sexual partners (up to four partners, restricted to last 12 mo). Stable partnerships were defined as either marriages or long-term consensual unions. All other partner types (boyfriend/girlfriend and casual) were defined as non-stable. Participants were asked whether each sexual partner's primary residence was within the same household, within the same community, or outside of that individual's community. As per protocol, RCCS participant identifiers could be matched with a named partner only for stable (usually household) partners. If the stable partner was also an RCCS participant, we considered those partners to be epidemiologically linked. In instances where the epidemiologically linked partner did not participate in RCCS R13 but did so in a prior RCCS survey and he/she was HIV seropositive at his/her last study visit, we considered that partner HIV seroprevalent. When discrepancies between the self-reported geographic locations of household partners and GPS data obtained through RCCS were identified (∼2%, *n* = 256 self-reported partners), data were independently reviewed and adjudicated by study investigators (M. K. G., A. D. R.).

We considered a household HIV-seropositive partner to be on ART if that person was on ART for ≥50% of the inter-survey interval in which their initially uninfected partner was at risk for HIV. The RCCS has identified no HIV seroconversions within serodiscordant couples where the HIV-infected partner is on ART since ART was introduced in Rakai in 2004 [25]; therefore, we assumed that HIV-seropositive household partners on ART posed no risk to their uninfected partners in this analysis.

HIV sequence data for self-reported sexual partners was obtained only if those partners could be identified as being another RCCS participant, and this was possible only for stable partners. For phylogenetic methods to exclude any self-reported partner as a source of infection, sequences from all partners and the ability to detect co-infection are needed. As neither was available in this study, the egocentric transmission model and phylogenetics were conducted as independent, though complementary, analyses.

We used egocentric sexual partner data from HIV-seronegative and -incident participants (excluding those HIV-seronegative participants who entered into the study for the first time in RCCS R13 or who had missed more than two previous study visits) to model the probability of HIV infection from self-reported partners and unreported partners/sources as follows:
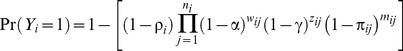
(2)where *Y_i_* is equal to 1 if participant *i* is an incident case; *n_i_* is the number of partners of case *i*; *w_ij_*, *z_ij_*, and *m_ij_* are indicators of whether partner *j* of case *i* is ART-naïve seroprevalent, incident, or missing HIV status, respectively; α and γ are the probabilities of infection from ART-naïve seroprevalent and incident partners, respectively, between study rounds; π*_ij_* is the probability of case *i* being infected by a partner *j* with missing status given their respective locations; and ρ*_i_* is the probability of *i* being infected from an unnamed partner/source.

The probability of infection from a self-reported partner of unknown HIV status was modeled as follows:
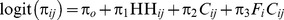
(3)where logit(π*_ij_*) is the log odds that *i* was infected by partner *j*, HH*_ij_* is an indicator of whether participant *i* shares a household with partner *j*, *C_ij_* is an indicator of whether the partner is outside the community, and *F_i_* is an indicator of whether partner *i* is female.

Parameters were estimated using Markov chain Monte Carlo methods. The numbers of infections attributable to specific partnership types were estimated by sampling parameters from the posterior distribution and then simulating sources of infections for each parameter set (250,000 iterations). In households where both partners had incident infection we initially randomly assigned one partner as having been infected first (i.e., without an identifiable incident partner) and the other partner as having been infected second (i.e., with an identifiable incident partner). Assignments were updated in each Markov chain Monte Carlo iteration and accepted or rejected using the standard Metropolis-Hastings criteria. For each incident infection, the probability of infection by each type of partner was calculated based upon the current parameter set and then normalized so that they summed to one (i.e., calculated conditional on that individual having been infected). Which partner (or unknown source) infected each individual was then randomly selected based upon these probabilities.

The sensitivity of the parameter estimates from our transmission model to unreported partnerships and misreported community status of partners was assessed by running 100 simulations where 10% of the reported partnerships in the original data were unreported and 100 simulations where the community status of 10% of extra-household partners was misreported (i.e., intra-community was changed to extra-community or vice versa).

### Accession Numbers

The 1,099 RCCS R13 sequences analyzed in this manuscript have been deposited in GenBank (http://www.ncbi.nlm.nih.gov/Genbank) under the accession numbers KJ373683–KJ374708 (*env*) and KJ372761–KJ373675 (*gag*).

## Results

There were 14,594 individuals who participated in RCCS R13 (2008–2009; Table 1), of whom 3,219 enrolled for the first time (7.8% were HIV seroprevalent at study entry, *n* = 252/3,219). More than 60% of the surveyed population was married (60.2%, *n* = 8,790/14,594), and slightly more than half of study participants were female (56.1%, *n* = 8,188/14,594). Study participants who were not in marital relationships included those who had never been married (27.3%, *n* = 3,982/14,594) or were previously but not currently married (12.3%, *n* = 1,795/14,594). Considering only married men, 15.3% (*n* = 560/3,664) were in polygamous unions.

**Table 1:** Summary statistics for the 46 Rakai communities (within 11 geographic regions) surveyed in RCCS R13.

Region	Community	Participants *N*	Female *n* (Percent)	Married^a^ *n* (Percent)	Households *n*	HIV Seropositive *n* (Percent)	HIV Seroincident *n*	Person-Years *n*	Incidence per 100 Person-Years^b^ (95% CI)
**Overall**		14,594	8,188 (56.1)	8,790 (60.2)	8,899	1,786 (12.2)	189	16,159.7	1.2 (1.0–1.3)
**1**	1	198	128 (64.6)	109 (55.1)	143	46 (23.2)	6	163.8	3.7 (1.3–8.0)
	2	543	335 (61.7)	316 (58.2)	347	85 (15.7)	8	518.0	1.5 (0.7–3.0)
	3	154	85 (55.2)	67 (43.5)	74	18 (11.7)	3	158.4	1.9 (0.4–5.5)
	4	388	227 (58.5)	224 (57.7)	249	62 (16.0)	6	382.3	1.6 (0.6–3.4)
	Total	1,283	775 (60.4)	716 (55.8)	813	211 (16.4)	23	1,222.5	1.9 (1.2–2.8)
**2**	5	400	234 (58.5)	216 (54.0)	239	36 (9.0)	3	419.1	0.7 (0.1–2.1)
	6	414	227 (54.8)	203 (49.0)	227	46 (11.1)	3	453.7	0.7 (0.1–1.9)
	7	270	143 (53.0)	158 (58.5)	163	36 (13.3)	6	285.6	2.1 (0.8–4.6)
	8	180	96 (53.3)	110 (61.1)	116	26 (14.4)	3	205.5	1.5 (0.3–4.3)
	9	207	112 (54.1)	87 (42.0)	123	31 (15.0)	2	209.4	1.0 (0.1–3.5)
	10	209	125 (59.8)	99 (47.4)	115	19 (9.1)	2	218.2	0.9 (0.1–3.3)
	Total	1,680	937 (55.8)	873 (52.0)	983	194 (11.5)	19	1,791.4	1.1 (0.6–1.7)
**3**	11	316	170 (53.8)	214 (67.7)	188	34 (10.8)	4	359.2	1.1 (0.3–2.9)
	12	375	202 (53.9)	205 (54.7)	207	35 (9.3)	2	441.6	0.5 (0.1–1.6)
	13	176	89 (50.6)	112 (63.6)	99	24 (13.6)	6	178.7	3.4 (1.2–7.3)
	Total	867	461 (53.2)	531 (61.2)	494	93 (10.7)	12	979.5	1.2 (0.6–2.1)
**4**	14	379	217 (53.3)	241 (63.6)	233	46 (12.1)	7	431.6	1.6 (0.7–3.3)
	15	262	149 (56.9)	158 (60.3)	181	36 (13.7)	1	251.0	0.4 (0.0–2.2)
	16	597	339 (56.8)	319 (53.4)	385	51 (8.5)	6	624.1	1.0 (0.4–2.1)
	17	399	223 (55.9)	240 (60.2)	229	34(8.5)	2	441.3	0.5 (0.1–1.6)
	Total	1,637	928 (56.7)	958 (58.5)	1,028	167 (10.2)	16	1,748.1	0.9 (0.5–1.5)
**5**	18	244	149 (61.1)	160 (65.6)	168	50 (20.5)	5	237.4	2.1 (0.7–4.9)
	19	140	79 (56.4)	97 (69.3)	88	28 (20.0)	3	148.2	2.0 (0.4–5.9)
	20	135	88 (65.2)	82 (60.7)	99	25 (18.5)	4	150.7	2.7 (0.7–6.8)
	21	222	133 (59.9)	130 (58.6)	150	49 (22.1)	4	221.7	1.8 (0.5–4.6)
	Total	741	449 (60.6)	469 (63.3)	505	152 (20.5)	16	757.9	2.1 (1.2–3.4)
**6**	22	428	225 (52.6)	254 (59.3)	254	50 (11.7)	4	496.8	0.8 (0.2–2.1)
	23	332	177 (53.3)	205 (61.7)	205	48 (14.5)	5	392.6	1.3 (0.4–3.0)
	24	771	428 (55.5)	448 (58.1)	458	79 (10.2)	7	922.8	0.8 (0.3–1.6)
	25	504	267 (53.0)	317 (62.9)	295	56 (11.1)	3	592.7	0.5 (0.1–1.5)
	Total	2,035	1,097 (53.9)	1,224 (60.1)	1,212	233 (11.4)	19	2,404.9	0.8 (0.5–1.2)
**7**	26	298	170 (57.0)	204 (68.5)	176	34 (11.4)	4	353.6	1.1 (0.3–2.9)
	27	456	258 (56.6)	284 (62.3)	278	36 (7.9)	4	523.8	0.8 (0.2–2.0)
	28	581	326 (56.1)	364 (62.7)	338	47 (8.1)	4	703.6	0.6 (0.2–1.5)
	29	251	132 (52.6)	176 (70.1)	148	28 (11.2)	3	300.8	1.0 (0.2–2.9)
	30	194	105 (54.1)	130 (67.0)	118	28 (14.4)	3	219.7	1.4 (0.3–4.0)
	Total	1,780	991 (55.7)	1,158 (65.1)	1,058	173 (9.7)	18	2,101.6	0.9 (0.5–1.4)
**8**	31	642	343 (53.4)	371 (57.8)	364	52 (8.1)	11	776.9	1.4 (0.7–2.5)
	32	185	95 (51.4)	92 (49.7)	108	16 (8.6)	3	221.9	1.4 (0.3–4.0)
	33	720	405 (56.2)	469 (65.1)	413	105 (14.6)	10	857.2	1.2 (0.6–2.1)
	Total	1,547	843 (54.5)	932 (60.2)	885	173 (11.2)	24	1,856.1	1.3 (0.8–1.9)
**9**	34	507	276 (54.4)	316 (62.3)	327	60 (11.8)	10	625.5	1.6 (0.8–2.9)
	35	340	194 (57.1)	220 (64.7)	240	52 (15.3)	5	345.5	1.4 (0.5–3.4)
	36	191	100 (52.4)	120 (62.8)	102	15 (7.9)	3	199.4	1.5 (0.3–4.4)
	37	75	43 (57.3)	41 (54.7)	51	11 (14.7)	2	77.7	2.6 (0.3–9.3)
	38	250	142 (56.8)	145 (58.0)	148	40 (16.0)	1	243.8	0.4 (0.0–2.3)
	39	76	47 (61.8)	39 (51.3)	44	10 (13.2)	1	78.1	1.3 (0.0–7.1)
	40	320	184 (57.5)	205 (64.1)	200	49 (15.3)	5	342.5	1.5 (0.5–3.4)
	41	180	107 (59.4)	106 (58.9)	108	11 (6.1)	0	180.4	—
	Total	1,939	1,093 (56.4)	1,192 (61.5)	1,220	248 (12.8)	27	2,092.9	1.3 (0.9–1.9)
**10**	42	569	312 (54.8)	426 (74.9)	360	45 (7.9)	4	680.4	0.6 (0.2–1.5)
	43	168	88 (52.4)	115 (68.5)	122	30 (17.9)	3	179.0	1.7 (0.3–4.9)
	44	142	84 (59.2)	86 (60.6)	88	18 (12.7)	4	163.5	2.4 (0.7–6.3)
	Total	879	484 (55.1)	627 (71.3)	570	93 (10.6)	11	1,022.9	1.1 (0.5–1.9)
**11**	45	100	64 (64.0)	53 (53.0)	66	27 (27.0)	4	85.7	4.7 (1.3–12.0)
	46	106	66 (62.3)	57 (53.8)	65	22 (20.8)	0	96.2	—
	Total	206	130 (63.1)	110 (53.4)	131	49 (23.8)	4	181.9	2.2 (0.6–5.6)

a Married refers to currently married individuals and includes married monogamous and married polygamous individuals.

b Calculated using Poisson regression and assuming that HIV seroconversion occurred at the midpoint of the follow-up interval.

We surveyed 75–771 eligible adults aged 15–49 y per community during RCCS R13, with 70% coverage of the censused target population (*n* = 14,594/21,275). There were 1,786 HIV-seropositive men and women who participated in RCCS R13, of whom 189 were incident cases. Among the HIV-seroprevalent individuals in this survey (*n* = 1,597), 1,345 had participated in a prior RCCS survey round, and 26.2% (*n* = 352/1,345) of these individuals were on ART. Among the HIV-seroprevalent men and women entering into the RCCS for the first time (*n* = 252), none were on ART. Overall, HIV seropositivity was 12.2% (*n* = 1,786/14,594), and incidence was 1.2 per 100 person-years (95% CI: 1.0–1.3) (Table 1). Individuals who were lost to follow-up during the interval prior to RCCS R13 (30.9% attrition) were significantly more likely to be unmarried (Poisson unadjusted relative risk [RR] = 1.59; 95% CI: 1.53–1.66) and significantly more likely to be less than age 25 y (RR = 1.62; 95% CI: 1.56–1.69) than those who remained in the study. Persons lost to follow-up were marginally more likely to be male (RR = 1.07; 95% CI: 1.03–1.12) and HIV seropositive (RR = 1.09; 95% CI: 1.02–1.16).

### Spatial Clustering of HIV-Seropositive Individuals

#### Spatial clustering of HIV-seropositive individuals within households

**Figure 2:**
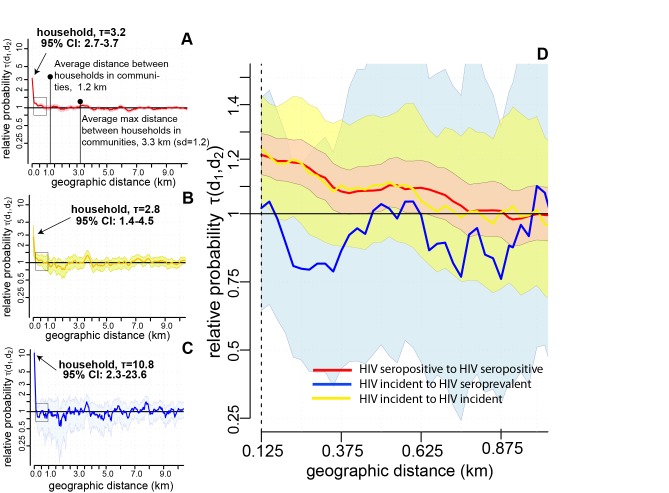
Spatial clustering of HIV-seropositive persons within households (0 km) and in geographic windows of 250 m up to 10 km (the first window is 10–250 m, and windows are centered every 50 m starting at 125 m). Spatial clustering analyses show whether HIV prevalence or incidence is elevated within certain distances of other HIV-seropositive persons. We define the spatial clustering of HIV-seropositive individuals as τ(*d*_1_,*d*_2_), the relative probability that an HIV-seropositive person resides within a distance window, *d*_1_ to *d*_2_, from another HIV-seropositive person compared to the probability that any individual is HIV seropositive in the entire study population. Where spatial clustering exists, values of τ(*d*_1_,*d*_2_) exceed one. Shaded areas show the 95% bootstrapped confidence intervals for spatial clustering estimates. (A) The spatial clustering between HIV-seropositive persons (prevalent or incident cases with other prevalent or incident cases; red). (B) The spatial clustering of HIV-seroincident cases with ART-naïve HIV-seroprevalent persons (yellow). (C) The spatial clustering of HIV-seroincident cases with other HIV-seroincident cases (blue). (D) A blowup of the area where significant extra-household spatial clustering (<500 m) was identified among all HIV-seropositive persons (marked with black box in [A–C]). Data are shown only up to 10 km (no significant spatial clustering was observed beyond this distance).

We observed strong spatial clustering of HIV-seropositive individuals within households (Figure 2A–2C). The probability that a participant living in the same household as an HIV-seropositive participant was also HIV seropositive was 3.2 (95% CI: 2.7–3.7) times greater than the probability that any RCCS participant was HIV seropositive (shown in red, Figure 2A). Even stronger household spatial clustering was observed among HIV-incident cases: the probability that a participant living with an HIV-incident case was also HIV incident was 10.8 (95% CI: 2.3–23.6) times the probability that any participant was an HIV-incident case (shown in blue, Figure 2C).

#### Spatial clustering of HIV-seropositive individuals within communities

We explored whether there was spatial clustering of HIV-seropositive individuals outside of households at distances up to 30 km. We found statistically significant though weaker spatial clustering of HIV-seropositive persons outside of households. Compared to all study participants, persons living 10–250 m from a HIV-seropositive participant were 1.22 (95% CI: 1.14–1.29) times as likely to be HIV seropositive themselves, and those living 250–500 m away were 1.08 (95% CI: 1.00–1.17) times as likely to be HIV seropositive (Figure 2A and 2D).

We also examined whether incident cases spatially clustered with other HIV-incident and -seroprevalent cases outside the household, since spatial clustering among all HIV-seropositive persons may reflect historic rather than recent patterns of HIV transmission. In contrast, we observed no statistically significant extra-household spatial clustering of HIV-incident cases with other incident or seroprevalent cases (Figure 2B and 2D), though incident cases appeared to weakly cluster with seroprevalent cases at geographic distances less than 500 m (shown in yellow, Figure 2B and 2D). There was no significant spatial clustering beyond 500 m in any spatial analyses and no significant intra-household or extra-household spatial clustering between HIV-incident and HIV-seroprevalent persons on ART (Figure S4).

### HIV Phylogenetics within and across Communities

Viral sequence data for the *gag* and *env* genes were obtained for 1,099/1,434 (76.6%) HIV-seropositive participants who were not on ART at the time of the RCCS R13 survey (Table S2), including 164 of 189 (86.7%) incident cases (Table S3). On average, 15 (range 3–24) viral sequences were retrieved from HIV-incident cases, and 85 (range 15–143) sequences were retrieved from HIV-prevalent cases per geographic region. Sequences were predominantly HIV-1 subtypes A1 or D, and both subtypes were found in all communities. Of those participants with sequence information in both gene regions (*n* = 842/1,099), 21.1% (*n* = 178/842) did not share the same HIV-1 subtype in *gag* and *env* genes. No statistically significant differences were found between HIV-infected individuals from whom viral sequences were obtained (in either or both genes) and those from whom no viral genetic data were obtained for duration of the participant's infection (prevalent or incident), gender, marital status, or geographic region of residence. However, there was a significant decrease in the number of sequences obtained with each increasing year of age (either gene: RR = 0.988; 95% CI: 0.980–0.995; both genes: RR = 0.990; 95% CI: 0.982–1.00).

#### Genetic relatedness of HIV viruses within households

Our study population included 165 epidemiologically linked couples where both partners had participated in RCCS R13 and were HIV seropositive and not on ART at the time of the survey. Twenty-five percent (*n* = 42/165) of these couples included at least one incident case (both partners were HIV incident in 9/42 incident couples). Sequence information was available for at least one gene region (either *gag* or *env*) in 63.6% (*n* = 105/165) of epidemiologically linked couples, including 76.2% (*n* = 32/42) of those with one or more incident cases (*n* = 7/9, 77.7% of those where both cases were incident). Ninety-nine percent (*n* = 104/105) of epidemiologically linked pairs with sequence data shared a household, including all 32 incident couples.

**Figure 3:**
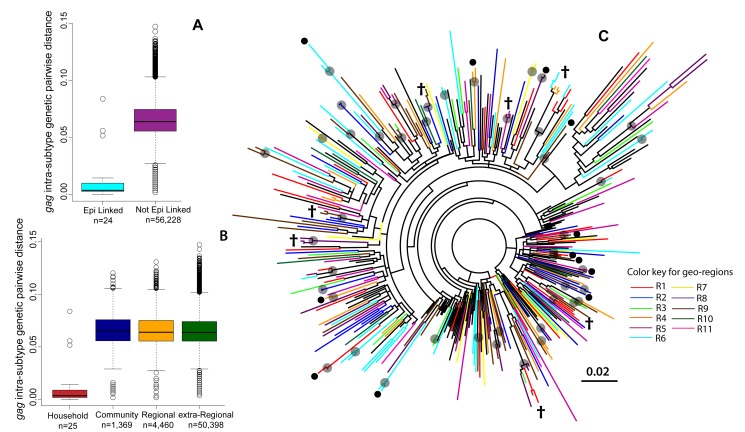
Maximum likelihood phylogenetic analyses of the HIV-1 *gag* gene. (A) Boxplots of the intra-subtype *gag* genetic pairwise distances for epidemiologically linked (Epi linked) incident couples (i.e., at least one member of the couple was an incident case) and for all epidemiologically unlinked incident pairs of individuals in RCCS R13. (B) Boxplots of intra-subtype *gag* genetic pairwise distances by the geographic distance between the incident pair. (C) A ML phylogenetic tree (radial) of HIV-1 subtype A *gag* sequences from HIV-seroprevalent (*n* = 245) and HIV-incident (*n* = 55) cases, where taxa are colored by the geographic region from which they were isolated. Reference strains (*n* = 87) are in black. Grey circles indicate nodes with bootstrap support of ≥70%; black circles indicate intra-household clusters; † indicates an intra-household virus also sharing a cluster with at least one other household. Additional radial and rectangular phylogenetic trees for HIV-1 subtypes A, D, and C for *gag* and *env* genes are included in Figures S5, S6, S7, S8, S9, S10, S11, S12, S13.

The median genetic distance between epidemiologically linked couples with an incident case was 0.4% in *gag* (*n* = 24/32, interquartile range [IQR]: 0.3%–0.9%) and 0.9% in *env* (*n* = 27/32, IQR: 0.4%–1.3%; Figure 3A). All of these epidemiologically linked couples (*n* = 32/32) shared the same viral subtype in one or both genes, but only 71.9% (*n* = 23/32) shared a phylogenetic cluster in the ML trees in at least one gene region. In comparison, the median intra-subtype genetic distance between epidemiologically linked HIV-seroprevalent partners was 1.3% in *gag* (*n* = 47/73, IQR: 0.9%–2.2%) and 2.7% in *env* (*n* = 55/73, IQR: 2.0%–4.2%), and only 38.4% (*n* = 28/73) of these couples phylogenetically clustered in at least one gene region.

There were 12 households where sequence data were available for two persons who were not epidemiologically linked, all of whom were HIV-seroprevalent pairs. Median intra-subtype genetic distance in these pairs was 6.4% in *gag* (*n* = 7/12, IQR: 3.0%–7.5%) and 9.4% in *env* (*n* = 10/12, IQR: 7.0%–10.7%), and only one pair phylogenetically clustered within the ML trees. A detailed summary of the HIV genetic data for all of the 105 epidemiologically linked couples with HIV sequence data is included in Table S4.

#### Genetic relatedness of HIV viruses within and across communities

Shown in Figure 3B is the distribution of intra-subtype genetic distances in the *gag* gene for incident couples (i.e., one sequence obtained from an incident case) sharing the same community (median = 6.3%; IQR: 5.4%–7.3%). This distribution was nearly identical to that seen within geographic regions (median = 6.4%; IQR: 5.4%–7.4%) and across all communities (median = 6.4%; IQR: 5.5%–7.3%). Similar distributions were observed in the *env* gene (data not shown).

Limited geographic structure was observed in ML phylogenetic trees, regardless of the viral subtype or gene region examined (Figures 3C and S5, S6, S7, S8, S9, S10, S11, S12, S13). More detailed phylogenetic trees, including information on both HIV status (i.e., incident or prevalent) and community of residence, showed that viral sequences from HIV-incident cases were distributed throughout the phylogenetic tree, with no apparent regard to community or geographic region of primary residence (Figures S5, S6, S7, S8, S9, S10, S11, S12, S13).

Two participants sharing a phylogenetic cluster suggests—because of our strict cluster definition—that they are separated by a relatively short and recent chain of transmission. Only 19.0% (209/1,099) of HIV-infected participants in RCCS R13 shared a phylogenetic cluster with at least one other RCCS study participant in either the *gag* or *env* genes. A total of 95 phylogenetic clusters were identified across all ML phylogenetic trees (*n* = 209 individuals; Tables 2 and S4). The majority of clusters included only two (86.3%, *n* = 82/95) or three HIV-infected persons (9.5%, *n* = 9/95). We also identified four additional phylogenetic clusters, of which two clusters contained four individuals each (2.1%, *n* = 2/95) and two clusters contained five individuals each (2.1%, *n* = 2/95). None of the identified phylogenetic clusters contained a reference sequence, and 40.0% (*n* = 38/95) contained at least one incident case, encompassing 50 incident cases in total (Table 2).

**Table 2:** Characteristics of 95 phylogenetic clusters identified in maximum likelihood phylogenetic analyses (HKY-85) of 915 *gag* sequences and 1,026 *env* sequences obtained from 1,099 HIV-infected participants in RCCS R13.

Cluster Characteristic^a^	Reference Unit	All Phylogenetic Clusters (*n* = 95)	Phylogenetic Clusters with Incident Case(s) (*n* = 38)
Cluster size distribution	Number of participants in cluster (frequency)	2 (82), 3 (9), 4 (2), 5 (2)	2 (30), 3 (5), 4 (1), 5 (2)
Clusters containing only incident cases	Number of clusters (percent of total clusters)	6 (6.3)	6 (15.8)
Household clusters^b^	Number of clusters (percent of total clusters)	42 (44.2)	18 (47.4)
Intra-community clusters^c^	Number of clusters (percent of total clusters)	15 (15.8)	7 (18.4)
Cross-community clusters^d^	Number of clusters (percent of total clusters)	38 (40.0)	13 (34.2)
Cross-regional clusters^e^	Number of clusters (percent of total clusters)	18 (18.9)	6 (15.8)

a Categories not mutually exclusive.

b Refers to clusters of two individuals who shared the same household.

c Refers to clusters of two or more individuals who spanned households but shared the same community.

d Refers to clusters of two or more individuals who spanned households and communities.

e Refers to clusters of two or more individuals who spanned households, communities, and geographic regions.

Almost half of all phylogenetic clusters identified (44.2%, *n* = 42/95) were household pairs of two (63 prevalent cases; 21 incident cases). Of the 53 clusters that contained participants who spanned households (*n* = 53/95), 38 clusters crossed community boundaries (71.7%). These 38 cross-community clusters included 28 pairs (47 prevalent cases; nine incident cases); seven triplets (18 prevalent cases; three incident cases), two clusters of size four (four prevalent cases; four incident cases), and one cluster of size five (one prevalent case; four incident cases). Nearly half of the cross-community clusters (47.4%, *n* = 18/38) also spanned geographic regions. Community clusters (*n* = 15/53) included 12 pairs (19 prevalent cases; five incident cases), two triplets (four prevalent cases; two incident cases), and one cluster of size five (three prevalent cases; two incident cases). When analyses were restricted to only those clusters containing at least one incident case (*n* = 38/95), similar geographic patterns were observed (Table 2).

There were six phylogenetic clusters that contained only incident cases (6.3%, *n* = 6/95), of which five contained a single household pair (ten incident cases) and one contained two household pairs (four incident cases). Our definition of a phylogenetic cluster may have precluded the identification of some transmission chains; however, in sensitivity analyses the proportion of clusters with more than one household that crossed community boundaries was robust to the strictest (66.7%, *n* = 18/27 crossed community boundaries) and most relaxed (74.0%, *n* = 77/104 crossed community boundaries) phylogenetic cluster definitions assessed (Table S5). A detailed summary of each of the 95 phylogenetic clusters identified is included in Table S5.

### Probable Infection from Household, Community, and Extra-Community Sources

A total of 11,992 recent sexual partners were self-reported by 5,368 women and 4,152 men who were HIV seronegative at a previous study visit (Table 3). Of these self-reported partners, 42.1% (*n* = 5,043) could be epidemiologically linked to another RCCS participant who participated in RCCS R13 or a previous survey round. Ninety-six percent (*n* = 5,159/5,368) of women reported only one sexual partner in the last 12 mo, compared to 59.2% of men (*n* = 2,458/4,152) (Table S7). Of enumerated self-reported partners, 63.0% (*n* = 7,549/11,992) held primary residence within the participant's household, 19.5% (*n* = 2,342/11,992) were within the participant's community but outside of the household, and 17.5% (*n* = 2,101/11,992) had a primary residence outside of the participant's community (Table S8). Household partnerships were almost always stable partnerships (i.e., 99% were marital or long-term consensual unions), whereas partnerships outside of the household were usually not stable (95%; Table S8). The majority of extra-household sexual partners were reported by unmarried persons (*n* = 2,895/4,443, 65.2%).

**Table 3:** Descriptive characteristics of HIV-seronegative and -incident participants in egocentric partner analysis (*n* = 9,520).

Characteristic	Women (*n* = 5,368)		Men (*n* = 4,152)	
	HIV-Seronegative *n* (Percent)	HIV-Incident *n* (Percent)	HIV-Seronegative *n* (Percent)	HIV-Incident *n* (Percent)
**Total population**	5,258 (98.0)	110 (2.0)	4,073 (98.1)	79 (1.9)
**Age in years**				
15–19 y	290 (5.5)	6 (5.5)	320 (7.9)	2 (2.5)
20–24 y	921 (17.5)	25 (22.7)	677 (16.6)	12 (15.2)
25–29 y	1,287 (24.5)	25 (22.7)	820 (20.1)	20 (25.3)
30–34 y	1,095 (20.8)	31 (28.2)	792 (19.4)	22 (27.8)
35–39 y	710 (13.5)	13 (11.8)	681 (16.7)	14 (17.7)
40+ y	955 (18.2)	10 (9.1)	783 (19.2)	9 (11.4)
**Marital status**				
Never married	590 (11.2)	19 (17.2)	906 (22.2)	11 (13.9)
Unmarried, previously married	779 (14.8)	34 (30.9)	311 (7.3)	14 (17.7)
Married, not polygamous	2,935 (55.8)	39 (35.5)	2,418 (59.4)	45 (57.0)
Married, polygamous^a^	954 (18.1)	18 (16.4)	438 (10.8)	9 (11.4)
**Number of self-reported recent sexual partners (last 12 mo)** b				
1	5,060 (96.2)	99 (90.0)	2,425 (59.5)	33 (41.8)
2	189 (3.6)	8 (7.2)	1,165 (28.6)	30 (40.0)
3–4	9 (0.2)	3 (2.7)	483 (11.9)	16 (20.2)
**Locations of self-reported recent sexual partners**				
Household only	3,907 (74.3)	59 (53.6)	1,918 (47.1)	27 (34.2)
Community only	554 (10.5)	12 (10.9)	559 (13.7)	13 (16.5)
Extra-community only	644 (12.2)	33 (30.0)	371 (9.1)	5 (6.3)
Household and community	73 (1.4)	2 (1.8)	564 (13.8)	10 (12.7)
Household and extra-community	44 (0.8)	1 (0.9)	411 (10.1)	14 (17.7)
Community and extra-community	33 (0.6)	3 (2.7)	159 (3.9)	5 (6.3)
Household, community, and extra-community	3 (0.1)	0 (0.0)	91 (2.2)	5 (6.3)
**One or more self-reported recent sexual partners with the following HIV serostatus** c				
HIV seronegative	2,274 (43.2)	10 (9.1)	2,361 (58.0)	16 (20.2)
ART naïve, HIV incident	16 (0.3)	9 (8.2)	8 (0.2)	9 (11.4)
ART naïve, HIV seroprevalent	101 (1.9)	18 (16.4)	91 (2.2)	19 (24.1)
Using ART^d^, HIV seroprevalent	14 (0.2)	0 (0.0)	14 (0.3)	1 (1.2)^e^
Missing HIV serostatus	2,928 (55.7)	76 (69.0)	2,640 (63.3)	64 (81.0)

a “Married, polygamous” for women refers to a woman in a marital relationship with a man who has multiple wives.

b Self-reported sexual partners from the egocentric partnership block of the RCCS study questionnaire (records up to four partners in the last 12 mo).

c Categories not mutually exclusive (i.e., participants may report multiple partners with different HIV serostatus).

d Partners were considered to be on ART if they were using ART for 50% or more of the corresponding index participant's time at risk.

e Partner on ART for 58% of the newly infected index participant's time at risk (previous to current survey interval).

#### Attributable fractions of HIV infections from household-based transmission

Using the egocentric partner data, we estimated that 39.0% (95% CI: 32.3%–43.9%) of 189 incident cases were infected by a household sexual partner (Table 4). Those with an incident household partner (*n* = 9 household pairs) had an estimated 26.0% (95% CI: 13.4%–45.0%) probability of acquiring HIV from that partner (Table 5). In 20.6% of cases where infection was attributed to a household partner with known HIV status, that partner was him/herself an incident case. There were 38 incident events among 250 individuals in a stable sexual partnership with an ART-naïve HIV-seroprevalent partner. After accounting for risk from other self-reported partners and unknown sources, we estimate that the probability of transmission from these seroprevalent household partners not on ART was 15.3% (95% CI: 10.9%–20.6%). Among at-risk individuals who had an HIV-seroprevalent partner who was on ART for 50% or more of the risk interval (*n* = 29), only one became HIV-infected; and there were no infections among the 27 with partners who were on ART for 60% or more of the interval.

**Table 4:** Attributable HIV transmissions by geographic location of sexual partner and gender of newly infected participant (estimated from egocentric transmission model).

HIV Status of Partner	Residential Location of Partner with Respect to Incident Case	Men (*n* = 79)		Women (*n* = 110)		Overall (*n* = 189)	
		Attributable Fraction	95% CI	Attributable Fraction	95% CI	Attributable Fraction	95% CI
ART naïve, HIV seroprevalent	Household	21.0%	17.7%–22.8%	16.1%	14.6%–16.4%	18.1%	16.4%–19.1%
ART naïve, HIV incident	Household	4.1%	1.3%–7.6%	5.2%	2.7%–7.3%	4.7%	4.2%–4.8%
Missing HIV status	Household	15.8%	10.1%–21.5%	16.6%	10.9%–20.9%	16.2%	11.6%–20.1%
Missing HIV status	Extra-household, intra-community	21.7%	15.2%–27.9%	10.2%	6.4%–13.6%	15.0%	11.1%–18.5%
Missing HIV status	Extra-household, extra-community	16.1%	7.8%–22.3%	30.6%	27.3%–33.6%	24.6%	20.1%–28.0%
Unknown contacts/sources	Unknown location	21.5%	12.7%–32.9%	21.4%	14.6%–30.0%	21.4%	14.8%–29.6%
	Household total^a^					39.0%	32.3%–43.9%
	Extra-household total^b^					39.5%	33.9%–42.3%

a Estimate includes infections attributable to ART-naïve HIV-prevalent and -incident cases and household partners with missing HIV status.

b Estimate includes extra-household, intra-community, and extra-community partners.

**Table 5:** Probability of HIV infection by partner type over 18-mo study interval.

Partner Type	Probability of HIV-Infection	95% CI
ART-naïve, HIV-incident partner^a^	26.0%	13.4%–43.0%
ART-naïve, HIV-seroprevalent partner^a^	15.3%	10.9%–20.6%
Household partner with unknown HIV serostatus	1.1%	0.7%–1.7%
Community partner with unknown HIV serostatus	1.3%	0.8%–2.0%
Extra-community partner with unknown HIV serostatus, for women	4.2%	3.0%–5.9%
Extra-community partner with unknown HIV serostatus, for men	0.9%	0.3%–1.8%
Unknown contacts/undisclosed partners	0.3%	0.2%–0.5%

a 99% of partnerships were intra-household.

The HIV status for the suspected index partner in 16.2% (95% CI: 11.6%–20.1%) of household transmissions was unknown.

#### Attributable fractions of HIV infections from community, extra-community, and unknown sources

Infections from self-reported extra-household partners were estimated to account for 39.5% of new cases (95% CI: 33.9%–42.3%), of which the majority (62.1%, 95% CI: 54.9%–69.7%) were from self-reported partners outside the community (Table 4). Where the specific location of these self-reported extra-community sexual partners was known (68%), 50% lived outside of the Rakai District and were geographically dispersed throughout Uganda (Figure 1A). While men were 1.8 times more likely to disclose an extra-community partner than women (1,061/4,152 versus 761/5,368; 95% CI: 1.7–2.0), those women who reported an extra-community partner had higher odds of HIV acquisition from that self-reported partner than men who reported an extra-community partner (odds ratio = 5.0; 95% CI: 2.2–14.1). Acquisition from unknown sources accounted for 21.4% of total infections (95% CI: 14.8%–29.6%), although the individual probability of such infections was low (0.3%; 95% CI: 0.2–0.5).

#### Sensitivity analysis

Sensitivity analyses were conducted to determine the robustness of the parameter estimates in Table 5 to underreporting and misreporting of self-reported sexual partnerships. In simulations where 10% of self-reported partnerships were considered unreported, the median bias in parameter estimates for the transmission model was less than 10% of the width of the 95% confidence interval in all cases except for the probability of infection from an unnamed source (ρ), which increased as expected. Moreover, all 95% CIs included the original point estimate, with the exception of ρ, which differed as expected. In simulations where 10% of extra-household partnerships were considered to have a misreported geographic relationship with the study participant (i.e., extra-community partners were reported as community partners or vice versa), the median bias of each parameter estimate was less than 10% of the reported 95% CI width, and 97% or more of the 95% CIs from simulated estimates included the estimate from the original data.

## Discussion

Using spatial statistics, viral phylogenetics, and egocentric transmission models we find evidence that extra-community HIV introductions are frequent, and likely play a significant role in sustaining ongoing HIV incidence in rural Rakai, Uganda. We estimate that viral introductions combined with intra-household transmission account for the majority of incident infections in this HIV-endemic region, though our data also suggest that community-based sexual networks play a critical part in HIV spread. Our results underscore the complexities of HIV epidemic dynamics and sexual networks in rural Uganda and have important implications for the design and implementation of CRCTs and HIV prevention programs.

**Figure 4:**
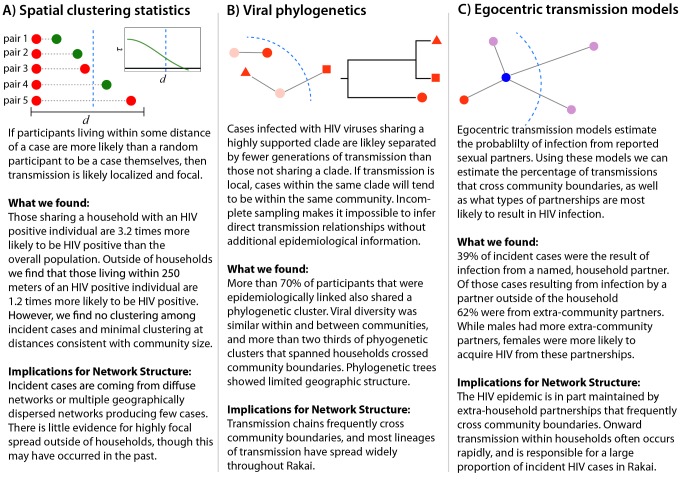
Summary of inferential methods and study results and conclusions. The dotted blue line represents the border of a hypothetical community.

Each of the analyses used illuminates a different aspect of HIV transmission networks, and together they provide a powerful framework for understanding the spatial scale and structure of HIV transmission networks (Figure 4). Spatial analyses reveal whether HIV incidence or prevalence is elevated in close proximity to HIV-infected persons, but cannot distinguish whether spatially related cases are part of the same sexual network. Viral phylogenetics provides insight into the relationship between spatial and viral genetic similarity; however, high mutation rates and sparse sampling of networks make it impossible to definitively link cases to an infecting source. Egocentric transmission models relate the geographic distribution of personal sexual networks to individuals' risk of HIV infection, but give minimal insight into global network structure.

All three analyses suggest that frequent HIV introductions into communities play a critical role in ongoing HIV incidence in rural Rakai, Uganda (Figure 4). They show limited spatial clustering of HIV cases outside of households, multiple circulating HIV viruses within communities, and a significant proportion of incidence resulting from extra-community partnerships. Together, our data imply that there are frequent viral introductions into communities, followed by onward transmission within households (where we estimate over 1/3 of transmission occurs) and within small intra-community sexual networks. These findings do not rule out an important role for community-level sexual networks in the Rakai HIV epidemic, but do suggest that local HIV epidemics are not sustained through community-based viral transmission alone. Furthermore, they highlight the risks of applying the results of sexually transmitted infection studies in urban areas outside of Africa (e.g., studies showing strong spatial clustering of gonorrhea cases in Baltimore [26]) to HIV control efforts within rural Africa.

In this prospective population-based cohort, intra-household HIV transmission was common, accounting for approximately 39% of new incident cases. This fraction is within the range of that previously estimated in 18 sub-Saharan African countries [27], but lower than the 55%–97% estimated in Zambia and Rwanda [28], both based on cross-sectional Demographic and Health Surveys (DHS). Hence, targeting treatment to stable HIV-discordant couples could prevent substantial numbers of new infections, but the effectiveness of this strategy is largely contingent on the rapid identification and treatment of HIV-infected index partners. Consistent with other studies [29],[30], we found that the highest risk of HIV acquisition was within the first 18 mo of an index partner's infection. Chronically HIV-infected individuals also posed substantial, though lower, risk to their uninfected partners; however, ART appeared to eliminate this risk entirely. The strong protective effect of ART observed in this population-based study corroborates the findings from the HPTN 052 clinical trial and other observational studies of HIV transmission in Africa [25]. Though no individuals in our study acquired HIV from an identifiable HIV-seroprevalent partner on ART, we cannot rule out the possibility that non-identifiable sexual partners of incident cases were taking ART at the time of transmission.

While intra-household transmission was common, it is extra-household transmission that determines the geographic scale of HIV epidemics. Here we estimate that more than half of all household introductions were the result of extra-community partnerships, with a wide geographic range of sexual partner networks. Fifty percent of extra-community partners had a primary residence outside of Rakai, including major urban centers in Uganda (i.e., Kampala and Masaka). Within the Rakai District, but outside of the RCCS target area, there are fishing communities along Lake Victoria where HIV prevalence is extremely high (∼40% in data from an unpublished pilot study of 2,106 individuals in fishing communities in the Rakai District). Preliminary data show that men from these high-risk fishing communities frequently travel to RCCS communities, which may in part explain the high rate of HIV infection we observed among unmarried women with extra-community partners. Mobility has long been associated with HIV transmission in Africa [31],[32], though how exactly it relates to local epidemic dynamics, including the persistence of viral transmission in African contexts, remains understudied. Studies of other infectious diseases and network simulations suggest that such long distance “jumps”—even when infrequent—can facilitate persistence of infection within broader contact networks [33]–[35].

We did not measure the impact of local treatment as prevention in this study; however, our results provide insight into the mechanisms and upper limits of its effectiveness when implemented only locally, given the relative fractions of community and cross-community HIV spread. Our results suggest that community-based ART programs could have a major impact on African epidemics, but also highlight the need to target extra-community sources of HIV infection. Viral introductions could be reduced either through wider spread coverage of ART among HIV-infected persons or through prevention interventions that provide direct protection to uninfected individuals (e.g., male circumcision or pre-exposure prophylaxis). Targeting interventions that provide direct protection to those most likely to have extra-community partners may be an important addition to local HIV control strategies.

Viral introductions pose significant challenges to epidemiological studies of HIV risk and prevention. Exposure misclassification may be common when using community viral load or other aggregated community-level measures of individual HIV risk [36],[37]. Similarly, in the case of CRCTs, indirect intervention effects may be obscured when cross-community transmissions are frequent [13]. Incorporating phylogenetics and detailed information on individual partnerships into study design may facilitate interpretation of results from community-based studies of treatment as prevention, including the upcoming HPTN 071 and Mochudi Prevention Project trials [11],[12].

Our study had several limitations. While RCCS demographics, including age distribution, marital status, and sexual behaviors, are largely representative of the broader Uganda population (Table S9) [38], our results may not be generalizable and suggest the need to study the spatial dynamics of HIV in other settings. In particular, uptake of HIV preventive services may be greater in RCCS communities, which could bias our estimates of per partnership risk if local HIV-infected partners were less likely to be infectious than partners outside of Rakai. A comparison of male circumcision prevalence in our study population versus in the general Ugandan population, as sampled in the DHS survey in 2011, revealed that the male circumcision rate was higher among RCCS participants than among DHS participants (39.4% versus 26.8%), though HIV prevalence and ART use among HIV-infected persons was similar between RCCS and DHS sampled populations (Table S9). We also considered newly enrolled HIV-seropositive persons to be HIV seroprevalent, potentially underestimating the effect of early HIV infections on transmission. Overrepresentation of particular types of partnerships in our sample could also have biased results. For example, oversampling of household partners could lead to overestimation of the importance of household transmission; however, the proportions of men and women who were married in RCCS were similar to those reported in the Ugandan DHS, and household partners were not selectively recruited over community partners [38]. Another limitation is that we identified the geographic sources of HIV infection from self-reported sexual partner data that may be inaccurate. The presence of HIV-incident cases for which no possible infecting partner could be determined indicates that some sexual partners were unreported. If these unreported sources of infection were evenly split between community and extra-community partners (as opposed to following the distribution in the data), our estimate of the percentage of extra-household transmission due to community partners would increase from 38% to 45%. Furthermore, sensitivity analyses show that randomly unreported partnerships or randomly misreported community status would not substantially bias the results. However, systematic biases in partnership reporting could bias our results.

A notable of strength of our study was its prospective population-based study design, which captured a representative sample of the sexually active adult population in rural Rakai and yielded a sampling fraction of local sexual networks (∼70% of the censused population) in the 46 surveyed communities. Individuals lost to follow-up during the interval of observation were more likely to be unmarried and younger than those who remained in the study. Such missing persons may be more mobile and at higher HIV risk. If so, our estimate of the frequency of cross-community transmission is likely an underestimate of the true value. Despite limited losses to follow-up and a high sampling fraction of the primary geographic unit of analysis (the community), we still observed minimal phylogenetic clustering between HIV sequences obtained from the same community, which limited our ability to identify HIV transmission chains using molecular epidemiological methods. Low levels of phylogenetic clustering are not uncommon in studies of HIV epidemics, particularly phylogenetic studies of heterosexual HIV transmission networks [39],[40]. Still, we were surprised to find so many singleton lineages within communities, given study participation rates. While it is true that we may have undersampled local sexual networks to some extent, high viral diversity within communities, coupled with a lack of spatial clustering outside of households and a high probability of infection from extra-community partners, implies that the limited phylogenetic clustering is a reflection of frequent viral introductions, at least in part. Intra-host HIV evolutionary dynamics, including HIV co-infection and rapid HIV genetic drift, also may have obscured the identification of HIV transmission chains using our phylogenetic approaches.

Taken together, our analyses reveal a complex picture of HIV dynamics in rural Uganda, and suggest that incidence is in part sustained through repeated introductions of HIV, with frequent intra-household transmission and some onward transmission through small intra-community networks. It remains unknown whether these patterns reflect broader source–sink dynamics, in which localized key populations, such as fishing communities with high HIV prevalence, may have a major effect on regional HIV transmission dynamics. HIV introductions present a challenge to local HIV control programs and CRCTs, necessitating a commitment to widespread combination HIV prevention in sub-Saharan Africa, and a deeper understanding of the extra-community partnerships that reintroduce infection into rural populations.

## Supporting Information

Figure S1 **The geographic scale of RCCS communities.** Communities are color-coded according to their RCCS geographic region (see Figure 1 for color key). The means for the average and maximum geographic distances between households within a community (across all communities) are marked with dotted red lines. The size of the dot is proportional to the size of the surveyed population/community size.
(PDF)

Figure S2 **Phylogenetic analyses of** ***gag*** **and** ***env*** **genes for specimens that underwent repeated viral RNA extraction and PCR testing.** Repeated viral RNA extractions and PCR testing was performed for a sample of patient specimens for *gag* (*n* = 26) (A) and *env* (*n* = 46) (B) to assess the reliability of our laboratory methods. Sequences were compared using neighbor-joining trees (1,000 bootstrap replicates). Trees were constructed separately for each gene region using a Tamura-Nei model of nucleotide substitution. Results of the phylogenetic analyses showed that the laboratory methods yielded reliable sequence information: sequences obtained from the same individual always clustered together.
(TIF)

Figure S3 **Genetic pairwise distances in** ***gag*** **and** ***env*** **genes for epidemiologically linked HIV-infected couples where at least one partner was an HIV-incident case.** Figures show only those incident couples who shared a monophyletic clade in a ML tree with 70% or greater bootstrap support. These distributions were used to determine the genetic distance thresholds for phylogenetic cluster analyses.
(TIFF)

Figure S4 **Spatial clustering of HIV-seroprevalent persons on ART with HIV-incident cases within households (0 km) and in geographic windows of 250 m up to 10 km (centered every 50 m beginning at 125 m).** Spatial clustering, τ(*d*_1_,*d*_2_), shown in black, is the relative probability that an HIV-seroprevalent person on ART resides within a distance range, *d*_1_ to *d*_2_, from an incident case compared to the probability that any individual participant is an incident case. The shaded area is the bootstrapped 95% confidence interval (1,000 iterations).
(TIF)

Figure S5 **Maximum likelihood tree (rectangular) of** ***gag*** **HIV-1 subtype A sequences.** Taxa are labeled using participant gender/geographic region/community/household. Reference sequences (*n* = 88) are in black, and only bootstrap values ≥50% are shown. Color corresponds to the geographic region.
(PDF)

Figure S6 **Maximum likelihood tree (radial) of** ***gag*** **HIV-1 subtype D sequences.** Color corresponds to geographic region (see Figure 1 key). Reference sequences (*n* = 57) are in black. Grey circles indicate nodes with bootstrap support of ≥70%; black circles indicate intra-household clusters; † indicates intra-household viruses also sharing a cluster with at least one other household.
(PDF)

Figure S7 **Maximum likelihood tree (rectangular) of** ***gag*** **HIV-1 subtype D sequences.** Taxa are labeled using participant gender/geographic region/community/household. Reference sequences (*n* = 57) are in black, and only bootstrap values ≥50% are shown. Color corresponds to the geographic region.
(PDF)

Figure S8 **Maximum likelihood tree (rectangular) of** ***gag*** **HIV-1 subtype C sequences.** Taxa are labeled using participant gender/geographic region/community/household. Reference sequences (*n* = 37) are in black, and only bootstrap values ≥50% are shown. Color corresponds to the geographic region.
(PDF)

Figure S9 **Maximum likelihood tree (radial) of** ***env*** **HIV-1 subtype A sequences.** Color corresponds to geographic region (see Figure 1 key). Reference sequences (*n* = 107) are in black. Grey circles indicate nodes with bootstrap support of ≥70%; black circles indicate intra-household clusters; † indicates intra-household viruses also sharing a cluster with at least one other household.
(PDF)

Figure S10 **Maximum likelihood tree (rectangular) of** ***env*** **HIV-1 subtype A sequences.** Taxa are labeled using participant gender/geographic region/community/household. Reference sequences (*n* = 107) are in black, and only bootstrap values ≥50% are shown. Color corresponds to the geographic region.
(PDF)

Figure S11 **Maximum likelihood tree (radial) of** ***env*** **HIV-1 subtype D sequences.** Color corresponds to geographic region (see Figure 1 key). Reference sequences (*n* = 70) are in black. Grey circles indicate nodes with bootstrap support of ≥70%; black circles indicate intra-household clusters; † indicates intra-household viruses also sharing a cluster with at least one other household.
(PDF)

Figure S12 **Maximum likelihood tree (rectangular) of** ***env*** **HIV-1 subtype D sequences.** Taxa are labeled using participant gender/geographic region/community/household. Reference sequences (*n* = 70) are in black, and only bootstrap values ≥50% are shown. Color corresponds to the geographic region.
(PDF)

Figure S13 **Maximum likelihood tree (rectangular) of** ***env*** **HIV-1 subtype C sequences.** Taxa are labeled using participant gender/geographic region/community/household. Reference sequences (*n* = 37) are in black, and only bootstrap values ≥50% are shown. Color corresponds to the geographic regions.
(PDF)

Table S1 **Accession numbers for Los Alamos National Laboratory HIV Sequence Database reference sequences used for maximum likelihood and Bayesian phylogenetic analyses.** This table includes the accession numbers, geographic location, year of collection, and HIV-1 subtype for each *gag* and *env* reference sequence used in phylogenetic analyses.
(DOCX)

Table S2 **Summary of HIV sequences from 1,434 HIV-1-seropositive participants in RCCS R13.** Table includes the HIV-1 group M subtype assignment of isolated viruses in *gag* and *env* genes.
(DOCX)

Table S3 **Summary of HIV sequences obtained from 189 HIV-1-incident participants in RCCS R13.** Table includes the HIV-1 group M subtype assignment of isolated viruses in *gag* and *env* genes.
(DOCX)

Table S4 **Summary phylogenetic data (HIV subtype, genetic pairwise distance, and phylogenetic clustering results) for the 105 epidemiologically linked incident couples with phylogenetic data in** ***gag*** **and/or** ***env*** **gene regions.**
(DOCX)

Table S5 **Detailed summary data for each of the 95 phylogenetic clusters identified in maximum likelihood phylogenetic trees (HKY-85 model).**
(DOCX)

Table S6 **Sensitivity analyses of phylogenetic clustering results to choice of evolutionary model and bootstrap and genetic distance thresholds.** Phylogenetic cluster analyses were conducted at 70%, 80%, 90%, and 99% bootstrap thresholds, with and without genetic distance cutoffs under the HKY-85 and GTR+I+G models of evolution. We present the cluster summary data shown in Table 2 under these different evolutionary models and genetic distance and bootstrap threshold criteria.
(DOCX)

Table S7 **Numbers of recent sexual partners self-reported by 9,520 HIV-seronegative and -incident participants in egocentric analysis by gender and marital status of the study participant.**
(DOCX)

Table S8 **Summary of self-reported sexual partner data from 9,520 HIV-seronegative and -incident participants in egocentric analysis by gender of the study participant and geographic location of the sexual partner.**
(DOCX)

Table S9 **Comparison of demographics and sexual behaviors (percent distribution) between RCCS study population (RCCS R13, 2008–2009) and the surveyed population in the 2011 Ugandan Demographic and Health Survey.**
(DOCX)

## Abbreviations

ART: antiretroviral therapy
CRCT: community-randomized controlled trial
DHS: Demographic and Health Surveys
GTR+I+G: general time reversible model with gamma distributed rate heterogeneity and a proportion of invariable sites
HIV: human immunodeficiency virus
IQR: interquartile range
ML: maximum likelihood
RCCS: Rakai Community Cohort Study
RCCS R13: Rakai Community Cohort Study survey round 13
RR: relative risk
