# Supplementary figures and images for: The Role of Viral Introductions in Sustaining Community-Based HIV Epidemics in Rural Uganda: Evidence from Spatial Clustering, Phylogenetics, and Egocentric Transmission Models
Source: PLoS Med. 2014 Mar 4;11(3):e1001610. doi: 10.1371/journal.pmed.1001610 (PMC3942316; doi:10.1371/journal.pmed.1001610)

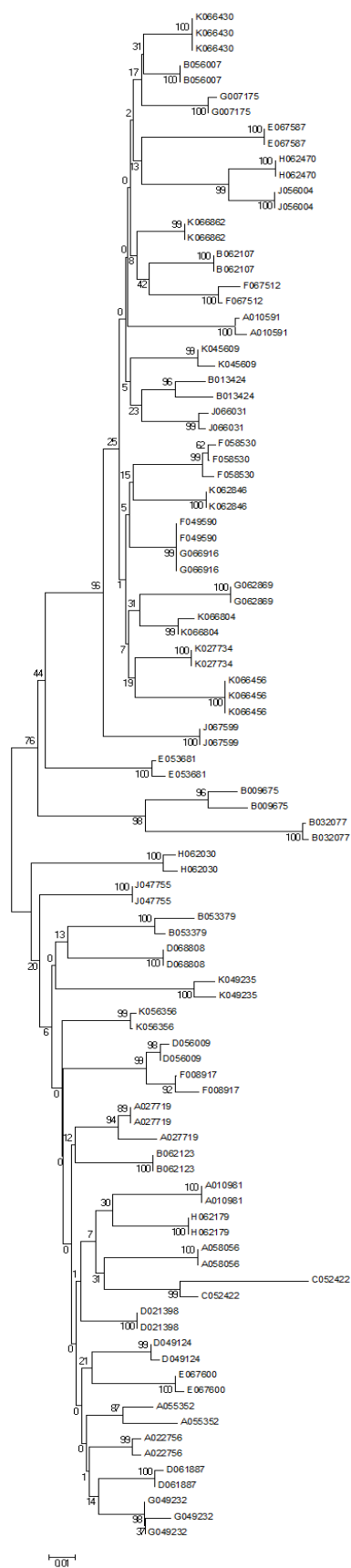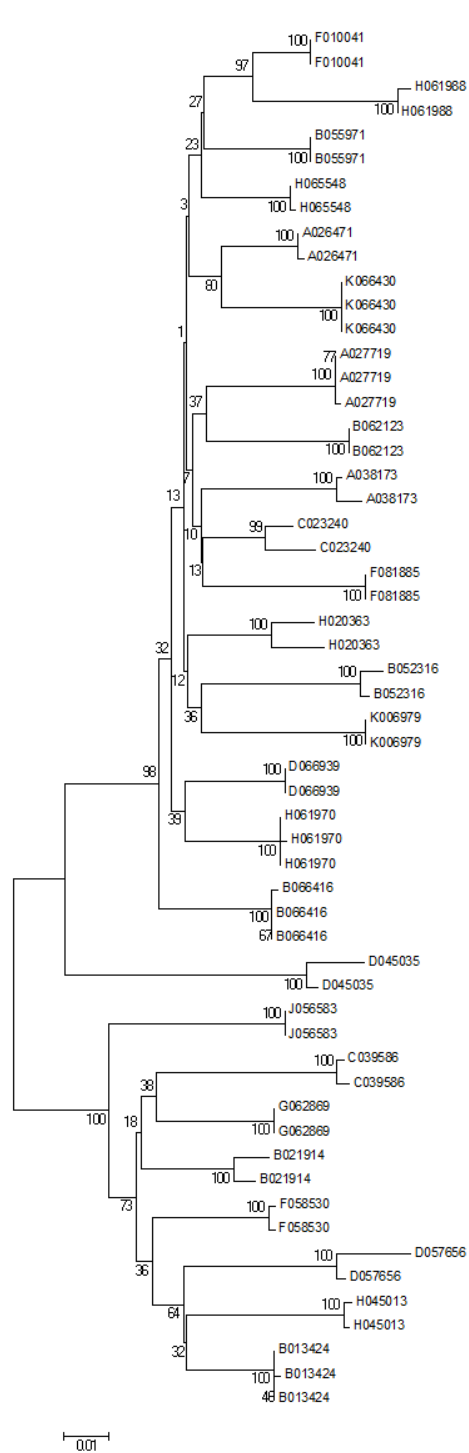

Supplement: Figure S1 — The geographic scale of RCCS communities. Communities are color-coded according to their RCCS geographic region (see Figure 1 for color key). The means for the average and maximum geographic distances between households within a community (across all communities) are marked with dotted red lines. The size of the dot is proportional to the size of the surveyed population/community size. (PDF) [file pmed.1001610.s001.pdf]

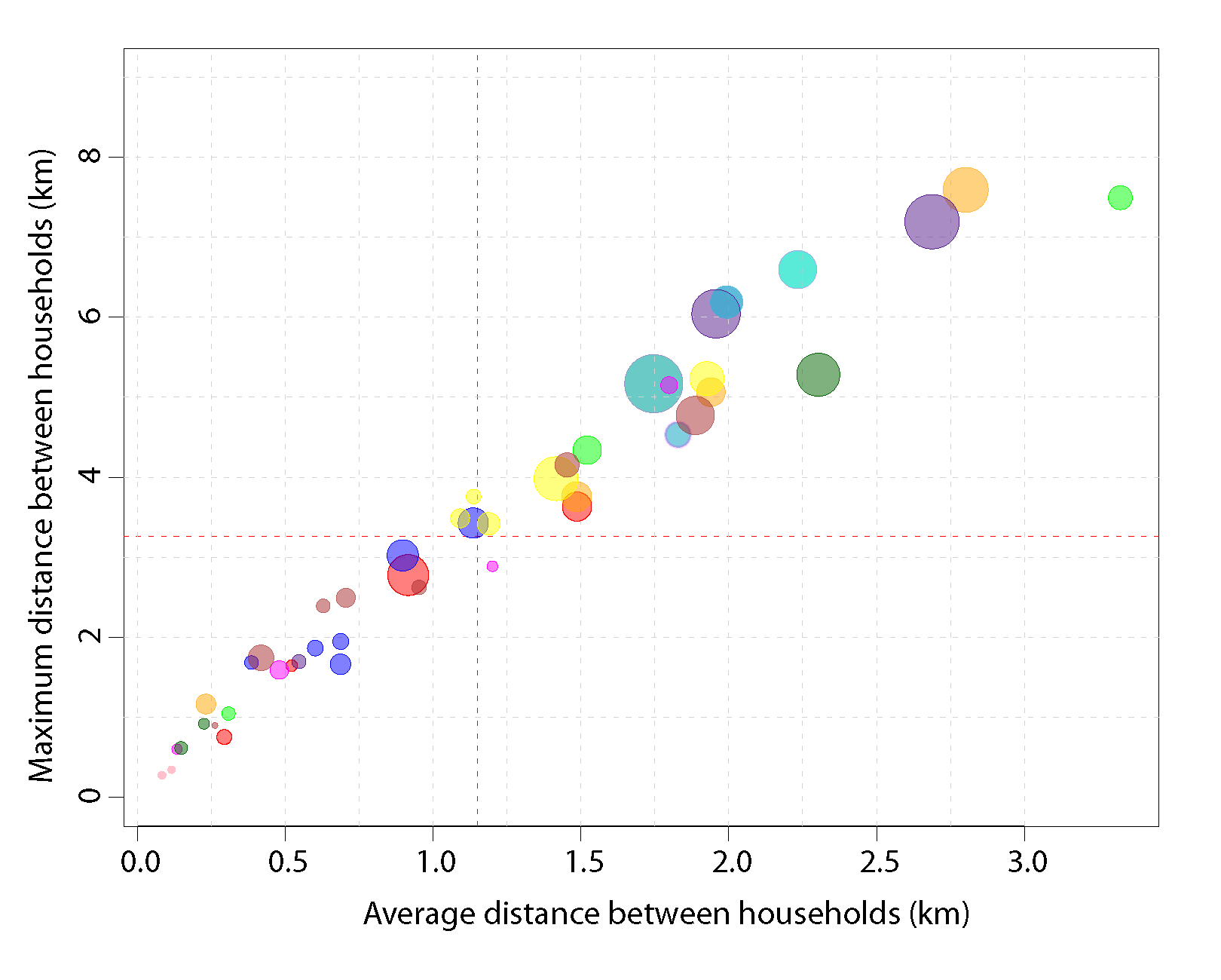

Supplement: Figure S2 — Phylogenetic analyses of gag and env genes for specimens that underwent repeated viral RNA extraction and PCR testing. Repeated viral RNA extractions and PCR testing was performed for a sample of patient specimens for gag (n = 26) (A) and env (n = 46) (B) to assess the reliability of our laboratory methods. Sequences were compared using neighbor-joining trees (1,000 bootstrap replicates). Trees were constructed separately for each gene region using a Tamura-Nei model of nucleotide substitution. Results of the phylogenetic analyses showed that the laboratory methods yielded reliable sequence information: sequences obtained from the same individual always clustered together. (TIF) [file pmed.1001610.s002.tif]

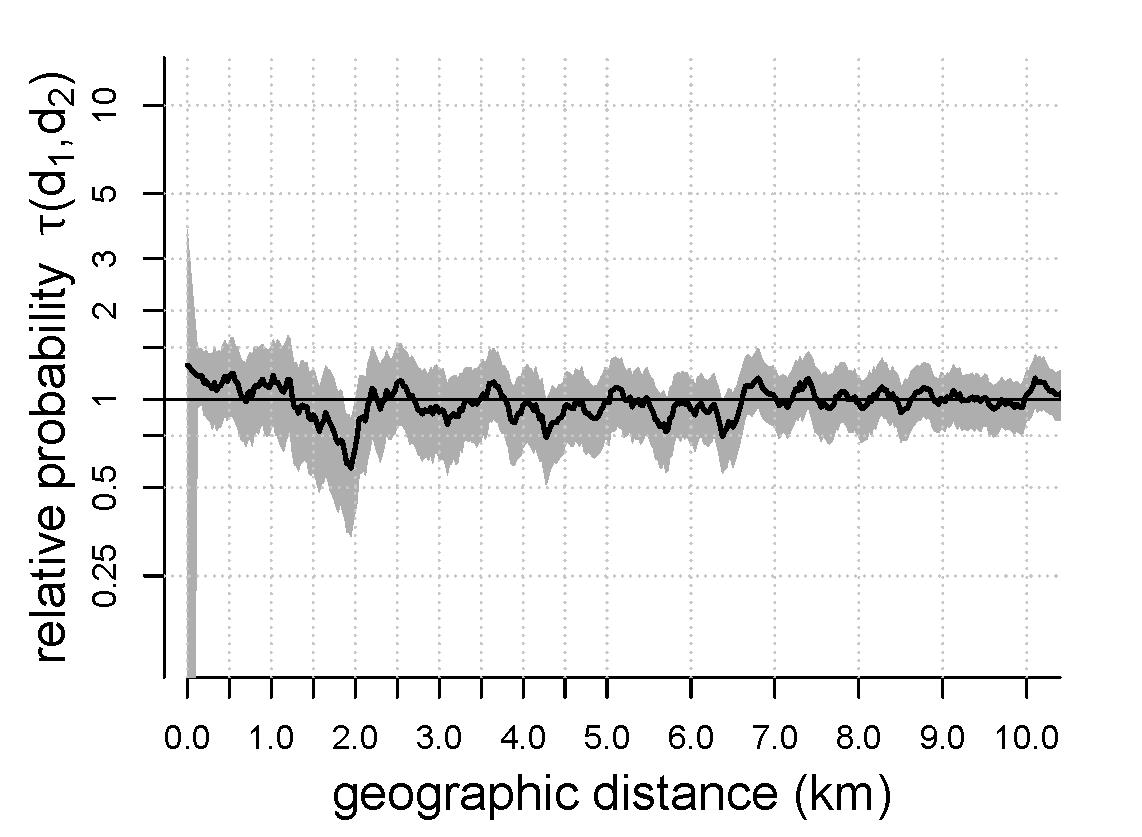

Supplement: Figure S4 — Spatial clustering of HIV-seroprevalent persons on ART with HIV-incident cases within households (0 km) and in geographic windows of 250 m up to 10 km (centered every 50 m beginning at 125 m). Spatial clustering, τ(d 1,d 2), shown in black, is the relative probability that an HIV-seroprevalent person on ART resides within a distance range, d 1 to d 2, from an incident case compared to the probability that any individual participant is an incident case. The shaded area is the bootstrapped 95% confidence interval (1,000 iterations). (TIF) [file pmed.1001610.s004.tif]

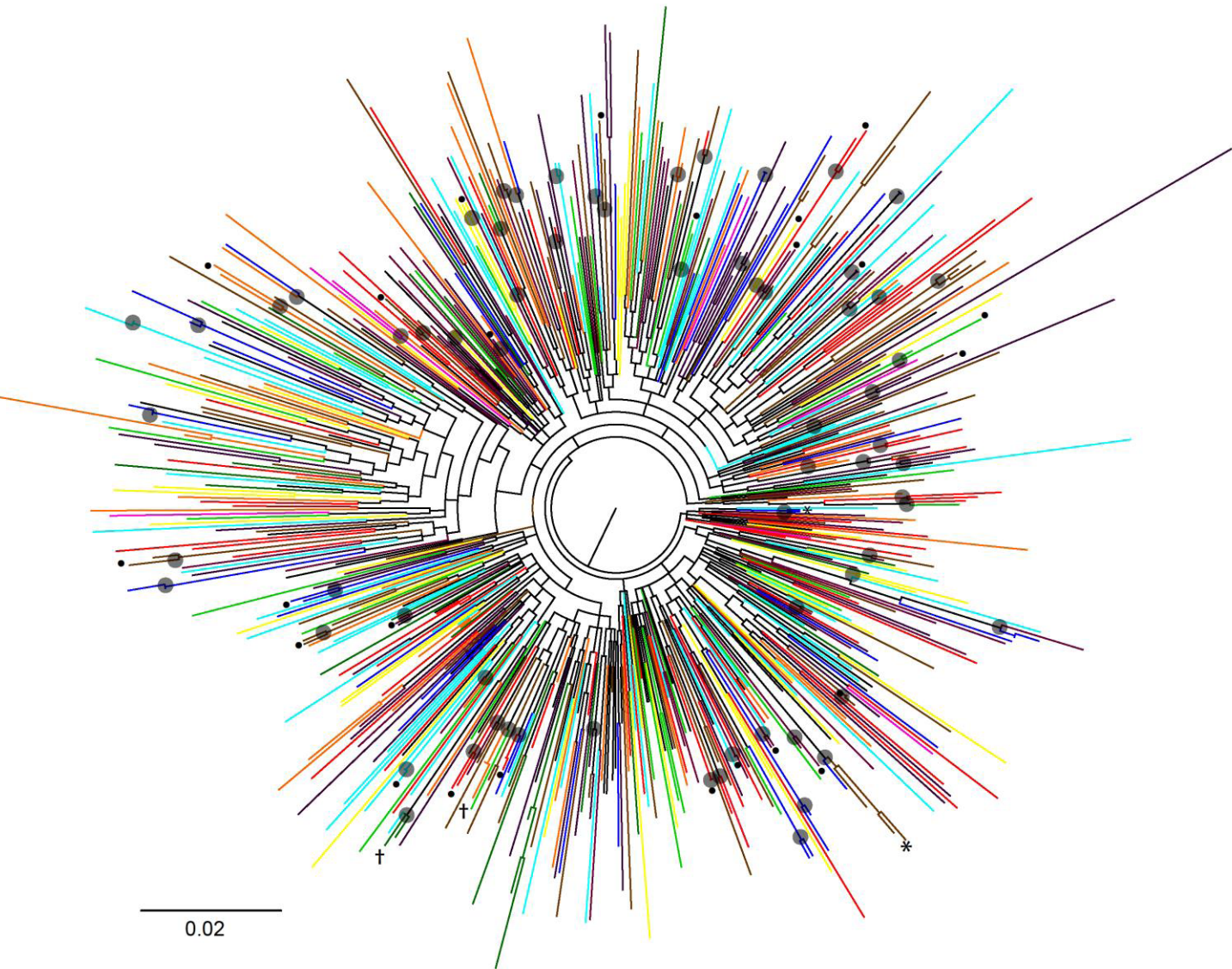

Supplement: Figure S6 — Maximum likelihood tree (radial) of gag HIV-1 subtype D sequences. Color corresponds to geographic region (see Figure 1 key). Reference sequences (n = 57) are in black. Grey circles indicate nodes with bootstrap support of ≥70%; black circles indicate intra-household clusters; † indicates intra-household viruses also sharing a cluster with at least one other household. (PDF) [file pmed.1001610.s006.pdf]

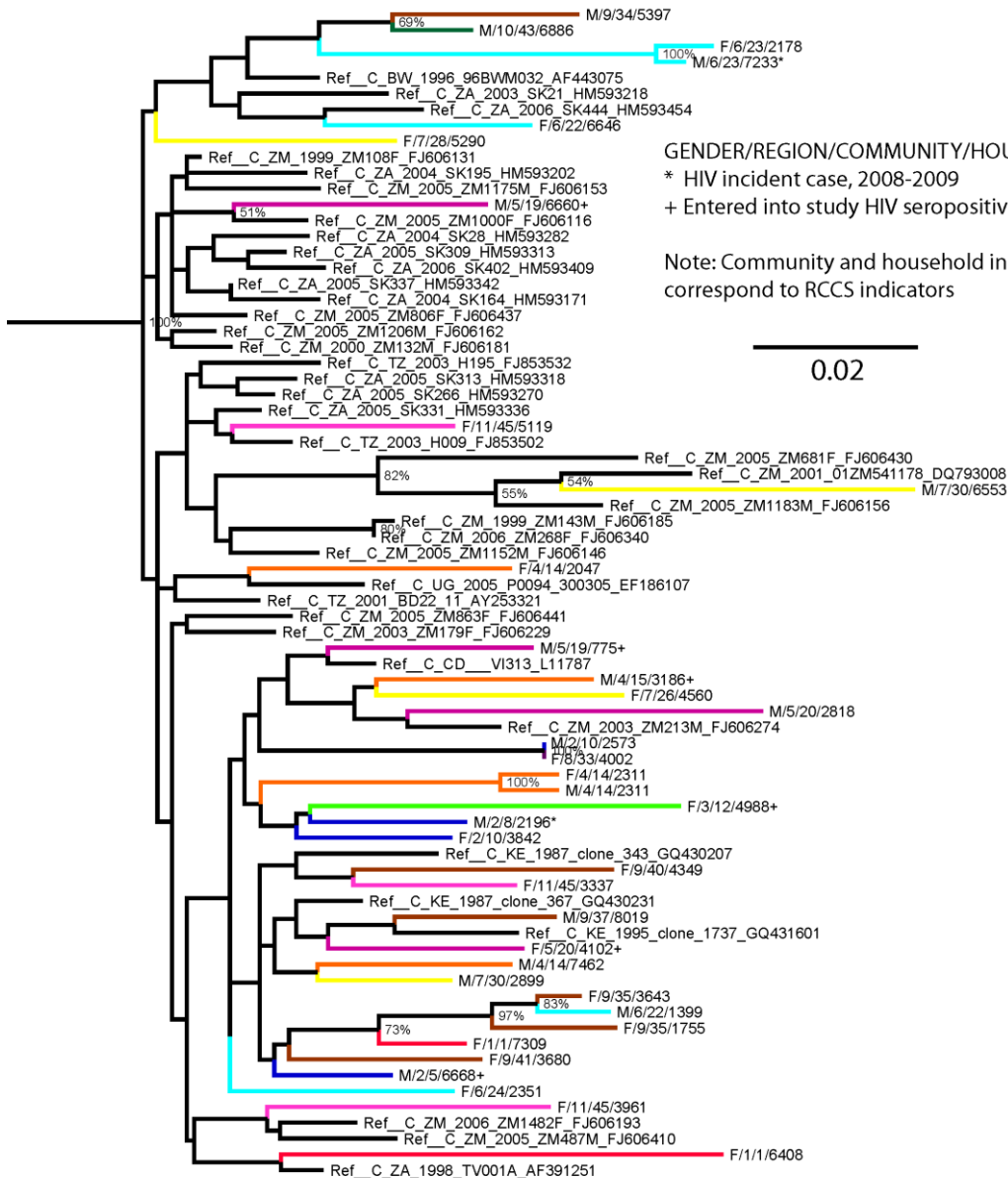

Supplement: Figure S8 — Maximum likelihood tree (rectangular) of gag HIV-1 subtype C sequences. Taxa are labeled using participant gender/geographic region/community/household. Reference sequences (n = 37) are in black, and only bootstrap values ≥50% are shown. Color corresponds to the geographic region. (PDF) [file pmed.1001610.s008.pdf]

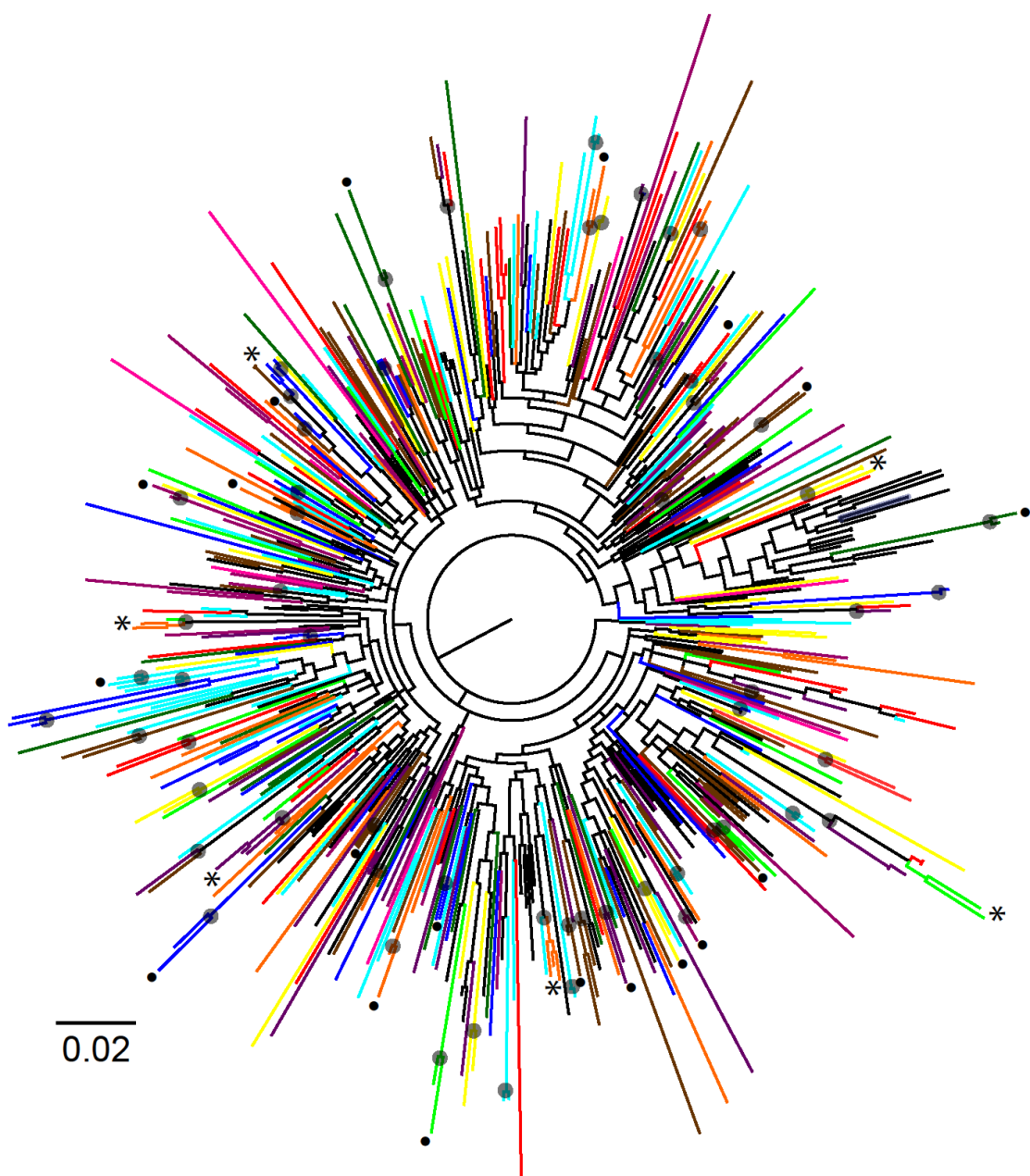

Supplement: Figure S9 — Maximum likelihood tree (radial) of env HIV-1 subtype A sequences. Color corresponds to geographic region (see Figure 1 key). Reference sequences (n = 107) are in black. Grey circles indicate nodes with bootstrap support of ≥70%; black circles indicate intra-household clusters; † indicates intra-household viruses also sharing a cluster with at least one other household. (PDF) [file pmed.1001610.s009.pdf]

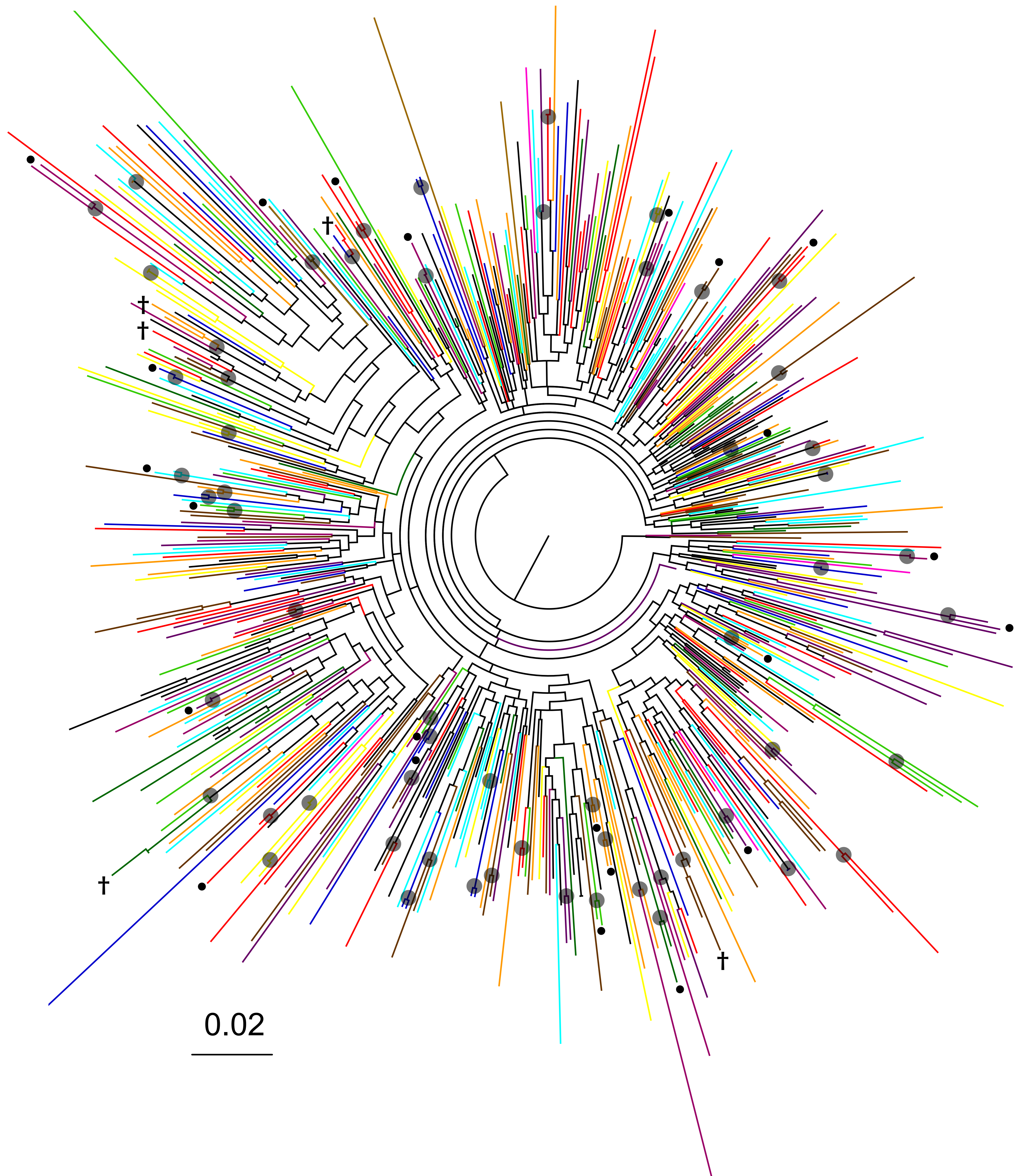

0.02

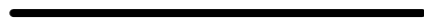

Supplement: Figure S11 — Maximum likelihood tree (radial) of env HIV-1 subtype D sequences. Color corresponds to geographic region (see Figure 1 key). Reference sequences (n = 70) are in black. Grey circles indicate nodes with bootstrap support of ≥70%; black circles indicate intra-household clusters; † indicates intra-household viruses also sharing a cluster with at least one other household. (PDF) [file pmed.1001610.s011.pdf]
