# Supplementary material for: The Role of Viral Introductions in Sustaining Community-Based HIV Epidemics in Rural Uganda: Evidence from Spatial Clustering, Phylogenetics, and Egocentric Transmission Models
Source: PLoS Med. 2014 Mar 4;11(3):e1001610. doi: 10.1371/journal.pmed.1001610 (PMC3942316; doi:10.1371/journal.pmed.1001610)
Supplement: Figure S5 — Maximum likelihood tree (rectangular) of gag HIV-1 subtype A sequences. Taxa are labeled using participant gender/geographic region/community/household. Reference sequences (n = 88) are in black, and only bootstrap values ≥50% are shown. Color corresponds to the geographic region. (PDF) [file pmed.1001610.s005.pdf]

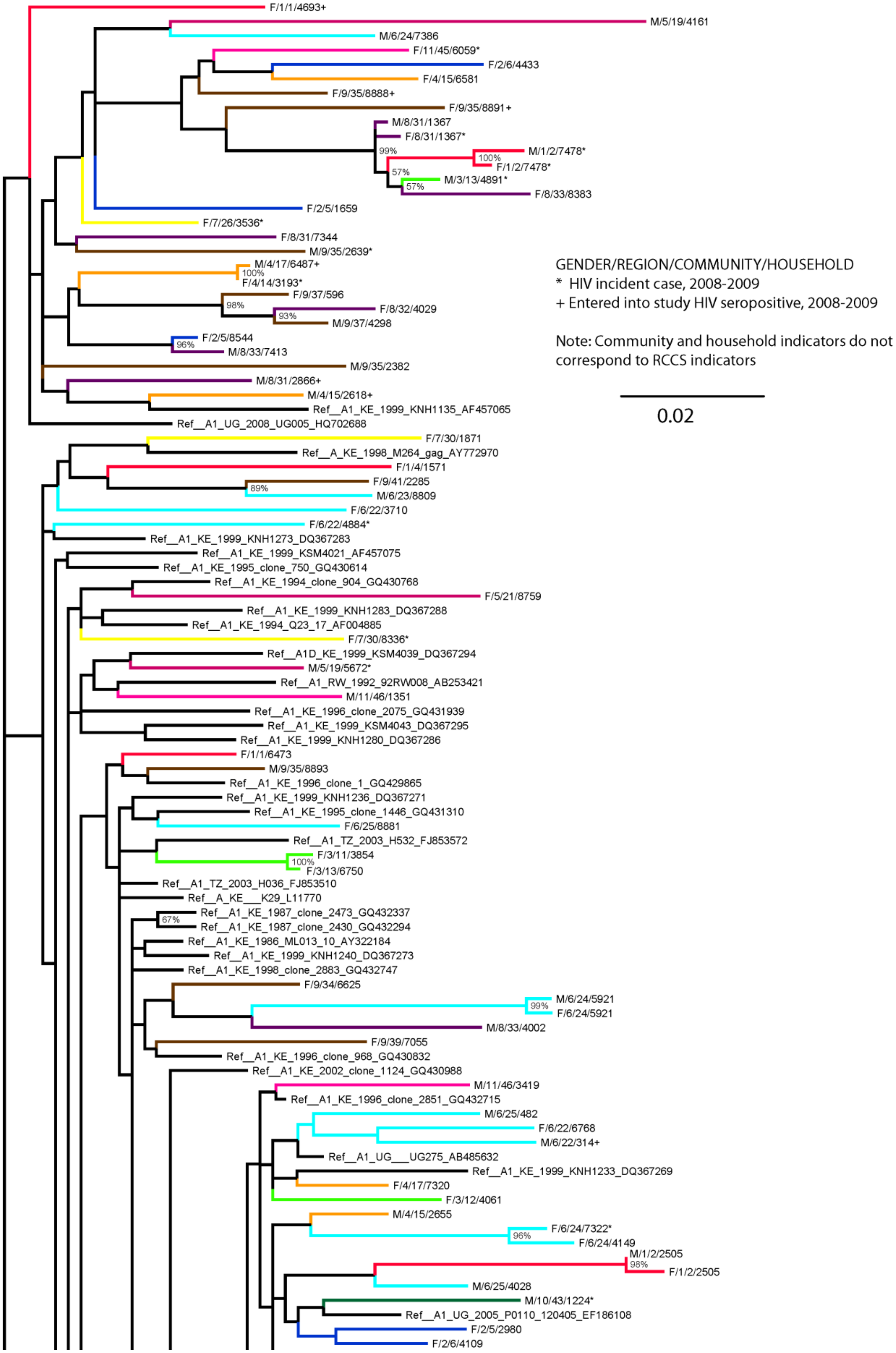
F/1/1/4693+
M/5/19/4161
M/6/24/7386
F/11/45/6059*
F/2/6/4433
F/4/15/6581
F/9/35/8888+
F/9/35/8891+
M/8/31/1367
F/8/31/1367*
99%
M/1/2/7478*
100%
F/1/2/7478*
57%
M/3/13/4891*
57%
F/8/33/8383
F/2/5/1659
F/7/26/3536*
F/8/31/7344
M/9/35/2639*
M/4/17/6487+
100%
F/4/14/3193*
F/9/37/596
98%
F/8/32/4029
93%
M/9/37/4298
F/2/5/8544
96%
M/8/33/7413
M/9/35/2382
M/8/31/2866+
M/4/15/2618+
Ref__A1_KE_1999_KNH1135_AF457065
Ref__A1_UG_2008_UG005_HQ702688
GENDER/REGION/COMMUNITY/HOUSEHOLD
* HIV incident case, 2008-2009
+ Entered into study HIV seropositive, 2008-2009
Note: Community and household indicators do not correspond to RCCS indicators
0.02
F/7/30/1871
Ref__A_KE_1998_M264_gag_AY772970
F/1/4/1571
F/9/41/2285
89%
M/6/23/8809
F/6/22/3710
F/6/22/4884*
Ref__A1_KE_1999_KNH1273_DQ367283
Ref__A1_KE_1999_KSM4021_AF457075
Ref__A1_KE_1995_clone_750_GQ430614
Ref__A1_KE_1994_clone_904_GQ430768
F/5/21/8759
Ref__A1_KE_1999_KNH1283_DQ367288
Ref__A1_KE_1994_Q23_17_AF004885
F/7/30/8336*
Ref__A1D_KE_1999_KSM4039_DQ367294
M/5/19/5672*
Ref__A1_RW_1992_92RW008_AB253421
M/11/46/1351
Ref__A1_KE_1996_clone_2075_GQ431939
Ref__A1_KE_1999_KSM4043_DQ367295
Ref__A1_KE_1999_KNH1280_DQ367286
F/1/1/6473
M/9/35/8893
Ref__A1_KE_1996_clone_1_GQ429865
Ref__A1_KE_1999_KNH1236_DQ367271
Ref__A1_KE_1995_clone_1446_GQ431310
F/6/25/8881
Ref__A1_TZ_2003_H532_FJ853572
F/3/11/3854
100%
F/3/13/6750
Ref__A1_TZ_2003_H036_FJ853510
Ref__A_KE___K29_L11770
Ref__A1_KE_1987_clone_2473_GQ432337
67%
Ref__A1_KE_1987_clone_2430_GQ432294
Ref__A1_KE_1986_ML013_10_AY322184
Ref__A1_KE_1999_KNH1240_DQ367273
Ref__A1_KE_1998_clone_2883_GQ432747
F/9/34/6625
M/6/24/5921
99%
F/6/24/5921
M/8/33/4002
F/9/39/7055
Ref__A1_KE_1996_clone_968_GQ430832
Ref__A1_KE_2002_clone_1124_GQ430988
M/11/46/3419
Ref__A1_KE_1996_clone_2851_GQ432715
M/6/25/482
F/6/22/6768
M/6/22/314+
Ref__A1_UG___UG275_AB485632
Ref__A1_KE_1999_KNH1233_DQ367269
F/4/17/7320
F/3/12/4061
M/4/15/2655
F/6/24/7322*
96%
F/6/24/4149
M/1/2/2505
98%
F/1/2/2505
M/6/25/4028
M/10/43/1224*
Ref__A1_UG_2005_P0110_120405_EF186108
F/2/5/2980
F/2/6/4109

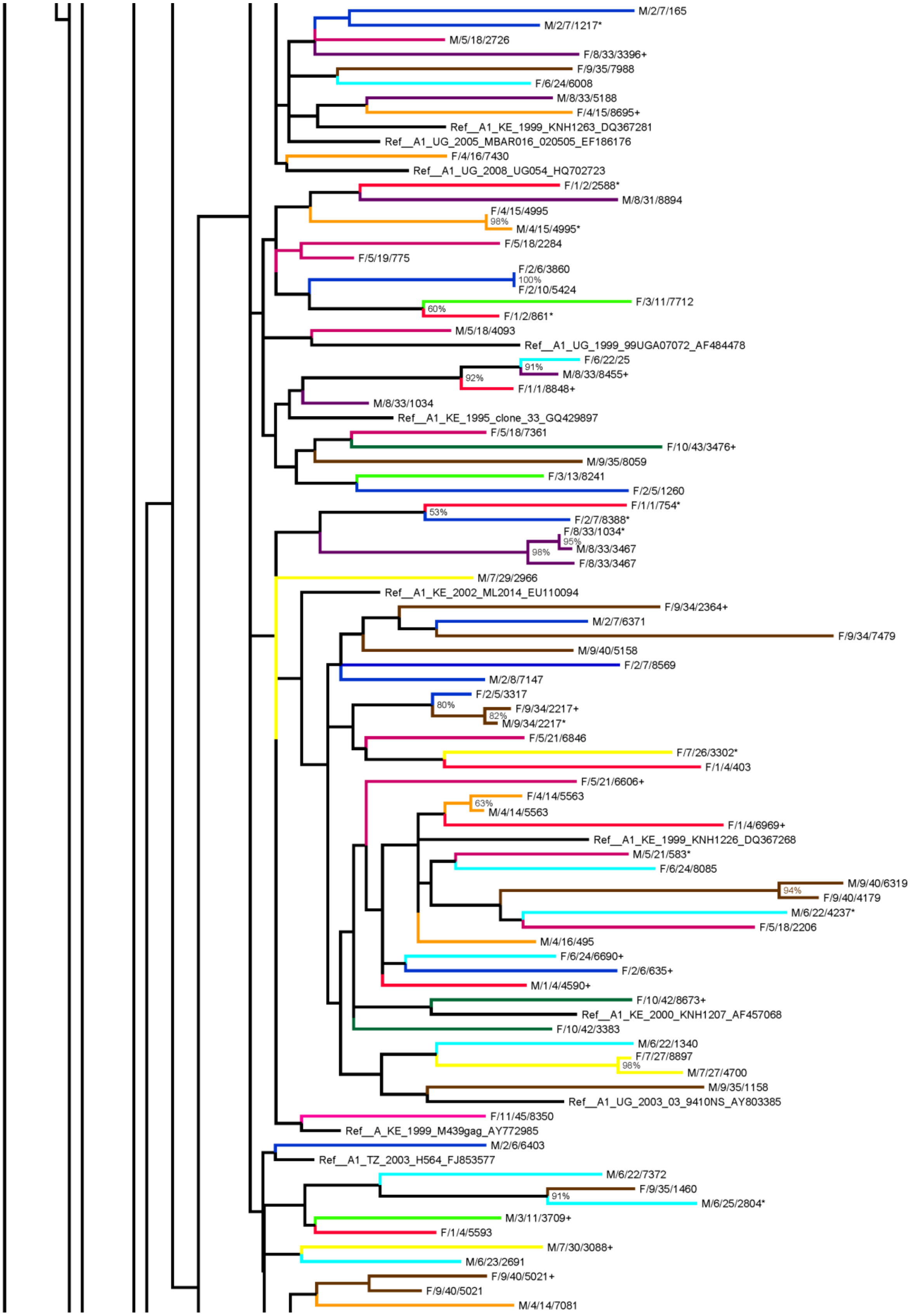
M/2/7/165
M/2/7/1217*
M/5/18/2726
F/8/33/3396+
F/9/35/7988
F/6/24/6008
M/8/33/5188
F/4/15/8695+
Ref__A1_KE_1999_KNH1263_DQ367281
Ref__A1_UG_2005_MBAR016_020505_EF186176
F/4/16/7430
Ref__A1_UG_2008_UG054_HQ702723
F/1/2/2588*
M/8/31/8894
F/4/15/4995
98%
M/4/15/4995*
F/5/18/2284
F/5/19/775
F/2/6/3860
100%
F/2/10/5424
F/3/11/7712
60%
F/1/2/861*
M/5/18/4093
Ref__A1_UG_1999_99UGA07072_AF484478
F/6/22/25
91%
M/8/33/8455+
92%
F/1/1/8848+
M/8/33/1034
Ref__A1_KE_1995_clone_33_GQ429897
F/5/18/7361
F/10/43/3476+
M/9/35/8059
F/3/13/8241
F/2/5/1260
F/1/1/754*
53%
F/2/7/8388*
F/8/33/1034*
95%
98%
M/8/33/3467
F/8/33/3467
M/7/29/2966
Ref__A1_KE_2002_ML2014_EU110094
F/9/34/2364+
M/2/7/6371
F/9/34/7479
M/9/40/5158
F/2/7/8569
M/2/8/7147
F/2/5/3317
80%
F/9/34/2217+
82%
M/9/34/2217*
F/5/21/6846
F/7/26/3302*
F/1/4/403
F/5/21/6606+
F/4/14/5563
63%
M/4/14/5563
F/1/4/6969+
Ref__A1_KE_1999_KNH1226_DQ367268
M/5/21/583*
F/6/24/8085
M/9/40/6319
94%
F/9/40/4179
M/6/22/4237*
F/5/18/2206
M/4/16/495
F/6/24/6690+
F/2/6/635+
M/1/4/4590+
F/10/42/8673+
Ref__A1_KE_2000_KNH1207_AF457068
F/10/42/3383
M/6/22/1340
F/7/27/8897
98%
M/7/27/4700
M/9/35/1158
Ref__A1_UG_2003_03_9410NS_AY803385
F/11/45/8350
Ref__A_KE_1999_M439gag_AY772985
M/2/6/6403
Ref__A1_TZ_2003_H564_FJ853577
M/6/22/7372
F/9/35/1460
91%
M/6/25/2804*
M/3/11/3709+
F/1/4/5593
M/7/30/3088+
M/6/23/2691
F/9/40/5021+
F/9/40/5021
M/4/14/7081

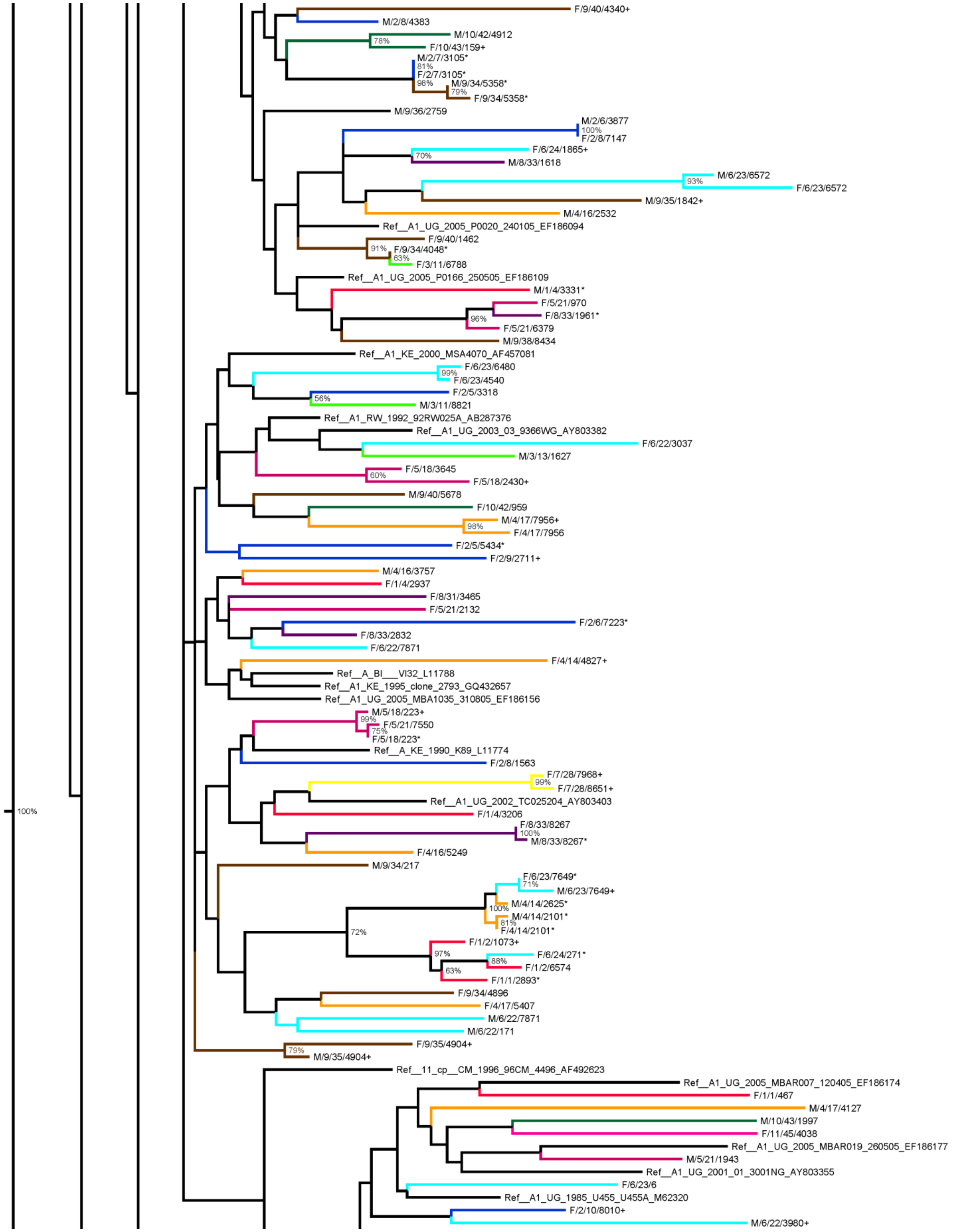

F/9/40/4340+
M/2/8/4383
M/10/42/4912
78%
F/10/43/159+
M/2/7/3105*
81%
F/2/7/3105*
98%
M/9/34/5358*
79%
F/9/34/5358*
M/9/36/2759
M/2/6/3877
100%
F/2/8/7147
F/6/24/1865+
70%
M/8/33/1618
M/6/23/6572
93%
F/6/23/6572
M/9/35/1842+
M/4/16/2532
Ref__A1_UG_2005_P0020_240105_EF186094
F/9/40/1462
91%
F/9/34/4048*
63%
F/3/11/6788
Ref__A1_UG_2005_P0166_250505_EF186109
M/1/4/3331*
F/5/21/970
F/8/33/1961*
96%
F/5/21/6379
M/9/38/8434
Ref__A1_KE_2000_MSA4070_AF457081
F/6/23/6480
99%
F/6/23/4540
F/2/5/3318
56%
M/3/11/8821
Ref__A1_RW_1992_92RW025A_AB287376
Ref__A1_UG_2003_03_9366WG_AY803382
F/6/22/3037
M/3/13/1627
F/5/18/3645
60%
F/5/18/2430+
M/9/40/5678
F/10/42/959
M/4/17/7956+
98%
F/4/17/7956
F/2/5/5434*
F/2/9/2711+
M/4/16/3757
F/1/4/2937
F/8/31/3465
F/5/21/2132
F/2/6/7223*
F/8/33/2832
F/6/22/7871
F/4/14/4827+
Ref__A_BI___VI32_L11788
Ref__A1_KE_1995_clone_2793_GQ432657
Ref__A1_UG_2005_MBA1035_310805_EF186156
M/5/18/223+
99%
F/5/21/7550
75%
F/5/18/223*
Ref__A_KE_1990_K89_L11774
F/2/8/1563
F/7/28/7968+
99%
F/7/28/8651+
Ref__A1_UG_2002_TC025204_AY803403
F/1/4/3206
F/8/33/8267
100%
M/8/33/8267*
F/4/16/5249
100%
M/9/34/217
F/6/23/7649*
71%
M/6/23/7649+
100%
M/4/14/2625*
M/4/14/2101*
81%
F/4/14/2101*
72%
F/1/2/1073+
97%
F/6/24/271*
88%
F/1/2/6574
63%
F/1/1/2893*
F/9/34/4896
F/4/17/5407
M/6/22/7871
M/6/22/171
F/9/35/4904+
79%
M/9/35/4904+
Ref__11_cp__CM_1996_96CM_4496_AF492623
Ref__A1_UG_2005_MBAR007_120405_EF186174
F/1/1/467
M/4/17/4127
M/10/43/1997
F/11/45/4038
Ref__A1_UG_2005_MBAR019_260505_EF186177
M/5/21/1943
Ref__A1_UG_2001_01_3001NG_AY803355
F/6/23/6
Ref__A1_UG_1985_U455_U455A_M62320
F/2/10/8010+
M/6/22/3980+

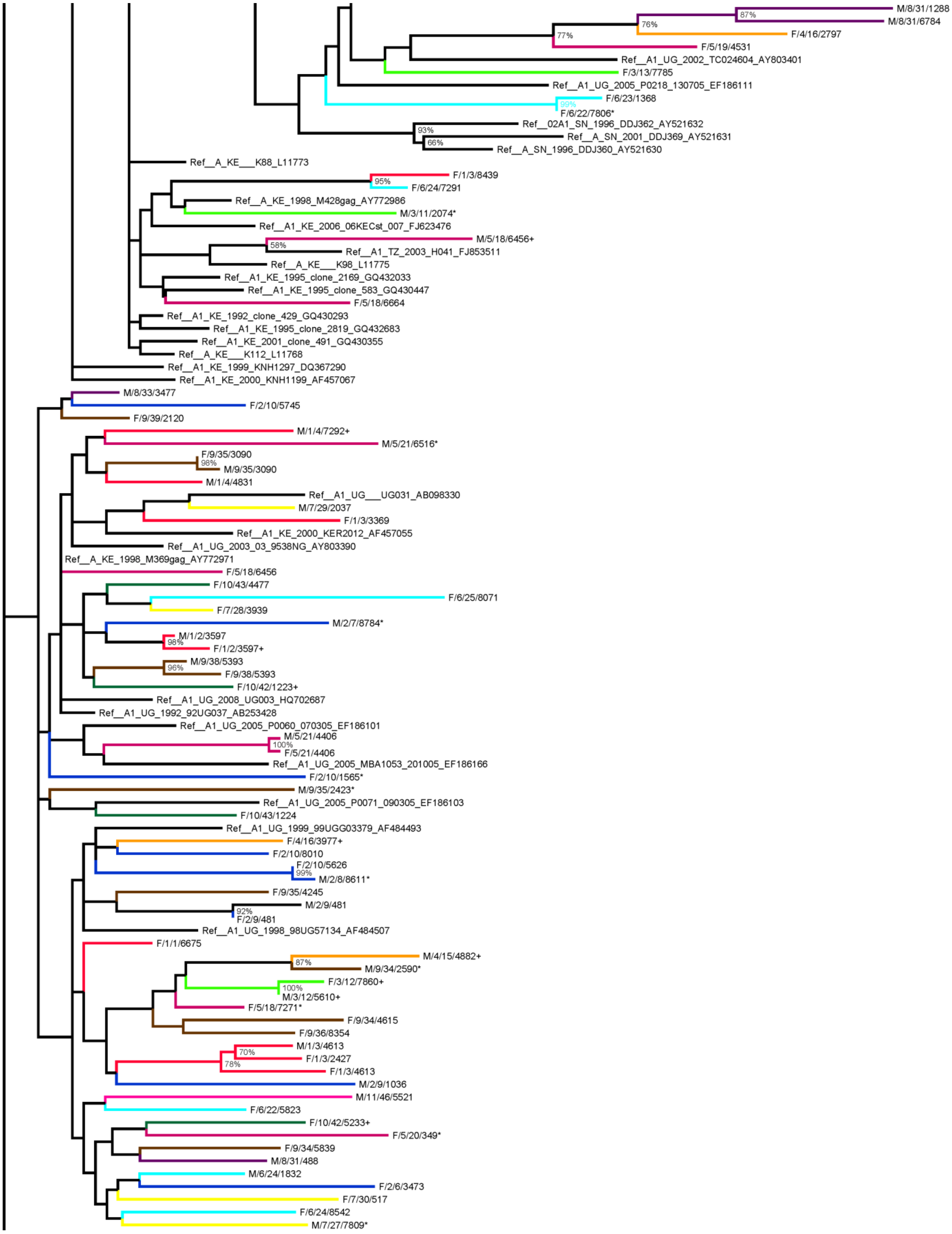
M/8/31/1288
87%
M/8/31/6784
76%
F/4/16/2797
77%
F/5/19/4531
Ref__A1_UG_2002_TC024604_AY803401
F/3/13/7785
Ref__A1_UG_2005_P0218_130705_EF186111
F/6/23/1368
99%
F/6/22/7806*
Ref__02A1_SN_1996_DDJ362_AY521632
93%
Ref__A_SN_2001_DDJ369_AY521631
66%
Ref__A_SN_1996_DDJ360_AY521630
Ref__A_KE___K88_L11773
F/1/3/8439
95%
F/6/24/7291
Ref__A_KE_1998_M428gag_AY772986
M/3/11/2074*
Ref__A1_KE_2006_06KECst_007_FJ623476
M/5/18/6456+
58%
Ref__A1_TZ_2003_H041_FJ853511
Ref__A_KE___K98_L11775
Ref__A1_KE_1995_clone_2169_GQ432033
Ref__A1_KE_1995_clone_583_GQ430447
F/5/18/6664
Ref__A1_KE_1992_clone_429_GQ430293
Ref__A1_KE_1995_clone_2819_GQ432683
Ref__A1_KE_2001_clone_491_GQ430355
Ref__A_KE___K112_L11768
Ref__A1_KE_1999_KNH1297_DQ367290
Ref__A1_KE_2000_KNH1199_AF457067
M/8/33/3477
F/2/10/5745
F/9/39/2120
M/1/4/7292+
M/5/21/6516*
F/9/35/3090
98%
M/9/35/3090
M/1/4/4831
Ref__A1_UG___UG031_AB098330
M/7/29/2037
F/1/3/3369
Ref__A1_KE_2000_KER2012_AF457055
Ref__A1_UG_2003_03_9538NG_AY803390
Ref__A_KE_1998_M369gag_AY772971
F/5/18/6456
F/10/43/4477
F/6/25/8071
F/7/28/3939
M/2/7/8784*
M/1/2/3597
98%
F/1/2/3597+
M/9/38/5393
96%
F/9/38/5393
F/10/42/1223+
Ref__A1_UG_2008_UG003_HQ702687
Ref__A1_UG_1992_92UG037_AB253428
Ref__A1_UG_2005_P0060_070305_EF186101
M/5/21/4406
100%
F/5/21/4406
Ref__A1_UG_2005_MBA1053_201005_EF186166
F/2/10/1565*
M/9/35/2423*
Ref__A1_UG_2005_P0071_090305_EF186103
F/10/43/1224
Ref__A1_UG_1999_99UGG03379_AF484493
F/4/16/3977+
F/2/10/8010
F/2/10/5626
99%
M/2/8/8611*
F/9/35/4245
M/2/9/481
92%
F/2/9/481
Ref__A1_UG_1998_98UG57134_AF484507
F/1/1/6675
M/4/15/4882+
87%
M/9/34/2590*
F/3/12/7860+
100%
M/3/12/5610+
F/5/18/7271*
F/9/34/4615
F/9/36/8354
M/1/3/4613
70%
F/1/3/2427
78%
F/1/3/4613
M/2/9/1036
M/11/46/5521
F/6/22/5823
F/10/42/5233+
F/5/20/349*
F/9/34/5839
M/8/31/488
M/6/24/1832
F/2/6/3473
F/7/30/517
F/6/24/8542
M/7/27/7809*

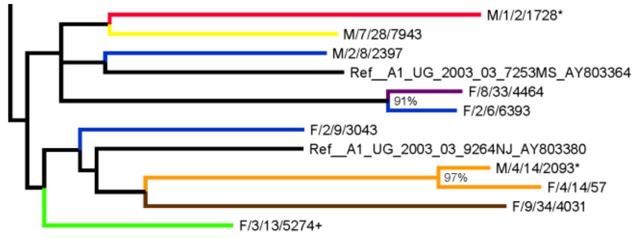
M/1/2/1728*
M/7/28/7943
M/2/8/2397
Ref__A1_UG_2003_03_7253MS_AY803364
F/8/33/4464
91%
F/2/6/6393
F/2/9/3043
Ref__A1_UG_2003_03_9264NJ_AY803380
M/4/14/2093*
97%
F/4/14/57
F/9/34/4031
F/3/13/5274+
