# Supplementary material for: The Role of Viral Introductions in Sustaining Community-Based HIV Epidemics in Rural Uganda: Evidence from Spatial Clustering, Phylogenetics, and Egocentric Transmission Models
Source: PLoS Med. 2014 Mar 4;11(3):e1001610. doi: 10.1371/journal.pmed.1001610 (PMC3942316; doi:10.1371/journal.pmed.1001610)
Supplement: Figure S7 — Maximum likelihood tree (rectangular) of gag HIV-1 subtype D sequences. Taxa are labeled using participant gender/geographic region/community/household. Reference sequences (n = 57) are in black, and only bootstrap values ≥50% are shown. Color corresponds to the geographic region. (PDF) [file pmed.1001610.s007.pdf]

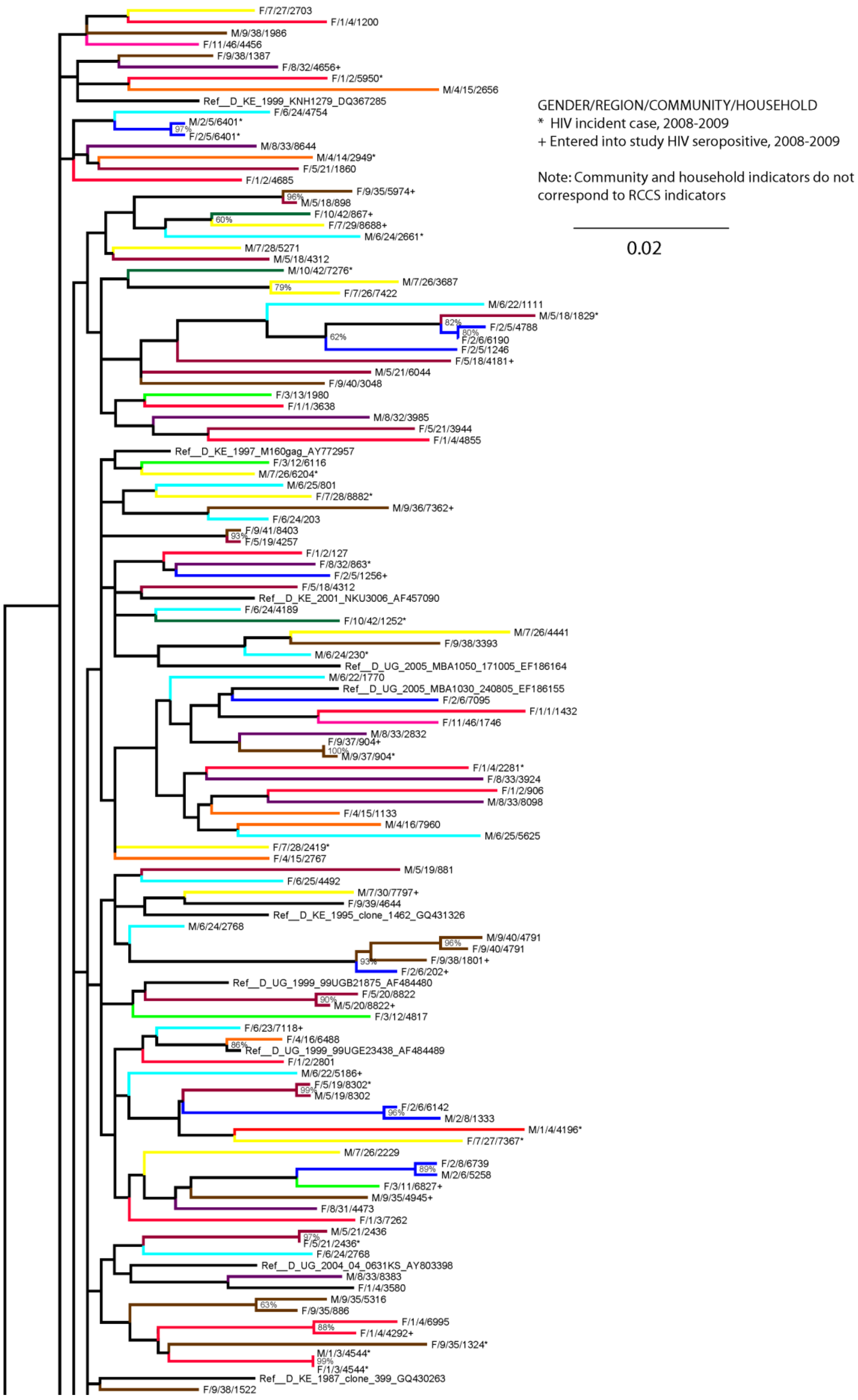

GENDER/REGION/COMMUNITY/HOUSEHOLD
* HIV incident case, 2008-2009
+ Entered into study HIV seropositive, 2008-2009
Note: Community and household indicators do not correspond to RCCS indicators
0.02
F/7/27/2703
F/1/4/1200
M/9/38/1986
F/11/46/4456
F/9/38/1387
F/8/32/4656+
F/1/2/5950*
M/4/15/2656
Ref__D_KE_1999_KNH1279_DQ367285
F/6/24/4754
M/2/5/6401*
97%
F/2/5/6401*
M/8/33/8644
M/4/14/2949*
F/5/21/1860
F/1/2/4685
F/9/35/5974+
96%
M/5/18/898
F/10/42/867+
60%
F/7/29/8688+
M/6/24/2661*
M/7/28/5271
M/5/18/4312
M/10/42/7276*
M/7/26/3687
79%
F/7/26/7422
M/6/22/1111
M/5/18/1829*
82%
F/2/5/4788
80%
F/2/6/6190
62%
F/2/5/1246
F/5/18/4181+
M/5/21/6044
F/9/40/3048
F/3/13/1980
F/1/1/3638
M/8/32/3985
F/5/21/3944
F/1/4/4855
Ref__D_KE_1997_M160gag_AY772957
F/3/12/6116
M/7/26/6204*
M/6/25/801
F/7/28/8882*
M/9/36/7362+
F/6/24/203
F/9/41/8403
93%
F/5/19/4257
F/1/2/127
F/8/32/863*
F/2/5/1256+
F/5/18/4312
Ref__D_KE_2001_NKU3006_AF457090
F/6/24/4189
F/10/42/1252*
M/7/26/4441
F/9/38/3393
M/6/24/230*
Ref__D_UG_2005_MBA1050_171005_EF186164
M/6/22/1770
Ref__D_UG_2005_MBA1030_240805_EF186155
F/2/6/7095
F/1/1/1432
F/11/46/1746
M/8/33/2832
F/9/37/904+
100%
M/9/37/904*
F/1/4/2281*
F/8/33/3924
F/1/2/906
M/8/33/8098
F/4/15/1133
M/4/16/7960
M/6/25/5625
F/7/28/2419*
F/4/15/2767
M/5/19/881
F/6/25/4492
M/7/30/7797+
F/9/39/4644
Ref__D_KE_1995_clone_1462_GQ431326
M/6/24/2768
M/9/40/4791
96%
F/9/40/4791
F/9/38/1801+
93%
F/2/6/202+
Ref__D_UG_1999_99UGB21875_AF484480
F/5/20/8822
90%
M/5/20/8822+
F/3/12/4817
F/6/23/7118+
F/4/16/6488
86%
Ref__D_UG_1999_99UGE23438_AF484489
F/1/2/2801
M/6/22/5186+
F/5/19/8302*
99%
M/5/19/8302
F/2/6/6142
96%
M/2/8/1333
M/1/4/4196*
F/7/27/7367*
M/7/26/2229
F/2/8/6739
89%
M/2/6/5258
F/3/11/6827+
M/9/35/4945+
F/8/31/4473
F/1/3/7262
M/5/21/2436
97%
F/5/21/2436*
F/6/24/2768
Ref__D_UG_2004_04_0631KS_AY803398
M/8/33/8383
F/1/4/3580
M/9/35/5316
63%
F/9/35/886
F/1/4/6995
88%
F/1/4/4292+
F/9/35/1324*
M/1/3/4544*
99%
F/1/3/4544*
Ref__D_KE_1987_clone_399_GQ430263
F/9/38/1522

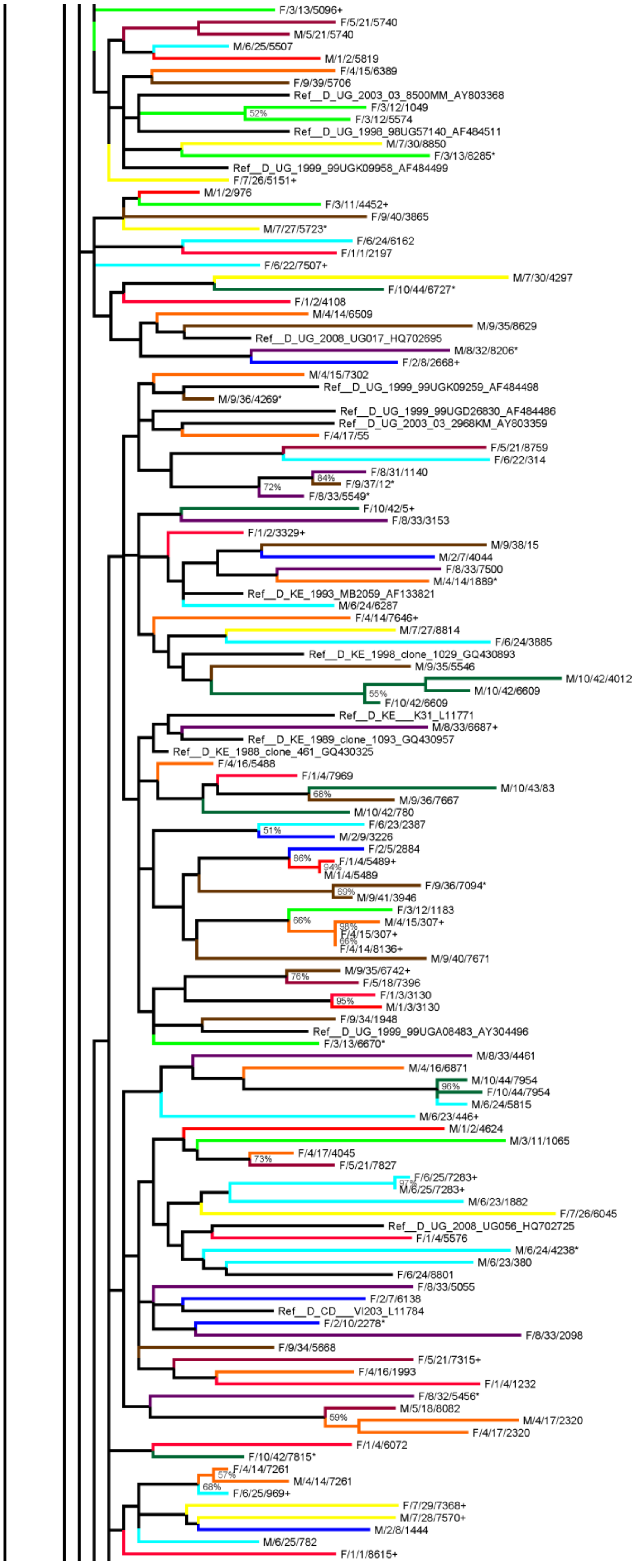
F/3/13/5096+
F/5/21/5740
M/5/21/5740
M/6/25/5507
M/1/2/5819
F/4/15/6389
F/9/39/5706
Ref__D_UG_2003_03_8500MM_AY803368
F/3/12/1049
F/3/12/5574
Ref__D_UG_1998_98UG57140_AF484511
M/7/30/8850
F/3/13/8285*
Ref__D_UG_1999_99UGK09958_AF484499
F/7/26/5151+
M/1/2/976
F/3/11/4452+
F/9/40/3865
M/7/27/5723*
F/6/24/6162
F/1/1/2197
F/6/22/7507+
M/7/30/4297
F/10/44/6727*
F/1/2/4108
M/4/14/6509
M/9/35/8629
Ref__D_UG_2008_UG017_HQ702695
M/8/32/8206*
F/2/8/2668+
M/4/15/7302
Ref__D_UG_1999_99UGK09259_AF484498
M/9/36/4269*
Ref__D_UG_1999_99UGD26830_AF484486
Ref__D_UG_2003_03_2968KM_AY803359
F/4/17/55
F/5/21/8759
F/6/22/314
F/8/31/1140
F/9/37/12*
F/8/33/5549*
F/10/42/5+
F/8/33/3153
F/1/2/3329+
M/9/38/15
M/2/7/4044
F/8/33/7500
M/4/14/1889*
Ref__D_KE_1993_MB2059_AF133821
M/6/24/6287
F/4/14/7646+
M/7/27/8814
F/6/24/3885
Ref__D_KE_1998_clone_1029_GQ430893
M/9/35/5546
M/10/42/4012
M/10/42/6609
F/10/42/6609
Ref__D_KE___K31_L11771
M/8/33/6687+
Ref__D_KE_1989_clone_1093_GQ430957
Ref__D_KE_1988_clone_461_GQ430325
F/4/16/5488
F/1/4/7969
M/10/43/83
M/9/36/7667
M/10/42/780
F/6/23/2387
M/2/9/3226
F/2/5/2884
F/1/4/5489+
M/1/4/5489
F/9/36/7094*
M/9/41/3946
F/3/12/1183
M/4/15/307+
F/4/15/307+
F/4/14/8136+
M/9/40/7671
M/9/35/6742+
F/5/18/7396
F/1/3/3130
M/1/3/3130
F/9/34/1948
Ref__D_UG_1999_99UGA08483_AY304496
F/3/13/6670*
M/8/33/4461
M/4/16/6871
M/10/44/7954
F/10/44/7954
M/6/24/5815
M/6/23/446+
M/1/2/4624
M/3/11/1065
F/4/17/4045
F/5/21/7827
F/6/25/7283+
M/6/25/7283+
M/6/23/1882
F/7/26/6045
Ref__D_UG_2008_UG056_HQ702725
F/1/4/5576
M/6/24/4238*
M/6/23/380
F/6/24/8801
F/8/33/5055
F/2/7/6138
Ref__D_CD___VI203_L11784
F/2/10/2278*
F/8/33/2098
F/9/34/5668
F/5/21/7315+
F/4/16/1993
F/1/4/1232
F/8/32/5456*
M/5/18/8082
M/4/17/2320
F/4/17/2320
F/1/4/6072
F/10/42/7815*
F/4/14/7261
M/4/14/7261
F/6/25/969+
F/7/29/7368+
M/7/28/7570+
M/2/8/1444
M/6/25/782
F/1/1/8615+
52%
84%
72%
55%
68%
51%
86%
94%
69%
66%
98%
66%
76%
95%
96%
73%
97%
59%
57%
68%

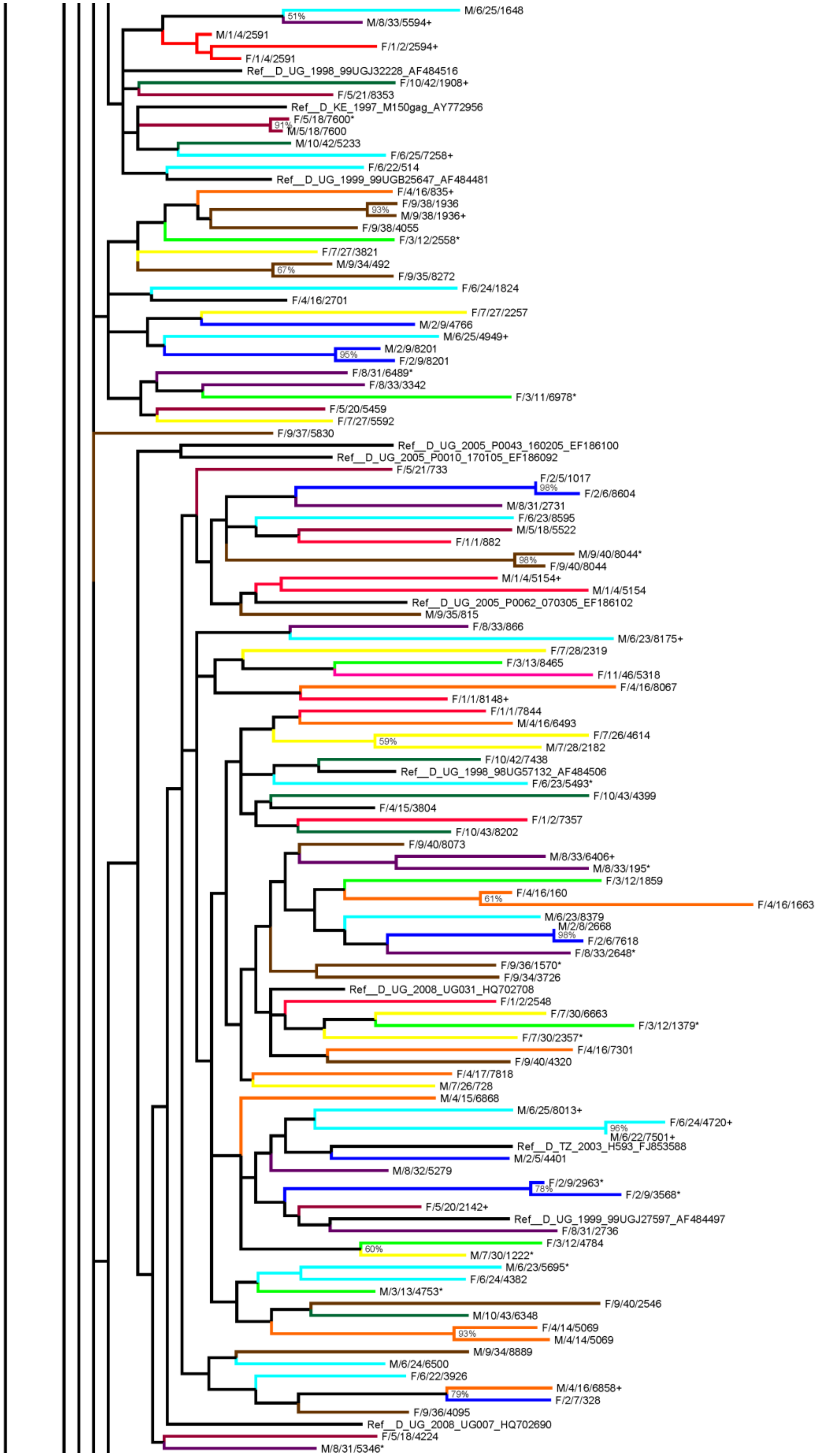

M/6/25/1648
51%
M/8/33/5594+
M/1/4/2591
F/1/2/2594+
F/1/4/2591
Ref__D_UG_1998_99UGJ32228_AF484516
F/10/42/1908+
F/5/21/8353
Ref__D_KE_1997_M150gag_AY772956
F/5/18/7600*
91%
M/5/18/7600
M/10/42/5233
F/6/25/7258+
F/6/22/514
Ref__D_UG_1999_99UGB25647_AF484481
F/4/16/835+
F/9/38/1936
93%
M/9/38/1936+
F/9/38/4055
F/3/12/2558*
F/7/27/3821
M/9/34/492
67%
F/9/35/8272
F/6/24/1824
F/4/16/2701
F/7/27/2257
M/2/9/4766
M/6/25/4949+
M/2/9/8201
95%
F/2/9/8201
F/8/31/6489*
F/8/33/3342
F/3/11/6978*
F/5/20/5459
F/7/27/5592
F/9/37/5830
Ref__D_UG_2005_P0043_160205_EF186100
Ref__D_UG_2005_P0010_170105_EF186092
F/5/21/733
F/2/5/1017
98%
F/2/6/8604
M/8/31/2731
F/6/23/8595
M/5/18/5522
F/1/1/882
M/9/40/8044*
98%
F/9/40/8044
M/1/4/5154+
M/1/4/5154
Ref__D_UG_2005_P0062_070305_EF186102
M/9/35/815
F/8/33/866
M/6/23/8175+
F/7/28/2319
F/3/13/8465
F/11/46/5318
F/4/16/8067
F/1/1/8148+
F/1/1/7844
M/4/16/6493
F/7/26/4614
59%
M/7/28/2182
F/10/42/7438
Ref__D_UG_1998_98UG57132_AF484506
F/6/23/5493*
F/10/43/4399
F/4/15/3804
F/1/2/7357
F/10/43/8202
F/9/40/8073
M/8/33/6406+
M/8/33/195*
F/3/12/1859
F/4/16/160
61%
F/4/16/1663
M/6/23/8379
M/2/8/2668
98%
F/2/6/7618
F/8/33/2648*
F/9/36/1570*
F/9/34/3726
Ref__D_UG_2008_UG031_HQ702708
F/1/2/2548
F/7/30/6663
F/3/12/1379*
F/7/30/2357*
F/4/16/7301
F/9/40/4320
F/4/17/7818
M/7/26/728
M/4/15/6868
M/6/25/8013+
F/6/24/4720+
96%
M/6/22/7501+
Ref__D_TZ_2003_H593_FJ853588
M/2/5/4401
M/8/32/5279
F/2/9/2963*
78%
F/2/9/3568*
F/5/20/2142+
Ref__D_UG_1999_99UGJ27597_AF484497
F/8/31/2736
F/3/12/4784
60%
M/7/30/1222*
M/6/23/5695*
F/6/24/4382
M/3/13/4753*
F/9/40/2546
M/10/43/6348
F/4/14/5069
93%
M/4/14/5069
M/9/34/8889
M/6/24/6500
F/6/22/3926
M/4/16/6858+
79%
F/2/7/328
F/9/36/4095
Ref__D_UG_2008_UG007_HQ702690
F/5/18/4224
M/8/31/5346*

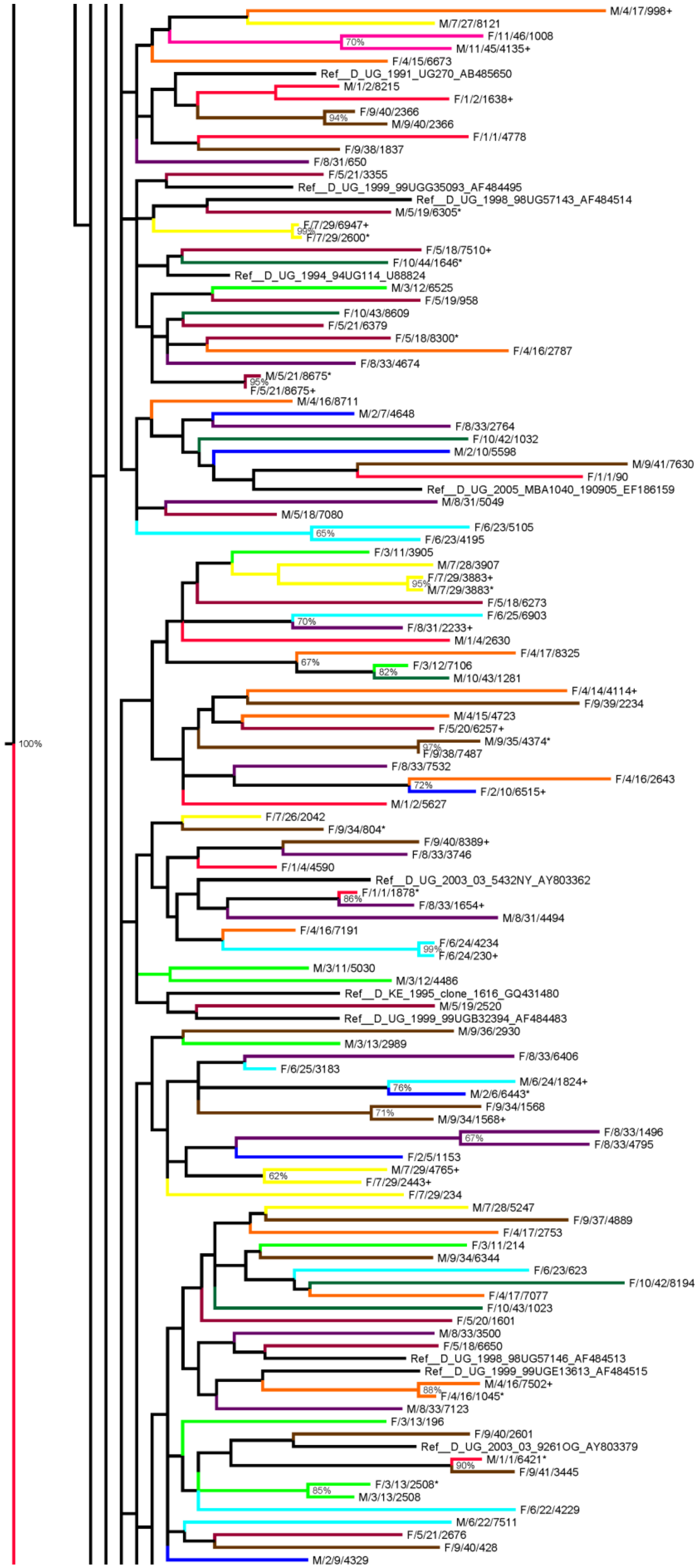

M/4/17/998+
M/7/27/8121
F/11/46/1008
70%
M/11/45/4135+
F/4/15/6673
Ref__D_UG_1991_UG270_AB485650
M/1/2/8215
F/1/2/1638+
F/9/40/2366
94%
M/9/40/2366
F/1/1/4778
F/9/38/1837
F/8/31/650
F/5/21/3355
Ref__D_UG_1999_99UGG35093_AF484495
Ref__D_UG_1998_98UG57143_AF484514
M/5/19/6305*
F/7/29/6947+
95%
F/7/29/2600*
F/5/18/7510+
F/10/44/1646*
Ref__D_UG_1994_94UG114_U88824
M/3/12/6525
F/5/19/958
F/10/43/8609
F/5/21/6379
F/5/18/8300*
F/4/16/2787
F/8/33/4674
M/5/21/8675*
95%
F/5/21/8675+
M/4/16/8711
M/2/7/4648
F/8/33/2764
F/10/42/1032
M/2/10/5598
M/9/41/7630
F/1/1/90
Ref__D_UG_2005_MBA1040_190905_EF186159
M/8/31/5049
M/5/18/7080
F/6/23/5105
65%
F/6/23/4195
F/3/11/3905
M/7/28/3907
F/7/29/3883+
95%
M/7/29/3883*
F/5/18/6273
70%
F/6/25/6903
F/8/31/2233+
M/1/4/2630
F/4/17/8325
67%
F/3/12/7106
82%
M/10/43/1281
F/4/14/4114+
F/9/39/2234
M/4/15/4723
F/5/20/6257+
M/9/35/4374*
97%
F/9/38/7487
F/8/33/7532
F/4/16/2643
72%
F/2/10/6515+
M/1/2/5627
F/7/26/2042
F/9/34/804*
F/9/40/8389+
F/8/33/3746
F/1/4/4590
Ref__D_UG_2003_03_5432NY_AY803362
F/1/1/1878*
86%
F/8/33/1654+
M/8/31/4494
F/4/16/7191
F/6/24/4234
99%
F/6/24/230+
M/3/11/5030
M/3/12/4486
Ref__D_KE_1995_clone_1616_GQ431480
M/5/19/2520
Ref__D_UG_1999_99UGB32394_AF484483
M/9/36/2930
M/3/13/2989
F/8/33/6406
F/6/25/3183
M/6/24/1824+
76%
M/2/6/6443*
F/9/34/1568
71%
M/9/34/1568+
F/8/33/1496
67%
F/8/33/4795
F/2/5/1153
M/7/29/4765+
62%
F/7/29/2443+
F/7/29/234
M/7/28/5247
F/9/37/4889
F/4/17/2753
F/3/11/214
M/9/34/6344
F/6/23/623
F/10/42/8194
F/4/17/7077
F/10/43/1023
F/5/20/1601
M/8/33/3500
F/5/18/6650
Ref__D_UG_1998_98UG57146_AF484513
Ref__D_UG_1999_99UGE13613_AF484515
M/4/16/7502+
88%
F/4/16/1045*
M/8/33/7123
F/3/13/196
F/9/40/2601
Ref__D_UG_2003_03_9261OG_AY803379
M/1/1/6421*
90%
F/9/41/3445
F/3/13/2508*
85%
M/3/13/2508
F/6/22/4229
M/6/22/7511
F/5/21/2676
F/9/40/428
M/2/9/4329
100%

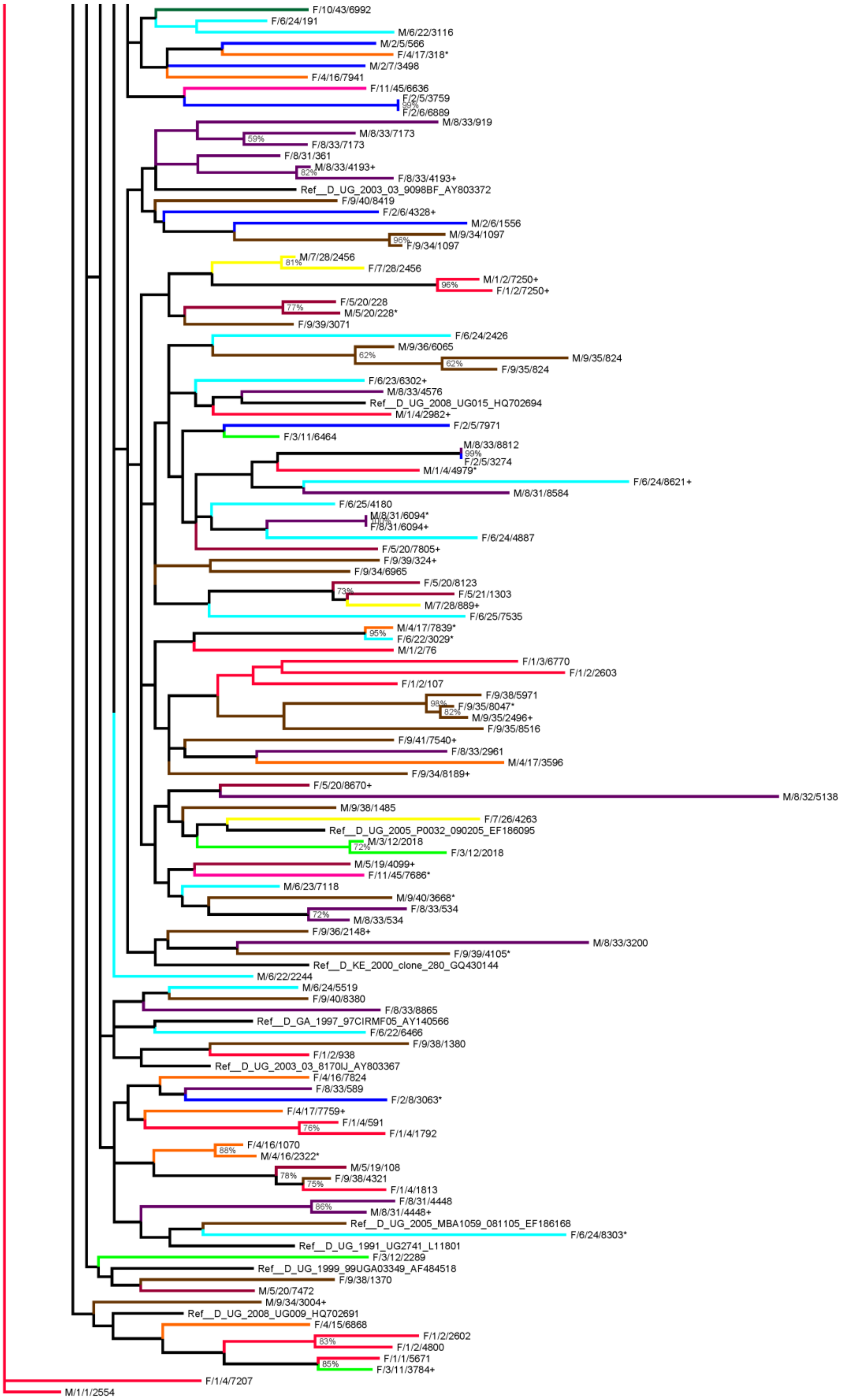
F/10/43/6992
F/6/24/191
M/6/22/3116
M/2/5/566
F/4/17/318*
M/2/7/3498
F/4/16/7941
F/11/45/6636
F/2/5/3759
F/2/6/6889
M/8/33/919
M/8/33/7173
F/8/33/7173
F/8/31/361
M/8/33/4193+
F/8/33/4193+
Ref__D_UG_2003_03_9098BF_AY803372
F/9/40/8419
F/2/6/4328+
M/2/6/1556
M/9/34/1097
F/9/34/1097
M/7/28/2456
F/7/28/2456
M/1/2/7250+
F/1/2/7250+
F/5/20/228
M/5/20/228*
F/9/39/3071
F/6/24/2426
M/9/36/6065
M/9/35/824
F/9/35/824
F/6/23/6302+
M/8/33/4576
Ref__D_UG_2008_UG015_HQ702694
M/1/4/2982+
F/2/5/7971
F/3/11/6464
M/8/33/8812
F/2/5/3274
M/1/4/4979*
F/6/24/8621+
M/8/31/8584
F/6/25/4180
M/8/31/6094*
F/8/31/6094+
F/6/24/4887
F/5/20/7805+
F/9/39/324+
F/9/34/6965
F/5/20/8123
F/5/21/1303
M/7/28/889+
F/6/25/7535
M/4/17/7839*
F/6/22/3029*
M/1/2/76
F/1/3/6770
F/1/2/2603
F/1/2/107
F/9/38/5971
F/9/35/8047*
M/9/35/2496+
F/9/35/8516
F/9/41/7540+
F/8/33/2961
M/4/17/3596
F/9/34/8189+
F/5/20/8670+
M/8/32/5138
M/9/38/1485
F/7/26/4263
Ref__D_UG_2005_P0032_090205_EF186095
M/3/12/2018
F/3/12/2018
M/5/19/4099+
F/11/45/7686*
M/6/23/7118
M/9/40/3668*
F/8/33/534
M/8/33/534
F/9/36/2148+
M/8/33/3200
F/9/39/4105*
Ref__D_KE_2000_clone_280_GQ430144
M/6/22/2244
M/6/24/5519
F/9/40/8380
F/8/33/8865
Ref__D_GA_1997_97CIRMF05_AY140566
F/6/22/6466
F/9/38/1380
F/1/2/938
Ref__D_UG_2003_03_8170IJ_AY803367
F/4/16/7824
F/8/33/589
F/2/8/3063*
F/4/17/7759+
F/1/4/591
F/1/4/1792
F/4/16/1070
M/4/16/2322*
M/5/19/108
F/9/38/4321
F/1/4/1813
F/8/31/4448
M/8/31/4448+
Ref__D_UG_2005_MBA1059_081105_EF186168
F/6/24/8303*
Ref__D_UG_1991_UG2741_L11801
F/3/12/2289
Ref__D_UG_1999_99UGA03349_AF484518
F/9/38/1370
M/5/20/7472
M/9/34/3004+
Ref__D_UG_2008_UG009_HQ702691
F/4/15/6868
F/1/2/2602
F/1/2/4800
F/1/1/5671
F/3/11/3784+
F/1/4/7207
M/1/1/2554
