# Supplementary material for: The Role of Viral Introductions in Sustaining Community-Based HIV Epidemics in Rural Uganda: Evidence from Spatial Clustering, Phylogenetics, and Egocentric Transmission Models
Source: PLoS Med. 2014 Mar 4;11(3):e1001610. doi: 10.1371/journal.pmed.1001610 (PMC3942316; doi:10.1371/journal.pmed.1001610)
Supplement: Figure S10 — Maximum likelihood tree (rectangular) of env HIV-1 subtype A sequences. Taxa are labeled using participant gender/geographic region/community/household. Reference sequences (n = 107) are in black, and only bootstrap values ≥50% are shown. Color corresponds to the geographic region. (PDF) [file pmed.1001610.s010.pdf]

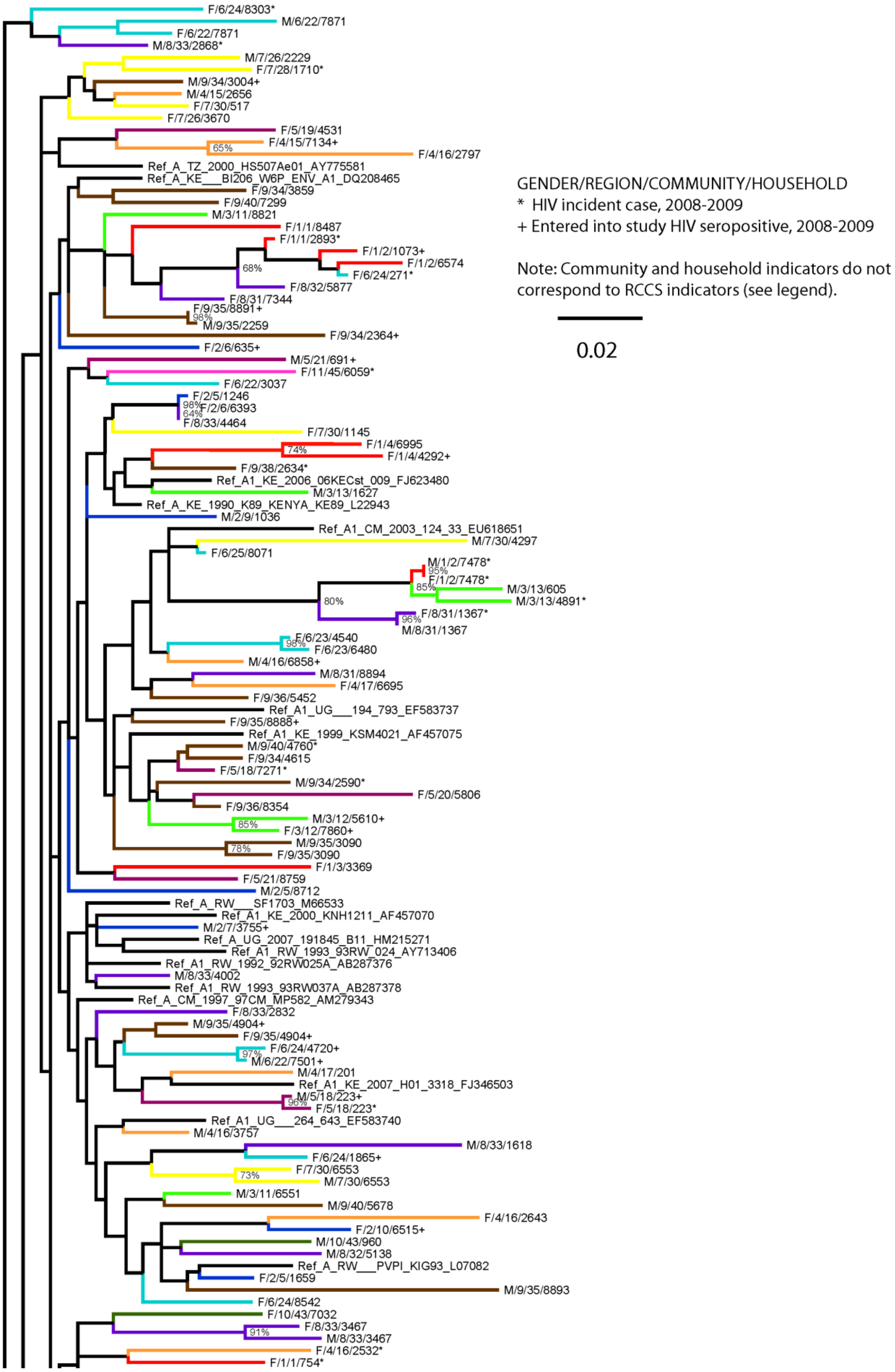
F/6/24/8303*
M/6/22/7871
F/6/22/7871
M/8/33/2868*
M/7/26/2229
F/7/28/1710*
M/9/34/3004+
M/4/15/2656
F/7/30/517
F/7/26/3670
F/5/19/4531
F/4/15/7134+
65%
F/4/16/2797
Ref_A_TZ_2000_HS507Ae01_AY775581
Ref_A_KE___BI206_W6P_ENV_A1_DQ208465
F/9/34/3859
F/9/40/7299
M/3/11/8821
F/1/1/8487
F/1/1/2893*
F/1/2/1073+
68%
F/1/2/6574
F/6/24/271*
F/8/32/5877
F/8/31/7344
F/9/35/8891+
98%
M/9/35/2259
F/9/34/2364+
F/2/6/635+
M/5/21/691+
F/11/45/6059*
F/6/22/3037
F/2/5/1246
98%
F/2/6/6393
64%
F/8/33/4464
F/7/30/1145
F/1/4/6995
74%
F/1/4/4292+
F/9/38/2634*
Ref_A1_KE_2006_06KECst_009_FJ623480
M/3/13/1627
Ref_A_KE_1990_K89_KENYA_KE89_L22943
M/2/9/1036
Ref_A1_CM_2003_124_33_EU618651
M/7/30/4297
F/6/25/8071
M/1/2/7478*
95%
F/1/2/7478*
85%
M/3/13/605
M/3/13/4891*
80%
F/8/31/1367*
96%
M/8/31/1367
F/6/23/4540
98%
F/6/23/6480
M/4/16/6858+
M/8/31/8894
F/4/17/6695
F/9/36/5452
Ref_A1_UG___194_793_EF583737
F/9/35/8888+
Ref_A1_KE_1999_KSM4021_AF457075
M/9/40/4760*
F/9/34/4615
F/5/18/7271*
M/9/34/2590*
F/5/20/5806
F/9/36/8354
M/3/12/5610+
85%
F/3/12/7860+
M/9/35/3090
78%
F/9/35/3090
F/1/3/3369
F/5/21/8759
M/2/5/8712
Ref_A_RW___SF1703_M66533
Ref_A1_KE_2000_KNH1211_AF457070
M/2/7/3755+
Ref_A_UG_2007_191845_B11_HM215271
Ref_A1_RW_1993_93RW_024_AY713406
Ref_A1_RW_1992_92RW025A_AB287376
M/8/33/4002
Ref_A1_RW_1993_93RW037A_AB287378
Ref_A_CM_1997_97CM_MP582_AM279343
F/8/33/2832
M/9/35/4904+
F/9/35/4904+
F/6/24/4720+
97%
M/6/22/7501+
M/4/17/201
Ref_A1_KE_2007_H01_3318_FJ346503
M/5/18/223+
96%
F/5/18/223*
Ref_A1_UG___264_643_EF583740
M/4/16/3757
M/8/33/1618
F/6/24/1865+
F/7/30/6553
73%
M/7/30/6553
M/3/11/6551
M/9/40/5678
F/4/16/2643
F/2/10/6515+
M/10/43/960
M/8/32/5138
Ref_A_RW___PVPI_KIG93_L07082
F/2/5/1659
M/9/35/8893
F/6/24/8542
F/10/43/7032
F/8/33/3467
91%
M/8/33/3467
F/4/16/2532*
F/1/1/754*
GENDER/REGION/COMMUNITY/HOUSEHOLD
* HIV incident case, 2008-2009
+ Entered into study HIV seropositive, 2008-2009
Note: Community and household indicators do not correspond to RCCS indicators (see legend).
0.02

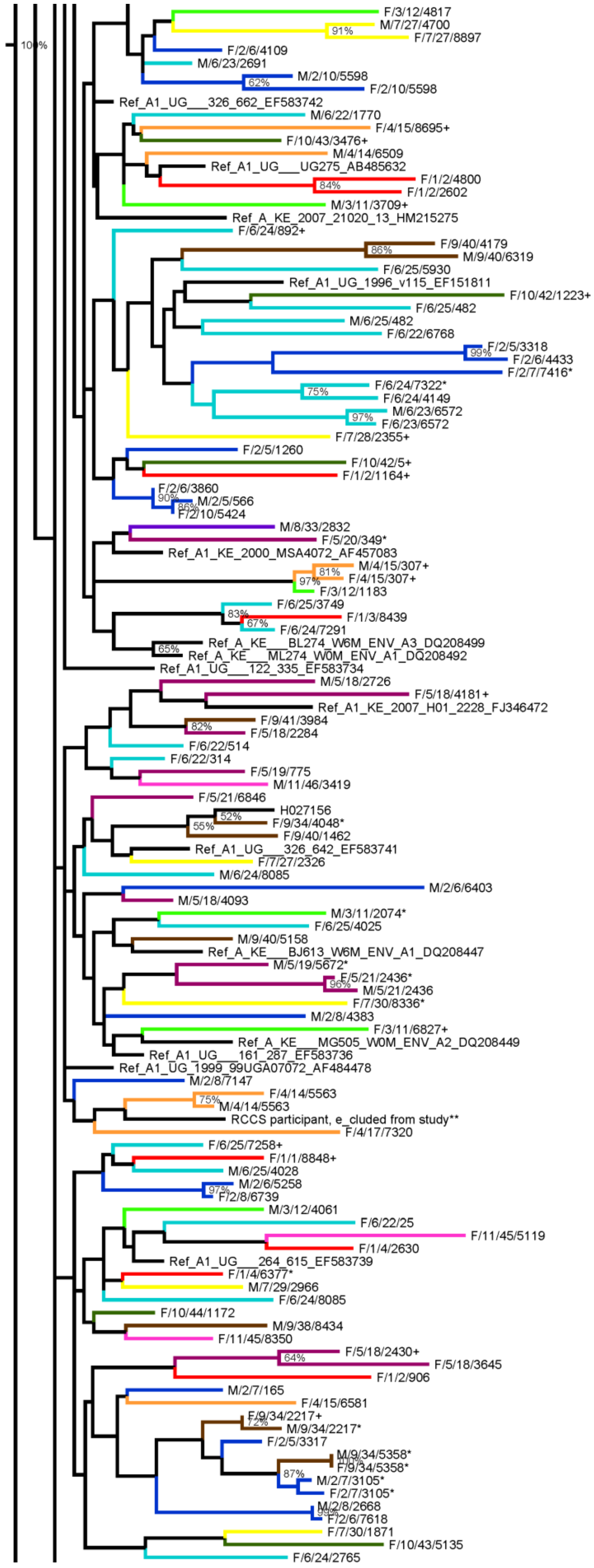

100%
F/3/12/4817
M/7/27/4700
91%
F/7/27/8897
F/2/6/4109
M/6/23/2691
M/2/10/5598
62%
F/2/10/5598
Ref_A1_UG___326_662_EF583742
M/6/22/1770
F/4/15/8695+
F/10/43/3476+
M/4/14/6509
Ref_A1_UG___UG275_AB485632
F/1/2/4800
84%
F/1/2/2602
M/3/11/3709+
Ref_A_KE_2007_21020_13_HM215275
F/6/24/892+
F/9/40/4179
86%
M/9/40/6319
F/6/25/5930
Ref_A1_UG_1996_v115_EF151811
F/10/42/1223+
F/6/25/482
M/6/25/482
F/6/22/6768
F/2/5/3318
99%
F/2/6/4433
F/2/7/7416*
F/6/24/7322*
75%
F/6/24/4149
M/6/23/6572
97%
F/6/23/6572
F/7/28/2355+
F/2/5/1260
F/10/42/5+
F/1/2/1164+
F/2/6/3860
90%
M/2/5/566
86%
F/2/10/5424
M/8/33/2832
F/5/20/349*
Ref_A1_KE_2000_MSA4072_AF457083
M/4/15/307+
81%
F/4/15/307+
97%
F/3/12/1183
F/6/25/3749
83%
F/1/3/8439
67%
F/6/24/7291
Ref_A_KE___BL274_W6M_ENV_A3_DQ208499
65%
Ref_A_KE___ML274_W0M_ENV_A1_DQ208492
Ref_A1_UG___122_335_EF583734
M/5/18/2726
F/5/18/4181+
Ref_A1_KE_2007_H01_2228_FJ346472
F/9/41/3984
82%
F/5/18/2284
F/6/22/514
F/6/22/314
F/5/19/775
M/11/46/3419
F/5/21/6846
H027156
52%
F/9/34/4048*
55%
F/9/40/1462
Ref_A1_UG___326_642_EF583741
F/7/27/2326
M/6/24/8085
M/2/6/6403
M/5/18/4093
M/3/11/2074*
F/6/25/4025
M/9/40/5158
Ref_A_KE___BJ613_W6M_ENV_A1_DQ208447
M/5/19/5672*
F/5/21/2436*
96%
M/5/21/2436
F/7/30/8336*
M/2/8/4383
F/3/11/6827+
Ref_A_KE___MG505_W0M_ENV_A2_DQ208449
Ref_A1_UG___161_287_EF583736
Ref_A1_UG_1999_99UGA07072_AF484478
M/2/8/7147
F/4/14/5563
75%
M/4/14/5563
RCCS participant, e_cluded from study**
F/4/17/7320
F/6/25/7258+
F/1/1/8848+
M/6/25/4028
M/2/6/5258
97%
F/2/8/6739
M/3/12/4061
F/6/22/25
F/11/45/5119
F/1/4/2630
Ref_A1_UG___264_615_EF583739
F/1/4/6377*
M/7/29/2966
F/6/24/8085
F/10/44/1172
M/9/38/8434
F/11/45/8350
F/5/18/2430+
64%
F/5/18/3645
F/1/2/906
M/2/7/165
F/4/15/6581
F/9/34/2217+
72%
M/9/34/2217*
F/2/5/3317
M/9/34/5358*
F/9/34/5358*
87%
M/2/7/3105*
F/2/7/3105*
M/2/8/2668
F/2/6/7618
F/7/30/1871
F/10/43/5135
F/6/24/2765

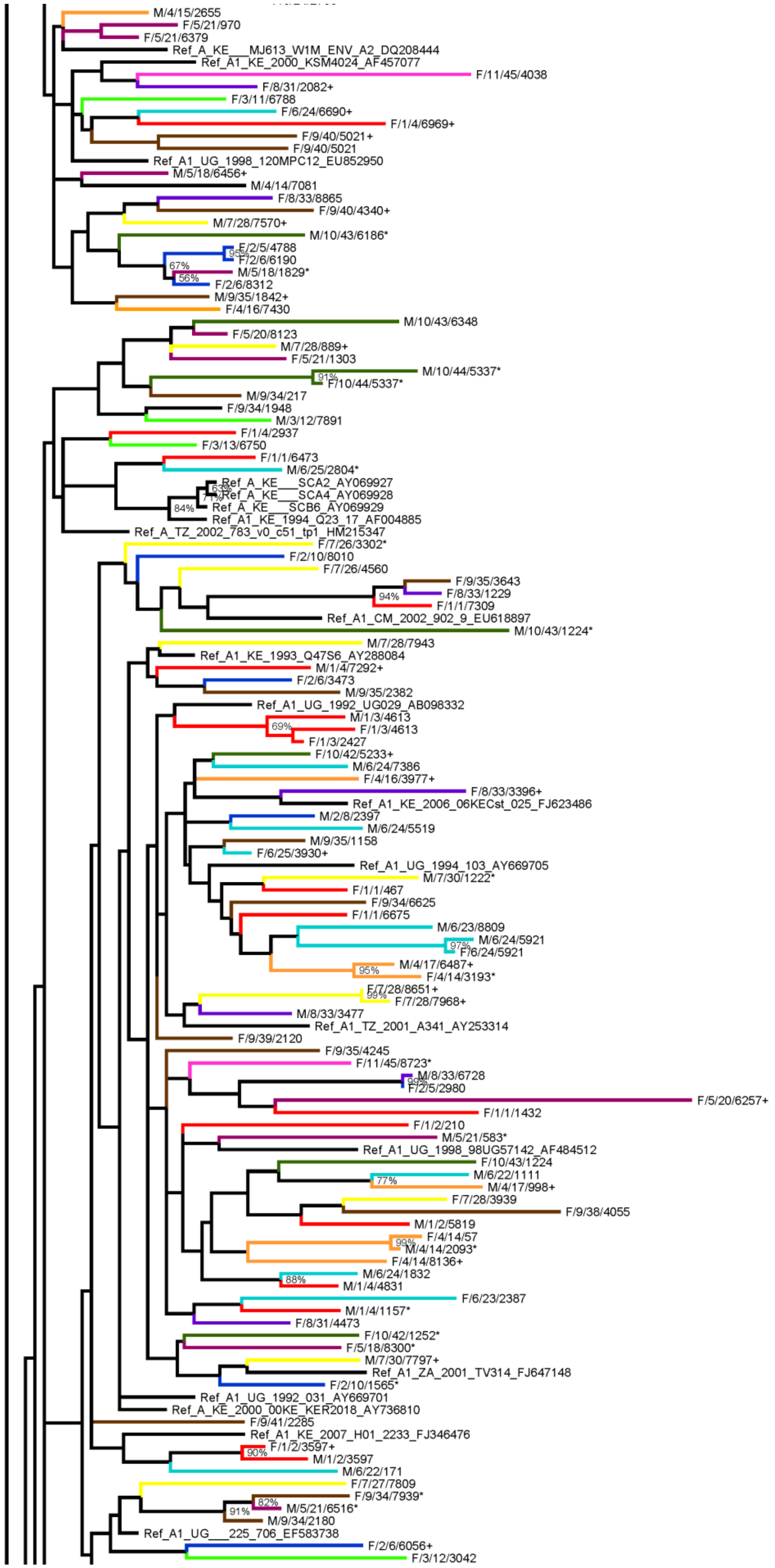

M/4/15/2655
F/5/21/970
F/5/21/6379
Ref_A_KE___MJ613_W1M_ENV_A2_DQ208444
Ref_A1_KE_2000_KSM4024_AF457077
F/11/45/4038
F/8/31/2082+
F/3/11/6788
F/6/24/6690+
F/1/4/6969+
F/9/40/5021+
F/9/40/5021
Ref_A1_UG_1998_120MPC12_EU852950
M/5/18/6456+
M/4/14/7081
F/8/33/8865
F/9/40/4340+
M/7/28/7570+
M/10/43/6186*
F/2/5/4788
F/2/6/6190
67%
M/5/18/1829*
56%
F/2/6/8312
M/9/35/1842+
F/4/16/7430
M/10/43/6348
F/5/20/8123
M/7/28/889+
F/5/21/1303
M/10/44/5337*
91%
F/10/44/5337*
M/9/34/217
F/9/34/1948
M/3/12/7891
F/1/4/2937
F/3/13/6750
F/1/1/6473
M/6/25/2804*
Ref_A_KE___SCA2_AY069927
Ref_A_KE___SCA4_AY069928
84%
Ref_A_KE___SCB6_AY069929
Ref_A1_KE_1994_Q23_17_AF004885
Ref_A_TZ_2002_783_v0_c51_tp1_HM215347
F/7/26/3302*
F/2/10/8010
F/7/26/4560
F/9/35/3643
94%
F/8/33/1229
F/1/1/7309
Ref_A1_CM_2002_902_9_EU618897
M/10/43/1224*
M/7/28/7943
Ref_A1_KE_1993_Q47S6_AY288084
M/1/4/7292+
F/2/6/3473
M/9/35/2382
Ref_A1_UG_1992_UG029_AB098332
M/1/3/4613
69%
F/1/3/4613
F/1/3/2427
F/10/42/5233+
M/6/24/7386
F/4/16/3977+
F/8/33/3396+
Ref_A1_KE_2006_06KECst_025_FJ623486
M/2/8/2397
M/6/24/5519
M/9/35/1158
F/6/25/3930+
Ref_A1_UG_1994_103_AY669705
M/7/30/1222*
F/1/1/467
F/9/34/6625
F/1/1/6675
M/6/23/8809
M/6/24/5921
97%
F/6/24/5921
M/4/17/6487+
95%
F/4/14/3193*
F/7/28/8651+
99%
F/7/28/7968+
M/8/33/3477
Ref_A1_TZ_2001_A341_AY253314
F/9/39/2120
F/9/35/4245
F/11/45/8723*
M/8/33/6728
F/2/5/2980
F/5/20/6257+
F/1/1/1432
F/1/2/210
M/5/21/583*
Ref_A1_UG_1998_98UG57142_AF484512
F/10/43/1224
M/6/22/1111
77%
M/4/17/998+
F/7/28/3939
F/9/38/4055
M/1/2/5819
F/4/14/57
99%
M/4/14/2093*
F/4/14/8136+
M/6/24/1832
88%
M/1/4/4831
F/6/23/2387
M/1/4/1157*
F/8/31/4473
F/10/42/1252*
F/5/18/8300*
M/7/30/7797+
Ref_A1_ZA_2001_TV314_FJ647148
F/2/10/1565*
Ref_A1_UG_1992_031_AY669701
Ref_A_KE_2000_00KE_KER2018_AY736810
F/9/41/2285
Ref_A1_KE_2007_H01_2233_FJ346476
F/1/2/3597+
90%
M/1/2/3597
M/6/22/171
F/7/27/7809
F/9/34/7939*
82%
M/5/21/6516*
91%
M/9/34/2180
Ref_A1_UG___225_706_EF583738
F/2/6/6056+
F/3/12/3042

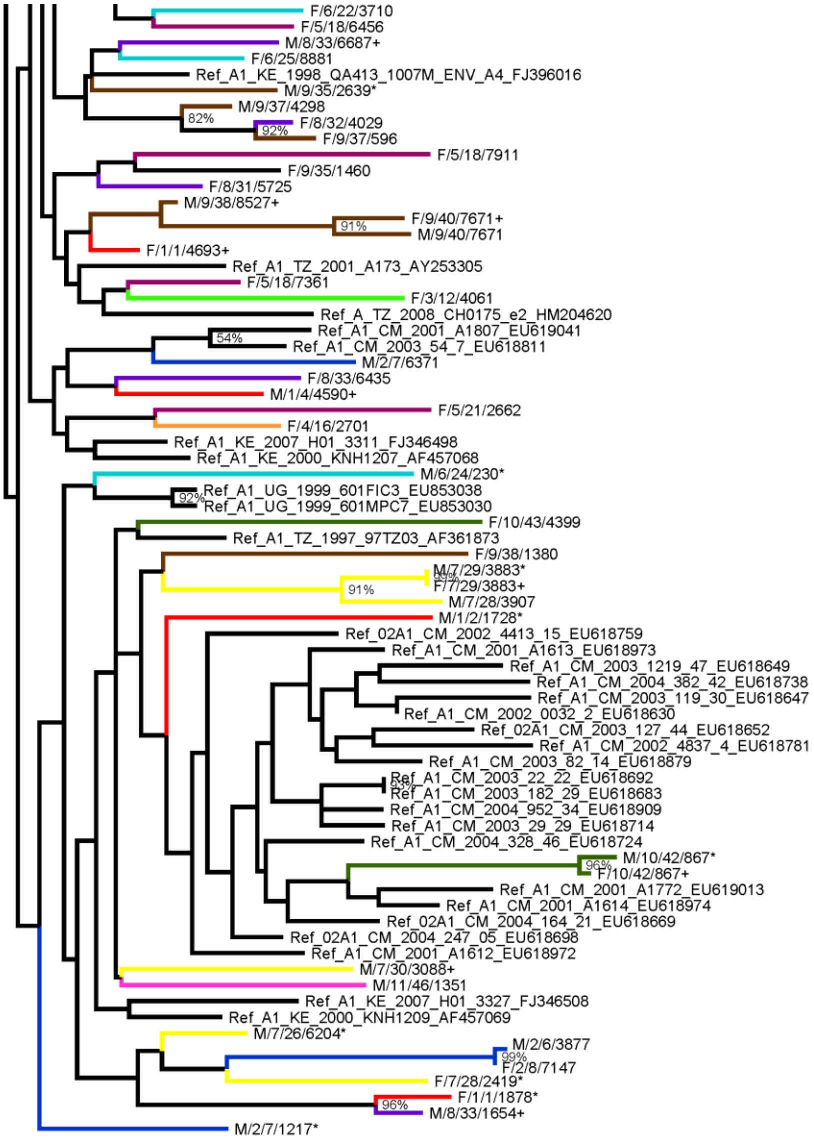
F/6/22/3710
F/5/18/6456
M/8/33/6687+
F/6/25/8881
Ref_A1_KE_1998_QA413_1007M_ENV_A4_FJ396016
M/9/35/2639*
M/9/37/4298
82%
F/8/32/4029
92%
F/9/37/596
F/5/18/7911
F/9/35/1460
F/8/31/5725
M/9/38/8527+
F/9/40/7671+
91%
M/9/40/7671
F/1/1/4693+
Ref_A1_TZ_2001_A173_AY253305
F/5/18/7361
F/3/12/4061
Ref_A_TZ_2008_CH0175_e2_HM204620
Ref_A1_CM_2001_A1807_EU619041
54%
Ref_A1_CM_2003_54_7_EU618811
M/2/7/6371
F/8/33/6435
M/1/4/4590+
F/5/21/2662
F/4/16/2701
Ref_A1_KE_2007_H01_3311_FJ346498
Ref_A1_KE_2000_KNH1207_AF457068
M/6/24/230*
Ref_A1_UG_1999_601FIC3_EU853038
92%
Ref_A1_UG_1999_601MPC7_EU853030
F/10/43/4399
Ref_A1_TZ_1997_97TZ03_AF361873
F/9/38/1380
M/7/29/3883*
F/7/29/3883+
91%
M/7/28/3907
M/1/2/1728*
Ref_02A1_CM_2002_4413_15_EU618759
Ref_A1_CM_2001_A1613_EU618973
Ref_A1_CM_2003_1219_47_EU618649
Ref_A1_CM_2004_382_42_EU618738
Ref_A1_CM_2003_119_30_EU618647
Ref_A1_CM_2002_0032_2_EU618630
Ref_02A1_CM_2003_127_44_EU618652
Ref_A1_CM_2002_4837_4_EU618781
Ref_A1_CM_2003_82_14_EU618879
Ref_A1_CM_2003_22_22_EU618692
Ref_A1_CM_2003_182_29_EU618683
Ref_A1_CM_2004_952_34_EU618909
Ref_A1_CM_2003_29_29_EU618714
Ref_A1_CM_2004_328_46_EU618724
M/10/42/867*
96%
F/10/42/867+
Ref_A1_CM_2001_A1772_EU619013
Ref_A1_CM_2001_A1614_EU618974
Ref_02A1_CM_2004_164_21_EU618669
Ref_02A1_CM_2004_247_05_EU618698
Ref_A1_CM_2001_A1612_EU618972
M/7/30/3088+
M/11/46/1351
Ref_A1_KE_2007_H01_3327_FJ346508
Ref_A1_KE_2000_KNH1209_AF457069
M/7/26/6204*
M/2/6/3877
F/2/8/7147
F/7/28/2419*
F/1/1/1878*
96%
M/8/33/1654+
M/2/7/1217*
