# Supplementary material for: The Role of Viral Introductions in Sustaining Community-Based HIV Epidemics in Rural Uganda: Evidence from Spatial Clustering, Phylogenetics, and Egocentric Transmission Models
Source: PLoS Med. 2014 Mar 4;11(3):e1001610. doi: 10.1371/journal.pmed.1001610 (PMC3942316; doi:10.1371/journal.pmed.1001610)
Supplement: Figure S12 — Maximum likelihood tree (rectangular) of env HIV-1 subtype D sequences. Taxa are labeled using participant gender/geographic region/community/household. Reference sequences (n = 70) are in black, and only bootstrap values ≥50% are shown. Color corresponds to the geographic region. (PDF) [file pmed.1001610.s012.pdf]

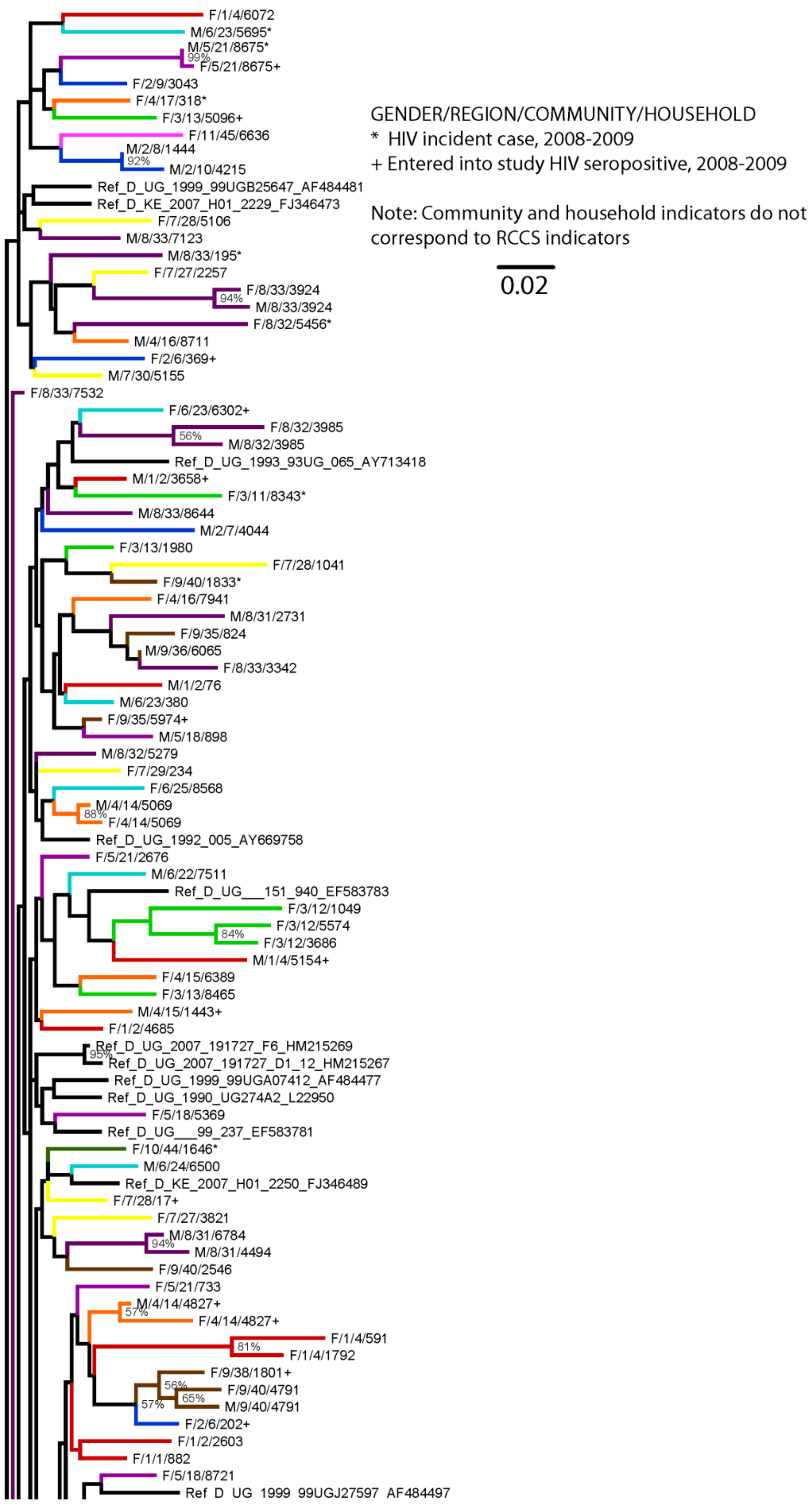
F/1/4/6072
M/6/23/5695*
M/5/21/8675*
99%
F/5/21/8675+
F/2/9/3043
F/4/17/318*
F/3/13/5096+
F/11/45/6636
M/2/8/1444
92%
M/2/10/4215
Ref_D_UG_1999_99UGB25647_AF484481
Ref_D_KE_2007_H01_2229_FJ346473
F/7/28/5106
M/8/33/7123
M/8/33/195*
F/7/27/2257
F/8/33/3924
94%
M/8/33/3924
F/8/32/5456*
M/4/16/8711
F/2/6/369+
M/7/30/5155
F/8/33/7532
F/6/23/6302+
F/8/32/3985
56%
M/8/32/3985
Ref_D_UG_1993_93UG_065_AY713418
M/1/2/3658+
F/3/11/8343*
M/8/33/8644
M/2/7/4044
F/3/13/1980
F/7/28/1041
F/9/40/1833*
F/4/16/7941
M/8/31/2731
F/9/35/824
M/9/36/6065
F/8/33/3342
M/1/2/76
M/6/23/380
F/9/35/5974+
M/5/18/898
M/8/32/5279
F/7/29/234
F/6/25/8568
M/4/14/5069
88%
F/4/14/5069
Ref_D_UG_1992_005_AY669758
F/5/21/2676
M/6/22/7511
Ref_D_UG___151_940_EF583783
F/3/12/1049
F/3/12/5574
84%
F/3/12/3686
M/1/4/5154+
F/4/15/6389
F/3/13/8465
M/4/15/1443+
F/1/2/4685
Ref_D_UG_2007_191727_F6_HM215269
95%
Ref_D_UG_2007_191727_D1_12_HM215267
Ref_D_UG_1999_99UGA07412_AF484477
Ref_D_UG_1990_UG274A2_L22950
F/5/18/5369
Ref_D_UG___99_237_EF583781
F/10/44/1646*
M/6/24/6500
Ref_D_KE_2007_H01_2250_FJ346489
F/7/28/17+
F/7/27/3821
M/8/31/6784
94%
M/8/31/4494
F/9/40/2546
F/5/21/733
M/4/14/4827+
57%
F/4/14/4827+
F/1/4/591
81%
F/1/4/1792
F/9/38/1801+
56%
F/9/40/4791
65%
57%
M/9/40/4791
F/2/6/202+
F/1/2/2603
F/1/1/882
F/5/18/8721
Ref D UG 1999 99UGJ27597 AF484497
GENDER/REGION/COMMUNITY/HOUSEHOLD
* HIV incident case, 2008-2009
+ Entered into study HIV seropositive, 2008-2009
Note: Community and household indicators do not correspond to RCCS indicators
0.02

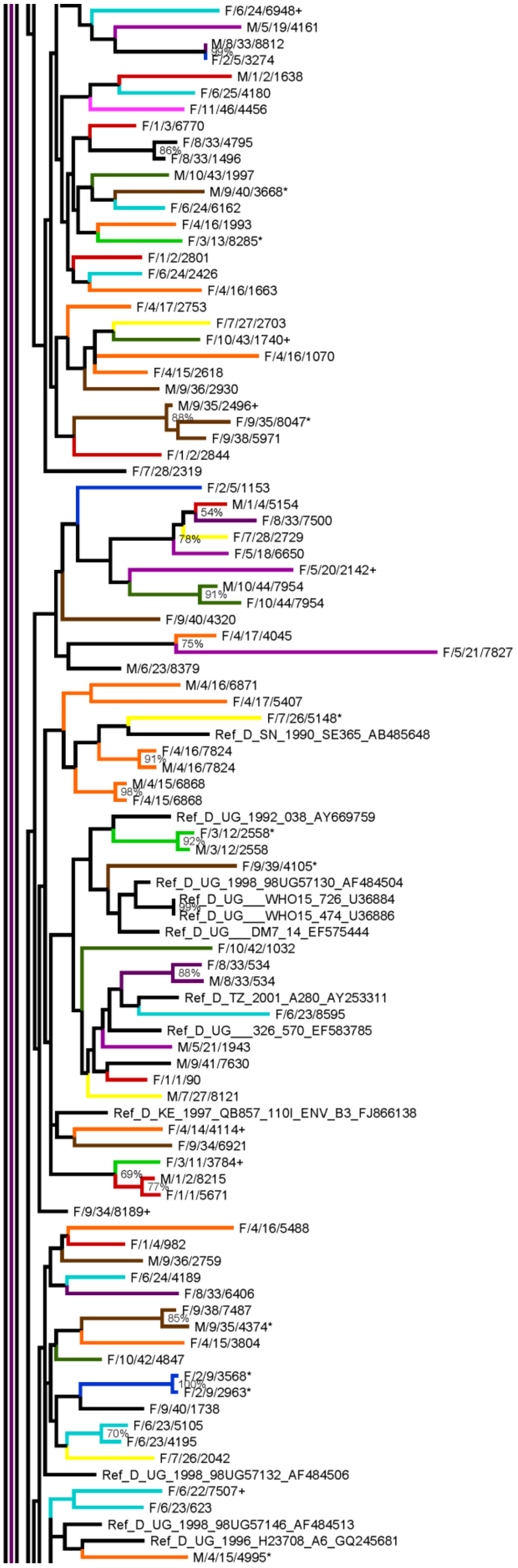
F/6/24/6948+
M/5/19/4161
M/8/33/8812
99%
F/2/5/3274
M/1/2/1638
F/6/25/4180
F/11/46/4456
F/1/3/6770
F/8/33/4795
86%
F/8/33/1496
M/10/43/1997
M/9/40/3668*
F/6/24/6162
F/4/16/1993
F/3/13/8285*
F/1/2/2801
F/6/24/2426
F/4/16/1663
F/4/17/2753
F/7/27/2703
F/10/43/1740+
F/4/16/1070
F/4/15/2618
M/9/36/2930
M/9/35/2496+
88%
F/9/35/8047*
F/9/38/5971
F/1/2/2844
F/7/28/2319
F/2/5/1153
M/1/4/5154
54%
F/8/33/7500
78%
F/7/28/2729
F/5/18/6650
F/5/20/2142+
M/10/44/7954
91%
F/10/44/7954
F/9/40/4320
F/4/17/4045
75%
F/5/21/7827
M/6/23/8379
M/4/16/6871
F/4/17/5407
F/7/26/5148*
Ref_D_SN_1990_SE365_AB485648
F/4/16/7824
91%
M/4/16/7824
M/4/15/6868
98%
F/4/15/6868
Ref_D_UG_1992_038_AY669759
F/3/12/2558*
92%
M/3/12/2558
F/9/39/4105*
Ref_D_UG_1998_98UG57130_AF484504
Ref_D_UG___WHO15_726_U36884
99%
Ref_D_UG___WHO15_474_U36886
Ref_D_UG___DM7_14_EF575444
F/10/42/1032
F/8/33/534
88%
M/8/33/534
Ref_D_TZ_2001_A280_AY253311
F/6/23/8595
Ref_D_UG___326_570_EF583785
M/5/21/1943
M/9/41/7630
F/1/1/90
M/7/27/8121
Ref_D_KE_1997_QB857_110I_ENV_B3_FJ866138
F/4/14/4114+
F/9/34/6921
F/3/11/3784+
69%
M/1/2/8215
77%
F/1/1/5671
F/9/34/8189+
F/4/16/5488
F/1/4/982
M/9/36/2759
F/6/24/4189
F/8/33/6406
F/9/38/7487
85%
M/9/35/4374*
F/4/15/3804
F/10/42/4847
F/2/9/3568*
100%
F/2/9/2963*
F/9/40/1738
F/6/23/5105
70%
F/6/23/4195
F/7/26/2042
Ref_D_UG_1998_98UG57132_AF484506
F/6/22/7507+
F/6/23/623
Ref_D_UG_1998_98UG57146_AF484513
Ref_D_UG_1996_H23708_A6_GQ245681
M/4/15/4995*

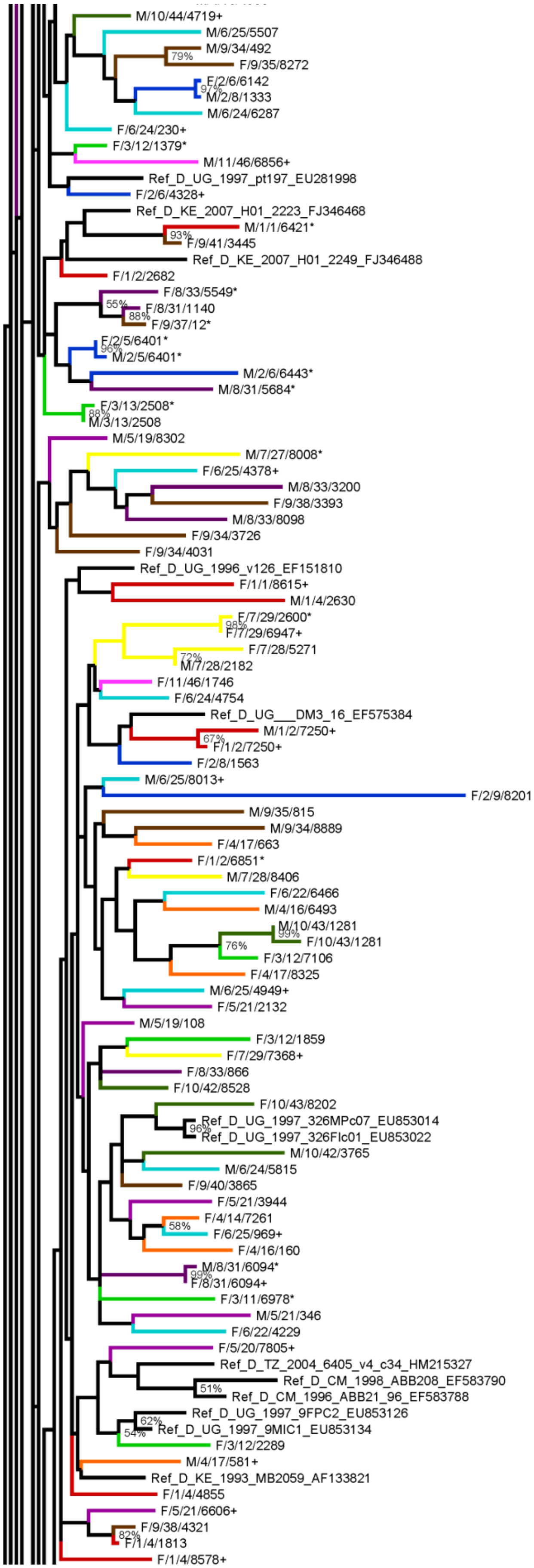
M/10/44/4719+
M/6/25/5507
79%
M/9/34/492
F/9/35/8272
F/2/6/6142
97%
M/2/8/1333
M/6/24/6287
F/6/24/230+
F/3/12/1379*
M/11/46/6856+
Ref_D_UG_1997_pt197_EU281998
F/2/6/4328+
Ref_D_KE_2007_H01_2223_FJ346468
M/1/1/6421*
93%
F/9/41/3445
Ref_D_KE_2007_H01_2249_FJ346488
F/1/2/2682
F/8/33/5549*
55%
F/8/31/1140
88%
F/9/37/12*
F/2/5/6401*
96%
M/2/5/6401*
M/2/6/6443*
M/8/31/5684*
F/3/13/2508*
88%
M/3/13/2508
M/5/19/8302
M/7/27/8008*
F/6/25/4378+
M/8/33/3200
F/9/38/3393
M/8/33/8098
F/9/34/3726
F/9/34/4031
Ref_D_UG_1996_v126_EF151810
F/1/1/8615+
M/1/4/2630
F/7/29/2600*
98%
F/7/29/6947+
F/7/28/5271
72%
M/7/28/2182
F/11/46/1746
F/6/24/4754
Ref_D_UG___DM3_16_EF575384
M/1/2/7250+
67%
F/1/2/7250+
F/2/8/1563
M/6/25/8013+
F/2/9/8201
M/9/35/815
M/9/34/8889
F/4/17/663
F/1/2/6851*
M/7/28/8406
F/6/22/6466
M/4/16/6493
M/10/43/1281
99%
F/10/43/1281
76%
F/3/12/7106
F/4/17/8325
M/6/25/4949+
F/5/21/2132
M/5/19/108
F/3/12/1859
F/7/29/7368+
F/8/33/866
F/10/42/8528
F/10/43/8202
Ref_D_UG_1997_326MPc07_EU853014
96%
Ref_D_UG_1997_326Flc01_EU853022
M/10/42/3765
M/6/24/5815
F/9/40/3865
F/5/21/3944
F/4/14/7261
58%
F/6/25/969+
F/4/16/160
M/8/31/6094*
99%
F/8/31/6094+
F/3/11/6978*
M/5/21/346
F/6/22/4229
F/5/20/7805+
Ref_D_TZ_2004_6405_v4_c34_HM215327
Ref_D_CM_1998_ABB208_EF583790
51%
Ref_D_CM_1996_ABB21_96_EF583788
Ref_D_UG_1997_9FPC2_EU853126
62%
54%
Ref_D_UG_1997_9MIC1_EU853134
F/3/12/2289
M/4/17/581+
Ref_D_KE_1993_MB2059_AF133821
F/1/4/4855
F/5/21/6606+
F/9/38/4321
82%
F/1/4/1813
F/1/4/8578+

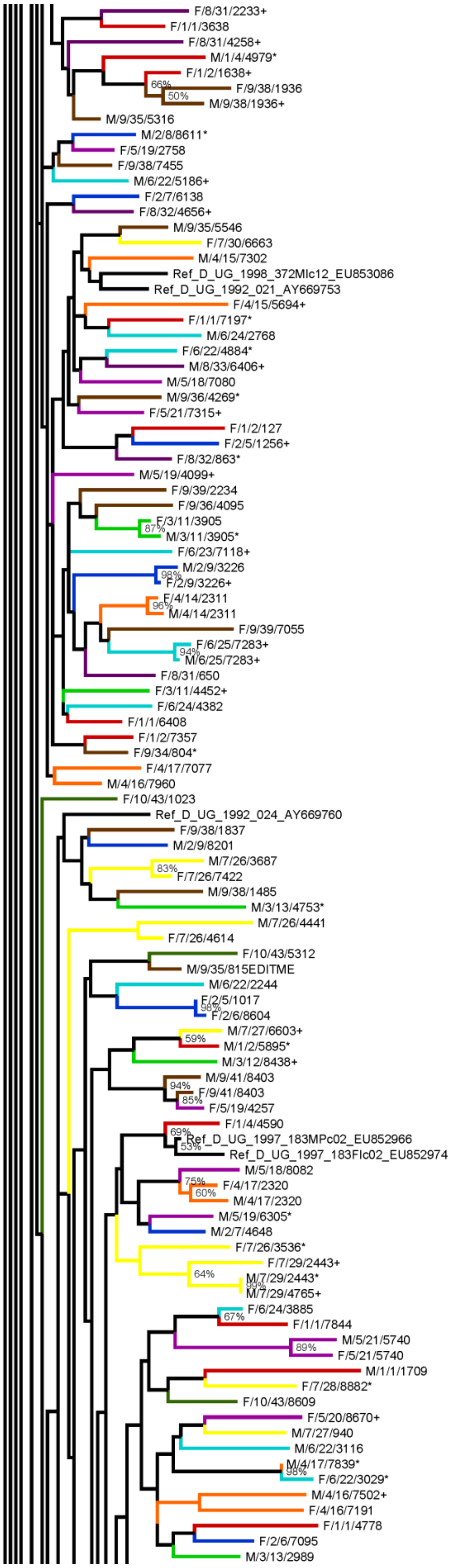
F/8/31/2233+
F/1/1/3638
F/8/31/4258+
M/1/4/4979*
F/1/2/1638+
66%
F/9/38/1936
50%
M/9/38/1936+
M/9/35/5316
M/2/8/8611*
F/5/19/2758
F/9/38/7455
M/6/22/5186+
F/2/7/6138
F/8/32/4656+
M/9/35/5546
F/7/30/6663
M/4/15/7302
Ref_D_UG_1998_372MIc12_EU853086
Ref_D_UG_1992_021_AY669753
F/4/15/5694+
F/1/1/7197*
M/6/24/2768
F/6/22/4884*
M/8/33/6406+
M/5/18/7080
M/9/36/4269*
F/5/21/7315+
F/1/2/127
F/2/5/1256+
F/8/32/863*
M/5/19/4099+
F/9/39/2234
F/9/36/4095
F/3/11/3905
87%
M/3/11/3905*
F/6/23/7118+
M/2/9/3226
98%
F/2/9/3226+
F/4/14/2311
96%
M/4/14/2311
F/9/39/7055
F/6/25/7283+
94%
M/6/25/7283+
F/8/31/650
F/3/11/4452+
F/6/24/4382
F/1/1/6408
F/1/2/7357
F/9/34/804*
F/4/17/7077
M/4/16/7960
F/10/43/1023
Ref_D_UG_1992_024_AY669760
F/9/38/1837
M/2/9/8201
M/7/26/3687
83%
F/7/26/7422
M/9/38/1485
M/3/13/4753*
M/7/26/4441
F/7/26/4614
F/10/43/5312
M/9/35/815EDITME
M/6/22/2244
F/2/5/1017
98%
F/2/6/8604
M/7/27/6603+
59%
M/1/2/5895*
M/3/12/8438+
M/9/41/8403
94%
F/9/41/8403
85%
F/5/19/4257
F/1/4/4590
69%
Ref_D_UG_1997_183MPc02_EU852966
53%
Ref_D_UG_1997_183FIc02_EU852974
M/5/18/8082
75%
F/4/17/2320
60%
M/4/17/2320
M/5/19/6305*
M/2/7/4648
F/7/26/3536*
F/7/29/2443+
64%
M/7/29/2443*
M/7/29/4765+
F/6/24/3885
67%
F/1/1/7844
M/5/21/5740
89%
F/5/21/5740
M/1/1/1709
F/7/28/8882*
F/10/43/8609
F/5/20/8670+
M/7/27/940
M/6/22/3116
M/4/17/7839*
98%
F/6/22/3029*
M/4/16/7502+
F/4/16/7191
F/1/1/4778
F/2/6/7095
M/3/13/2989

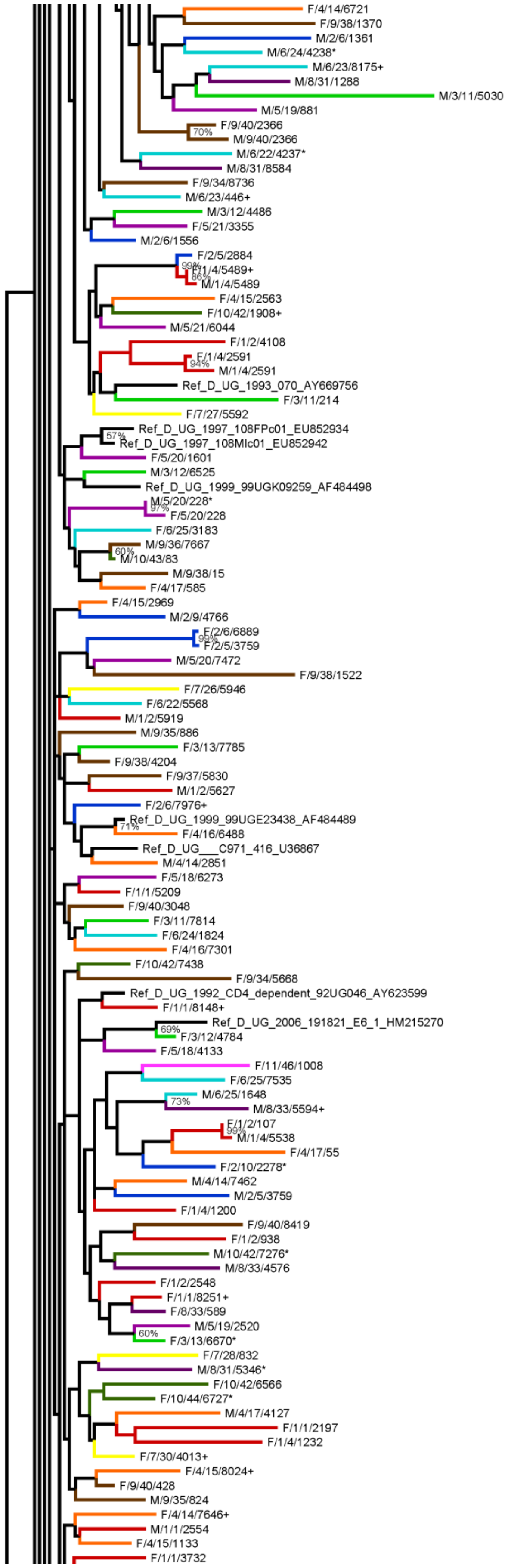
F/4/14/6721
F/9/38/1370
M/2/6/1361
M/6/24/4238*
M/6/23/8175+
M/8/31/1288
M/3/11/5030
M/5/19/881
F/9/40/2366
70%
M/9/40/2366
M/6/22/4237*
M/8/31/8584
F/9/34/8736
M/6/23/446+
M/3/12/4486
F/5/21/3355
M/2/6/1556
F/2/5/2884
99%
F/1/4/5489+
86%
M/1/4/5489
F/4/15/2563
F/10/42/1908+
M/5/21/6044
F/1/2/4108
F/1/4/2591
94%
M/1/4/2591
Ref_D_UG_1993_070_AY669756
F/3/11/214
F/7/27/5592
Ref_D_UG_1997_108FPc01_EU852934
57%
Ref_D_UG_1997_108MIc01_EU852942
F/5/20/1601
M/3/12/6525
Ref_D_UG_1999_99UGK09259_AF484498
M/5/20/228*
97%
F/5/20/228
F/6/25/3183
M/9/36/7667
60%
M/10/43/83
M/9/38/15
F/4/17/585
F/4/15/2969
M/2/9/4766
F/2/6/6889
95%
F/2/5/3759
M/5/20/7472
F/9/38/1522
F/7/26/5946
F/6/22/5568
M/1/2/5919
M/9/35/886
F/3/13/7785
F/9/38/4204
F/9/37/5830
M/1/2/5627
F/2/6/7976+
Ref_D_UG_1999_99UGE23438_AF484489
71%
F/4/16/6488
Ref_D_UG___C971_416_U36867
M/4/14/2851
F/5/18/6273
F/1/1/5209
F/9/40/3048
F/3/11/7814
F/6/24/1824
F/4/16/7301
F/10/42/7438
F/9/34/5668
Ref_D_UG_1992_CD4_dependent_92UG046_AY623599
F/1/1/8148+
Ref_D_UG_2006_191821_E6_1_HM215270
69%
F/3/12/4784
F/5/18/4133
F/11/46/1008
F/6/25/7535
M/6/25/1648
73%
M/8/33/5594+
F/1/2/107
99%
M/1/4/5538
F/4/17/55
F/2/10/2278*
M/4/14/7462
M/2/5/3759
F/1/4/1200
F/9/40/8419
F/1/2/938
M/10/42/7276*
M/8/33/4576
F/1/2/2548
F/1/1/8251+
F/8/33/589
M/5/19/2520
60%
F/3/13/6670*
F/7/28/832
M/8/31/5346*
F/10/42/6566
F/10/44/6727*
M/4/17/4127
F/1/1/2197
F/1/4/1232
F/7/30/4013+
F/4/15/8024+
F/9/40/428
M/9/35/824
F/4/14/7646+
M/1/1/2554
F/4/15/1133
F/1/1/3732

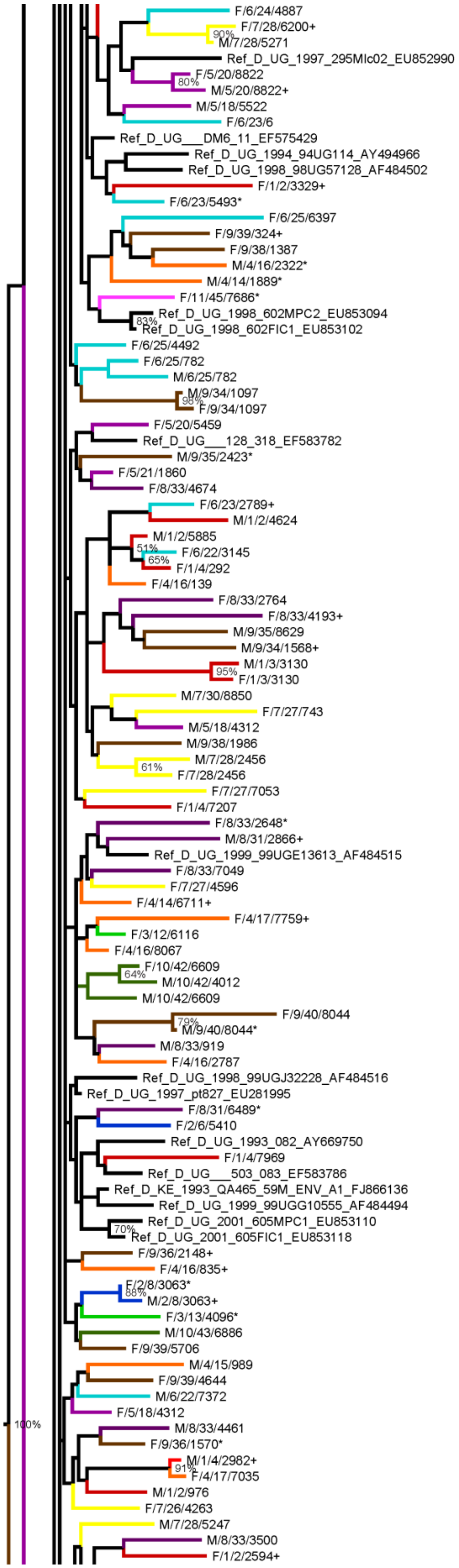
F/6/24/4887
F/7/28/6200+
90%
M/7/28/5271
Ref_D_UG_1997_295MIc02_EU852990
F/5/20/8822
80%
M/5/20/8822+
M/5/18/5522
F/6/23/6
Ref_D_UG___DM6_11_EF575429
Ref_D_UG_1994_94UG114_AY494966
Ref_D_UG_1998_98UG57128_AF484502
F/1/2/3329+
F/6/23/5493*
F/6/25/6397
F/9/39/324+
F/9/38/1387
M/4/16/2322*
M/4/14/1889*
F/11/45/7686*
Ref_D_UG_1998_602MPC2_EU853094
83%
Ref_D_UG_1998_602FIC1_EU853102
F/6/25/4492
F/6/25/782
M/6/25/782
M/9/34/1097
98%
F/9/34/1097
F/5/20/5459
Ref_D_UG___128_318_EF583782
M/9/35/2423*
F/5/21/1860
F/8/33/4674
F/6/23/2789+
M/1/2/4624
M/1/2/5885
51%
F/6/22/3145
65%
F/1/4/292
F/4/16/139
F/8/33/2764
F/8/33/4193+
M/9/35/8629
M/9/34/1568+
M/1/3/3130
95%
F/1/3/3130
M/7/30/8850
F/7/27/743
M/5/18/4312
M/9/38/1986
M/7/28/2456
61%
F/7/28/2456
F/7/27/7053
F/1/4/7207
F/8/33/2648*
M/8/31/2866+
Ref_D_UG_1999_99UGE13613_AF484515
F/8/33/7049
F/7/27/4596
F/4/14/6711+
F/4/17/7759+
F/3/12/6116
F/4/16/8067
F/10/42/6609
64%
M/10/42/4012
M/10/42/6609
F/9/40/8044
79%
M/9/40/8044*
M/8/33/919
F/4/16/2787
Ref_D_UG_1998_99UGJ32228_AF484516
Ref_D_UG_1997_pt827_EU281995
F/8/31/6489*
F/2/6/5410
Ref_D_UG_1993_082_AY669750
F/1/4/7969
Ref_D_UG___503_083_EF583786
Ref_D_KE_1993_QA465_59M_ENV_A1_FJ866136
Ref_D_UG_1999_99UGG10555_AF484494
Ref_D_UG_2001_605MPC1_EU853110
70%
Ref_D_UG_2001_605FIC1_EU853118
F/9/36/2148+
F/4/16/835+
F/2/8/3063*
88%
M/2/8/3063+
F/3/13/4096*
M/10/43/6886
F/9/39/5706
M/4/15/989
F/9/39/4644
M/6/22/7372
F/5/18/4312
100%
M/8/33/4461
F/9/36/1570*
M/1/4/2982+
91%
F/4/17/7035
M/1/2/976
F/7/26/4263
M/7/28/5247
M/8/33/3500
F/1/2/2594+

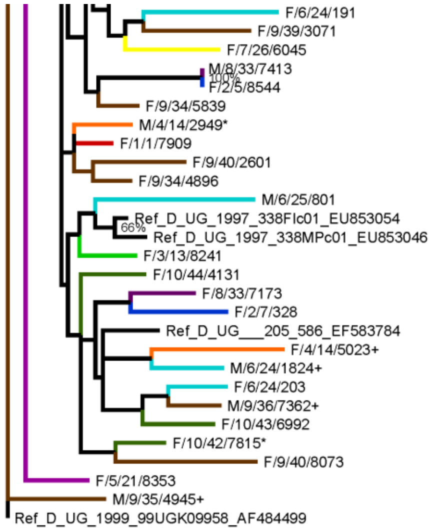

F/6/24/191
F/9/39/3071
F/7/26/6045
M/8/33/7413
100%
F/2/5/8544
F/9/34/5839
M/4/14/2949*
F/1/1/7909
F/9/40/2601
F/9/34/4896
M/6/25/801
Ref_D_UG_1997_338FIc01_EU853054
66%
Ref_D_UG_1997_338MPc01_EU853046
F/3/13/8241
F/10/44/4131
F/8/33/7173
F/2/7/328
Ref_D_UG___205_586_EF583784
F/4/14/5023+
M/6/24/1824+
F/6/24/203
M/9/36/7362+
F/10/43/6992
F/10/42/7815*
F/9/40/8073
F/5/21/8353
M/9/35/4945+
Ref_D_UG_1999_99UGK09958_AF484499
