# Supplementary material for: The Role of Viral Introductions in Sustaining Community-Based HIV Epidemics in Rural Uganda: Evidence from Spatial Clustering, Phylogenetics, and Egocentric Transmission Models
Source: PLoS Med. 2014 Mar 4;11(3):e1001610. doi: 10.1371/journal.pmed.1001610 (PMC3942316; doi:10.1371/journal.pmed.1001610)
Supplement: Figure S13 — Maximum likelihood tree (rectangular) of env HIV-1 subtype C sequences. Taxa are labeled using participant gender/geographic region/community/household. Reference sequences (n = 37) are in black, and only bootstrap values ≥50% are shown. Color corresponds to the geographic regions. (PDF) [file pmed.1001610.s013.pdf]

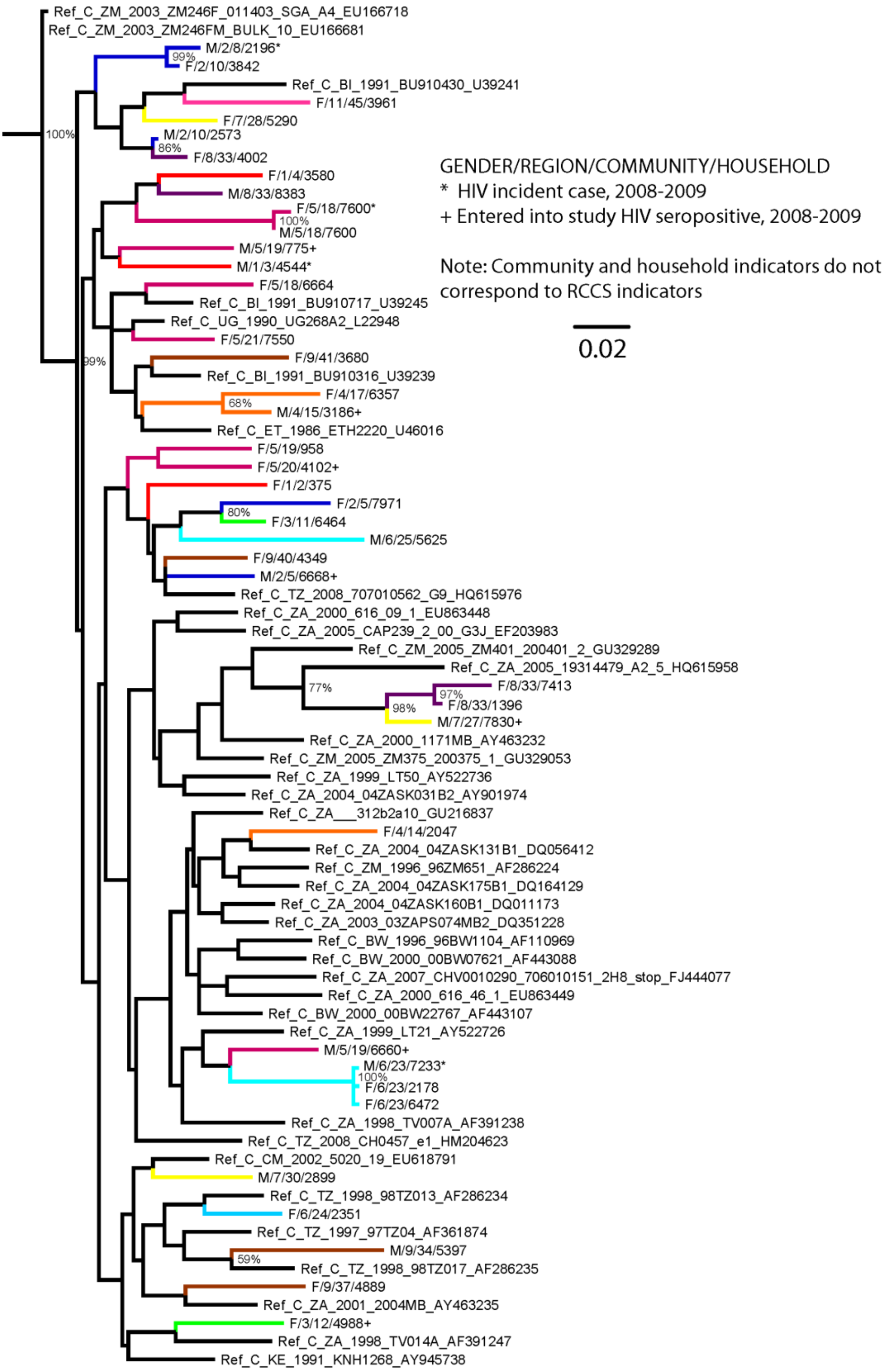
Ref_C_ZM_2003_ZM246F_011403_SGA_A4_EU166718
Ref_C_ZM_2003_ZM246FM_BULK_10_EU166681
M/2/8/2196*
99%
F/2/10/3842
Ref_C_BI_1991_BU910430_U39241
F/11/45/3961
F/7/28/5290
100%
M/2/10/2573
86%
F/8/33/4002
F/1/4/3580
M/8/33/8383
F/5/18/7600*
100%
M/5/18/7600
M/5/19/775+
M/1/3/4544*
F/5/18/6664
Ref_C_BI_1991_BU910717_U39245
Ref_C_UG_1990_UG268A2_L22948
F/5/21/7550
99%
F/9/41/3680
Ref_C_BI_1991_BU910316_U39239
F/4/17/6357
68%
M/4/15/3186+
Ref_C_ET_1986_ETH2220_U46016
F/5/19/958
F/5/20/4102+
F/1/2/375
F/2/5/7971
80%
F/3/11/6464
M/6/25/5625
F/9/40/4349
M/2/5/6668+
Ref_C_TZ_2008_707010562_G9_HQ615976
Ref_C_ZA_2000_616_09_1_EU863448
Ref_C_ZA_2005_CAP239_2_00_G3J_EF203983
Ref_C_ZM_2005_ZM401_200401_2_GU329289
Ref_C_ZA_2005_19314479_A2_5_HQ615958
77%
F/8/33/7413
97%
F/8/33/1396
98%
M/7/27/7830+
Ref_C_ZA_2000_1171MB_AY463232
Ref_C_ZM_2005_ZM375_200375_1_GU329053
Ref_C_ZA_1999_LT50_AY522736
Ref_C_ZA_2004_04ZASK031B2_AY901974
Ref_C_ZA___312b2a10_GU216837
F/4/14/2047
Ref_C_ZA_2004_04ZASK131B1_DQ056412
Ref_C_ZM_1996_96ZM651_AF286224
Ref_C_ZA_2004_04ZASK175B1_DQ164129
Ref_C_ZA_2004_04ZASK160B1_DQ011173
Ref_C_ZA_2003_03ZAPS074MB2_DQ351228
Ref_C_BW_1996_96BW1104_AF110969
Ref_C_BW_2000_00BW07621_AF443088
Ref_C_ZA_2007_CHV0010290_706010151_2H8_stop_FJ444077
Ref_C_ZA_2000_616_46_1_EU863449
Ref_C_BW_2000_00BW22767_AF443107
Ref_C_ZA_1999_LT21_AY522726
M/5/19/6660+
M/6/23/7233*
100%
F/6/23/2178
F/6/23/6472
Ref_C_ZA_1998_TV007A_AF391238
Ref_C_TZ_2008_CH0457_e1_HM204623
Ref_C_CM_2002_5020_19_EU618791
M/7/30/2899
Ref_C_TZ_1998_98TZ013_AF286234
F/6/24/2351
Ref_C_TZ_1997_97TZ04_AF361874
M/9/34/5397
59%
Ref_C_TZ_1998_98TZ017_AF286235
F/9/37/4889
Ref_C_ZA_2001_2004MB_AY463235
F/3/12/4988+
Ref_C_ZA_1998_TV014A_AF391247
Ref_C_KE_1991_KNH1268_AY945738
GENDER/REGION/COMMUNITY/HOUSEHOLD
* HIV incident case, 2008-2009
+ Entered into study HIV seropositive, 2008-2009
Note: Community and household indicators do not correspond to RCCS indicators
0.02
