# Supplementary material for: The Role of Viral Introductions in Sustaining Community-Based HIV Epidemics in Rural Uganda: Evidence from Spatial Clustering, Phylogenetics, and Egocentric Transmission Models
Source: PLoS Med. 2014 Mar 4;11(3):e1001610. doi: 10.1371/journal.pmed.1001610 (PMC3942316; doi:10.1371/journal.pmed.1001610)
Supplement: Table S1 — Accession numbers for Los Alamos National Laboratory HIV Sequence Database reference sequences used for maximum likelihood and Bayesian phylogenetic analyses. This table includes the accession numbers, geographic location, year of collection, and HIV-1 subtype for each gag and env reference sequence used in phylogenetic analyses. (DOCX) [file pmed.1001610.s014.docx]

| **Table S1. Accession numbers for HIVDB reference sequences used for maximum likelihood and Bayesian phylogenetic analyses.** | | | | |
| --- | --- | --- | --- | --- |
| Accession | Country | Year | HIV-1 Subtype | Gene region |
| AB098330 | UG |  | A1 | *gag* |
| AB253421 | RW | 1992 | A1 | *gag* |
| AB253428 | UG | 1992 | A1 | *gag* |
| AB287376 | RW | 1992 | A1 | *gag* |
| AB485632 | UG |  | A1 | *gag* |
| AB485650 | UG | 1991 | D | *gag* |
| AF004885 | KE | 1994 | A1 | *gag* |
| AF133821 | KE | 1993 | D | *gag* |
| AF391251 | ZA | 1998 | C | *gag* |
| AF443075 | BW | 1996 | C | *gag* |
| AF457055 | KE | 2000 | A1 | *gag* |
| AF457065 | KE | 1999 | A1 | *gag* |
| AF457067 | KE | 2000 | A1 | *gag* |
| AF457068 | KE | 2000 | A1 | *gag* |
| AF457075 | KE | 1999 | A1 | *gag* |
| AF457081 | KE | 2000 | A1 | *gag* |
| AF457090 | KE | 2001 | D | *gag* |
| AF484478 | UG | 1999 | A1 | *gag* |
| AF484480 | UG | 1999 | D | *gag* |
| AF484481 | UG | 1999 | D | *gag* |
| AF484483 | UG | 1999 | D | *gag* |
| AF484486 | UG | 1999 | D | *gag* |
| AF484489 | UG | 1999 | D | *gag* |
| AF484493 | UG | 1999 | A1 | *gag* |
| AF484495 | UG | 1999 | D | *gag* |
| AF484497 | UG | 1999 | D | *gag* |
| AF484498 | UG | 1999 | D | *gag* |
| AF484499 | UG | 1999 | D | *gag* |
| AF484506 | UG | 1998 | D | *gag* |
| AF484507 | UG | 1998 | A1 | *gag* |
| AF484511 | UG | 1998 | D | *gag* |
| AF484513 | UG | 1998 | D | *gag* |
| AF484514 | UG | 1998 | D | *gag* |
| AF484515 | UG | 1999 | D | *gag* |
| AF484516 | UG | 1998 | D | *gag* |
| AF484518 | UG | 1999 | D | *gag* |
| AY140566 | GA | 1997 | D | *gag* |
| AY253321 | TZ | 2001 | C | *gag* |
| AY304496 | UG | 1999 | D | *gag* |
| AY322184 | KE | 1986 | A1 | *gag* |
| AY521630 | SN | 1996 | A | *gag* |
| AY521631 | SN | 2001 | A | *gag* |
| AY521632 | SN | 1996 | A1 | *gag* |
| AY772956 | KE | 1997 | D | *gag* |
| AY772957 | KE | 1997 | D | *gag* |
| AY772970 | KE | 1998 | A | *gag* |
| AY772971 | KE | 1998 | A | *gag* |
| AY772985 | KE | 1999 | A | *gag* |
| AY772986 | KE | 1998 | A | *gag* |
| AY803355 | UG | 2001 | A1 | *gag* |
| AY803359 | UG | 2003 | D | *gag* |
| AY803362 | UG | 2003 | D | *gag* |
| AY803364 | UG | 2003 | A1 | *gag* |
| AY803367 | UG | 2003 | D | *gag* |
| AY803368 | UG | 2003 | D | *gag* |
| AY803372 | UG | 2003 | D | *gag* |
| AY803379 | UG | 2003 | D | *gag* |
| AY803380 | UG | 2003 | A1 | *gag* |
| AY803382 | UG | 2003 | A1 | *gag* |
| AY803385 | UG | 2003 | A1 | *gag* |
| AY803390 | UG | 2003 | A1 | *gag* |
| AY803398 | UG | 2004 | D | *gag* |
| AY803401 | UG | 2002 | A1 | *gag* |
| AY803403 | UG | 2002 | A1 | *gag* |
| DQ367268 | KE | 1999 | A1 | *gag* |
| DQ367269 | KE | 1999 | A1 | *gag* |
| DQ367271 | KE | 1999 | A1 | *gag* |
| DQ367273 | KE | 1999 | A1 | *gag* |
| DQ367281 | KE | 1999 | A1 | *gag* |
| DQ367283 | KE | 1999 | A1 | *gag* |
| DQ367285 | KE | 1999 | D | *gag* |
| DQ367286 | KE | 1999 | A1 | *gag* |
| DQ367288 | KE | 1999 | A1 | *gag* |
| DQ367290 | KE | 1999 | A1 | *gag* |
| DQ367294 | KE | 1999 | A1 | *gag* |
| DQ367295 | KE | 1999 | A1 | *gag* |
| DQ793008 | ZM | 2001 | C | *gag* |
| EF186092 | UG | 2005 | D | *gag* |
| EF186094 | UG | 2005 | A1 | *gag* |
| EF186095 | UG | 2005 | D | *gag* |
| EF186100 | UG | 2005 | D | *gag* |
| EF186101 | UG | 2005 | A1 | *gag* |
| EF186102 | UG | 2005 | D | *gag* |
| EF186103 | UG | 2005 | A1 | *gag* |
| EF186107 | UG | 2005 | C | *gag* |
| EF186108 | UG | 2005 | A1 | *gag* |
| EF186109 | UG | 2005 | A1 | *gag* |
| EF186111 | UG | 2005 | A1 | *gag* |
| EF186155 | UG | 2005 | D | *gag* |
| EF186156 | UG | 2005 | A1 | *gag* |
| EF186159 | UG | 2005 | D | *gag* |
| EF186164 | UG | 2005 | D | *gag* |
| EF186166 | UG | 2005 | A1 | *gag* |
| EF186168 | UG | 2005 | D | *gag* |
| EF186174 | UG | 2005 | A1 | *gag* |
| EF186176 | UG | 2005 | A1 | *gag* |
| EF186177 | UG | 2005 | A1 | *gag* |
| EU110094 | KE | 2002 | A1 | *gag* |
| FJ606116 | ZM | 2005 | C | *gag* |
| FJ606131 | ZM | 1999 | C | *gag* |
| FJ606146 | ZM | 2005 | C | *gag* |
| FJ606153 | ZM | 2005 | C | *gag* |
| FJ606156 | ZM | 2005 | C | *gag* |
| FJ606162 | ZM | 2005 | C | *gag* |
| FJ606181 | ZM | 2000 | C | *gag* |
| FJ606185 | ZM | 1999 | C | *gag* |
| FJ606193 | ZM | 2006 | C | *gag* |
| FJ606229 | ZM | 2003 | C | *gag* |
| FJ606274 | ZM | 2003 | C | *gag* |
| FJ606340 | ZM | 2006 | C | *gag* |
| FJ606410 | ZM | 2005 | C | *gag* |
| FJ606430 | ZM | 2005 | C | *gag* |
| FJ606437 | ZM | 2005 | C | *gag* |
| FJ606441 | ZM | 2005 | C | *gag* |
| FJ623476 | KE | 2006 | A1 | *gag* |
| FJ853502 | TZ | 2003 | C | *gag* |
| FJ853510 | TZ | 2003 | A1 | *gag* |
| FJ853511 | TZ | 2003 | A1 | *gag* |
| FJ853532 | TZ | 2003 | C | *gag* |
| FJ853572 | TZ | 2003 | A1 | *gag* |
| FJ853577 | TZ | 2003 | A1 | *gag* |
| FJ853588 | TZ | 2003 | D | *gag* |
| GQ429865 | KE | 1996 | A1 | *gag* |
| GQ429897 | KE | 1995 | A1 | *gag* |
| GQ430144 | KE | 2000 | D | *gag* |
| GQ430207 | KE | 1987 | C | *gag* |
| GQ430231 | KE | 1987 | C | *gag* |
| GQ430263 | KE | 1987 | D | *gag* |
| GQ430293 | KE | 1992 | A1 | *gag* |
| GQ430325 | KE | 1988 | D | *gag* |
| GQ430355 | KE | 2001 | A1 | *gag* |
| GQ430447 | KE | 1995 | A1 | *gag* |
| GQ430614 | KE | 1995 | A1 | *gag* |
| GQ430768 | KE | 1994 | A1 | *gag* |
| GQ430832 | KE | 1996 | A1 | *gag* |
| GQ430893 | KE | 1998 | D | *gag* |
| GQ430957 | KE | 1989 | D | *gag* |
| GQ430988 | KE | 2002 | A1 | *gag* |
| GQ431310 | KE | 1995 | A1 | *gag* |
| GQ431326 | KE | 1995 | D | *gag* |
| GQ431480 | KE | 1995 | D | *gag* |
| GQ431601 | KE | 1995 | C | *gag* |
| GQ431939 | KE | 1996 | A1 | *gag* |
| GQ432033 | KE | 1995 | A1 | *gag* |
| GQ432294 | KE | 1987 | A1 | *gag* |
| GQ432337 | KE | 1987 | A1 | *gag* |
| GQ432657 | KE | 1995 | A1 | *gag* |
| GQ432683 | KE | 1995 | A1 | *gag* |
| GQ432715 | KE | 1996 | A1 | *gag* |
| GQ432747 | KE | 1998 | A1 | *gag* |
| HM593171 | ZA | 2004 | C | *gag* |
| HM593202 | ZA | 2004 | C | *gag* |
| HM593218 | ZA | 2003 | C | *gag* |
| HM593270 | ZA | 2005 | C | *gag* |
| HM593282 | ZA | 2004 | C | *gag* |
| HM593313 | ZA | 2005 | C | *gag* |
| HM593318 | ZA | 2005 | C | *gag* |
| HM593336 | ZA | 2005 | C | *gag* |
| HM593342 | ZA | 2005 | C | *gag* |
| HM593409 | ZA | 2006 | C | *gag* |
| HM593454 | ZA | 2006 | C | *gag* |
| HQ702687 | UG | 2008 | A1 | *gag* |
| HQ702688 | UG | 2008 | A1 | *gag* |
| HQ702690 | UG | 2008 | D | *gag* |
| HQ702691 | UG | 2008 | D | *gag* |
| HQ702694 | UG | 2008 | D | *gag* |
| HQ702695 | UG | 2008 | D | *gag* |
| HQ702708 | UG | 2008 | D | *gag* |
| HQ702723 | UG | 2008 | A1 | *gag* |
| HQ702725 | UG | 2008 | D | *gag* |
| L11768 | KE |  | A | *gag* |
| L11770 | KE |  | A | *gag* |
| L11771 | KE |  | D | *gag* |
| L11773 | KE |  | A | *gag* |
| L11774 | KE | 1990 | A | *gag* |
| L11775 | KE |  | A | *gag* |
| L11784 | CD |  | D | *gag* |
| L11787 | CD |  | C | *gag* |
| L11788 | BI |  | A | *gag* |
| L11801 | UG | 1991 | D | *gag* |
| M62320 | UG | 1985 | A1 | *gag* |
| U88824 | UG | 1994 | D | *gag* |
| AB098332 | UG | 1992 | A1 | *env* |
| AB287376 | RW | 1992 | A1 | *env* |
| AB287378 | RW | 1993 | A1 | *env* |
| AB485632 | UG |  | A1 | *env* |
| AB485648 | SN | 1990 | D | *env* |
| AF004885 | KE | 1994 | A1 | *env* |
| AF110969 | BW | 1996 | C | *env* |
| AF133821 | KE | 1993 | D | *env* |
| AF286224 | ZM | 1996 | C | *env* |
| AF286234 | TZ | 1998 | C | *env* |
| AF286235 | TZ | 1998 | C | *env* |
| AF361873 | TZ | 1997 | A1 | *env* |
| AF361874 | TZ | 1997 | C | *env* |
| AF391238 | ZA | 1998 | C | *env* |
| AF391247 | ZA | 1998 | C | *env* |
| AF407148 | KE | 1995 | A1 | *env* |
| AF407154 | KE | 1995 | A1 | *env* |
| AF407160 | KE | 1994 | A1 | *env* |
| AF443088 | BW | 2000 | C | *env* |
| AF443107 | BW | 2000 | C | *env* |
| AF457055 | KE | 2000 | A1 | *env* |
| AF457056 | KE | 2000 | A1 | *env* |
| AF457062 | KE | 1999 | A1 | *env* |
| AF457068 | KE | 2000 | A1 | *env* |
| AF457069 | KE | 2000 | A1 | *env* |
| AF457070 | KE | 2000 | A1 | *env* |
| AF457075 | KE | 1999 | A1 | *env* |
| AF457077 | KE | 2000 | A1 | *env* |
| AF457079 | KE | 2000 | A1 | *env* |
| AF457083 | KE | 2000 | A1 | *env* |
| AF457086 | KE | 2000 | A1 | *env* |
| AF484477 | UG | 1999 | D | *env* |
| AF484478 | UG | 1999 | A1 | *env* |
| AF484481 | UG | 1999 | D | *env* |
| AF484489 | UG | 1999 | D | *env* |
| AF484491 | UG | 1999 | A1 | *env* |
| AF484494 | UG | 1999 | D | *env* |
| AF484497 | UG | 1999 | D | *env* |
| AF484498 | UG | 1999 | D | *env* |
| AF484499 | UG | 1999 | D | *env* |
| AF484502 | UG | 1998 | D | *env* |
| AF484504 | UG | 1998 | D | *env* |
| AF484506 | UG | 1998 | D | *env* |
| AF484512 | UG | 1998 | A1 | *env* |
| AF484513 | UG | 1998 | D | *env* |
| AF484515 | UG | 1999 | D | *env* |
| AF484516 | UG | 1998 | D | *env* |
| AM279343 | CM | 1997 | A | *env* |
| AM279350 | CM | 1999 | G | *env* |
| AY069927 | KE |  | A | *env* |
| AY069928 | KE |  | A | *env* |
| AY069929 | KE |  | A | *env* |
| AY253305 | TZ | 2001 | A1 | *env* |
| AY253311 | TZ | 2001 | D | *env* |
| AY253314 | TZ | 2001 | A1 | *env* |
| AY288084 | KE | 1993 | A1 | *env* |
| AY322184 | KE | 1986 | A1 | *env* |
| AY463232 | ZA | 2000 | C | *env* |
| AY463235 | ZA | 2001 | C | *env* |
| AY494966 | UG | 1994 | D | *env* |
| AY522726 | ZA | 1999 | C | *env* |
| AY522736 | ZA | 1999 | C | *env* |
| AY623599 | UG | 1992 | D | *env* |
| AY669700 | RW | 1992 | A1 | *env* |
| AY669701 | UG | 1992 | A1 | *env* |
| AY669702 | RW | 1992 | A1 | *env* |
| AY669704 | UG | 1993 | A1 | *env* |
| AY669705 | UG | 1994 | A1 | *env* |
| AY669706 | RW | 1992 | A1 | *env* |
| AY669750 | UG | 1993 | D | *env* |
| AY669753 | UG | 1992 | D | *env* |
| AY669756 | UG | 1993 | D | *env* |
| AY669758 | UG | 1992 | D | *env* |
| AY669759 | UG | 1992 | D | *env* |
| AY669760 | UG | 1992 | D | *env* |
| AY713406 | RW | 1993 | A1 | *env* |
| AY713418 | UG | 1993 | D | *env* |
| AY736810 | KE | 2000 | A | *env* |
| AY775581 | TZ | 2000 | A | *env* |
| AY901974 | ZA | 2004 | C | *env* |
| AY945738 | KE | 1991 | C | *env* |
| DQ011173 | ZA | 2004 | C | *env* |
| DQ056412 | ZA | 2004 | C | *env* |
| DQ164129 | ZA | 2004 | C | *env* |
| DQ208444 | KE |  | A | *env* |
| DQ208447 | KE |  | A | *env* |
| DQ208449 | KE |  | A | *env* |
| DQ208465 | KE |  | A | *env* |
| DQ208492 | KE |  | A | *env* |
| DQ208499 | KE |  | A | *env* |
| DQ351228 | ZA | 2003 | C | *env* |
| EF151810 | UG | 1996 | D | *env* |
| EF151811 | UG | 1996 | A1 | *env* |
| EF203983 | ZA | 2005 | C | *env* |
| EF575384 | UG |  | D | *env* |
| EF575429 | UG |  | D | *env* |
| EF575444 | UG |  | D | *env* |
| EF583734 | UG |  | A1 | *env* |
| EF583735 | UG |  | A1 | *env* |
| EF583736 | UG |  | A1 | *env* |
| EF583737 | UG |  | A1 | *env* |
| EF583738 | UG |  | A1 | *env* |
| EF583739 | UG |  | A1 | *env* |
| EF583740 | UG |  | A1 | *env* |
| EF583741 | UG |  | A1 | *env* |
| EF583742 | UG |  | A1 | *env* |
| EF583743 | CM | 1999 | A1 | *env* |
| EF583781 | UG |  | D | *env* |
| EF583782 | UG |  | D | *env* |
| EF583783 | UG |  | D | *env* |
| EF583784 | UG |  | D | *env* |
| EF583785 | UG |  | D | *env* |
| EF583786 | UG |  | D | *env* |
| EF583788 | CM | 1996 | D | *env* |
| EF583790 | CM | 1998 | D | *env* |
| EU110088 | KE | 2001 | A1 | *env* |
| EU110093 | KE | 2002 | A1 | *env* |
| EU166681 | ZM | 2003 | C | *env* |
| EU166718 | ZM | 2003 | C | *env* |
| EU281995 | UG | 1997 | D | *env* |
| EU281998 | UG | 1997 | D | *env* |
| EU513183 | CM | 2005 | A1 | *env* |
| EU618630 | CM | 2002 | A1 | *env* |
| EU618647 | CM | 2003 | A1 | *env* |
| EU618649 | CM | 2003 | A1 | *env* |
| EU618651 | CM | 2003 | A1 | *env* |
| EU618652 | CM | 2003 | A1 | *env* |
| EU618659 | CM | 2003 | A1 | *env* |
| EU618669 | CM | 2004 | A1 | *env* |
| EU618683 | CM | 2003 | A1 | *env* |
| EU618692 | CM | 2003 | A1 | *env* |
| EU618696 | CM | 2004 | A1 | *env* |
| EU618697 | CM | 2004 | A1 | *env* |
| EU618698 | CM | 2004 | A1 | *env* |
| EU618714 | CM | 2003 | A1 | *env* |
| EU618724 | CM | 2004 | A1 | *env* |
| EU618738 | CM | 2004 | A1 | *env* |
| EU618759 | CM | 2002 | A1 | *env* |
| EU618781 | CM | 2002 | A1 | *env* |
| EU618791 | CM | 2002 | C | *env* |
| EU618811 | CM | 2003 | A1 | *env* |
| EU618837 | CM | 2004 | A1 | *env* |
| EU618879 | CM | 2003 | A1 | *env* |
| EU618894 | CM | 2004 | A1 | *env* |
| EU618897 | CM | 2002 | A1 | *env* |
| EU618909 | CM | 2004 | A1 | *env* |
| EU618972 | CM | 2001 | A1 | *env* |
| EU618973 | CM | 2001 | A1 | *env* |
| EU618974 | CM | 2001 | A1 | *env* |
| EU618975 | CM | 2001 | A1 | *env* |
| EU619000 | CM | 2001 | A1 | *env* |
| EU619013 | CM | 2001 | A1 | *env* |
| EU619041 | CM | 2001 | A1 | *env* |
| EU852934 | UG | 1997 | D | *env* |
| EU852942 | UG | 1997 | D | *env* |
| EU852950 | UG | 1998 | A1 | *env* |
| EU852966 | UG | 1997 | D | *env* |
| EU852974 | UG | 1997 | D | *env* |
| EU852990 | UG | 1997 | D | *env* |
| EU853014 | UG | 1997 | D | *env* |
| EU853022 | UG | 1997 | D | *env* |
| EU853030 | UG | 1999 | A1 | *env* |
| EU853038 | UG | 1999 | A1 | *env* |
| EU853046 | UG | 1997 | D | *env* |
| EU853054 | UG | 1997 | D | *env* |
| EU853062 | UG | 1997 | A1 | *env* |
| EU853070 | UG | 1997 | A1 | *env* |
| EU853086 | UG | 1998 | D | *env* |
| EU853094 | UG | 1998 | D | *env* |
| EU853102 | UG | 1998 | D | *env* |
| EU853110 | UG | 2001 | D | *env* |
| EU853118 | UG | 2001 | D | *env* |
| EU853126 | UG | 1997 | D | *env* |
| EU853134 | UG | 1997 | D | *env* |
| EU863448 | ZA | 2000 | C | *env* |
| EU863449 | ZA | 2000 | C | *env* |
| FJ346468 | KE | 2007 | D | *env* |
| FJ346472 | KE | 2007 | A1 | *env* |
| FJ346473 | KE | 2007 | D | *env* |
| FJ346476 | KE | 2007 | A1 | *env* |
| FJ346488 | KE | 2007 | D | *env* |
| FJ346489 | KE | 2007 | D | *env* |
| FJ346498 | KE | 2007 | A1 | *env* |
| FJ346503 | KE | 2007 | A1 | *env* |
| FJ346508 | KE | 2007 | A1 | *env* |
| FJ346528 | KE | 2007 | A1 | *env* |
| FJ396016 | KE | 1998 | A1 | *env* |
| FJ444077 | ZA | 2007 | C | *env* |
| FJ623480 | KE | 2006 | A1 | *env* |
| FJ623481 | KE | 2006 | A1 | *env* |
| FJ623484 | KE | 2006 | A1 | *env* |
| FJ623486 | KE | 2006 | A1 | *env* |
| FJ647148 | ZA | 2001 | A1 | *env* |
| FJ866136 | KE | 1993 | D | *env* |
| FJ866138 | KE | 1997 | D | *env* |
| GQ245681 | UG | 1996 | D | *env* |
| GU216837 | ZA |  | C | *env* |
| GU329053 | ZM | 2005 | C | *env* |
| GU329289 | ZM | 2005 | C | *env* |
| HM204620 | TZ | 2008 | A | *env* |
| HM204623 | TZ | 2008 | C | *env* |
| HM215267 | UG | 2007 | D | *env* |
| HM215269 | UG | 2007 | D | *env* |
| HM215270 | UG | 2006 | D | *env* |
| HM215271 | UG | 2007 | A | *env* |
| HM215275 | KE | 2007 | A | *env* |
| HM215327 | TZ | 2004 | D | *env* |
| HM215347 | TZ | 2002 | A | *env* |
| HQ615958 | ZA | 2005 | C | *env* |
| HQ615976 | TZ | 2008 | C | *env* |
| L07082 | RW |  | A | *env* |
| L22943 | KE | 1990 | A | *env* |
| L22948 | UG | 1990 | C | *env* |
| L22950 | UG | 1990 | D | *env* |
| M66533 | RW |  | A | *env* |
| U36867 | UG |  | D | *env* |
| U36884 | UG |  | D | *env* |
| U36886 | UG |  | D | *env* |
| U39239 | BI | 1991 | C | *env* |
| U39241 | BI | 1991 | C | *env* |
| U39245 | BI | 1991 | C | *env* |
| U46016 | ET | 1986 | C | *env* |
