# Supplementary material for: The Role of Viral Introductions in Sustaining Community-Based HIV Epidemics in Rural Uganda: Evidence from Spatial Clustering, Phylogenetics, and Egocentric Transmission Models
Source: PLoS Med. 2014 Mar 4;11(3):e1001610. doi: 10.1371/journal.pmed.1001610 (PMC3942316; doi:10.1371/journal.pmed.1001610)
Supplement: Table S2 — Summary of HIV sequences from 1,434 HIV-1-seropositive participants in RCCS R13. Table includes the HIV-1 group M subtype assignment of isolated viruses in gag and env genes. (DOCX) [file pmed.1001610.s015.docx]

| **Table S2. Summary of HIV sequences obtained from 1,434 HIV seropositive participants in RCCS R13.** | | | | | | | |
| --- | --- | --- | --- | --- | --- | --- | --- |
| ***env* Subtype (N=1026)** | ***gag* Subtype (N=915)** | | | | | |  |
|  |  | A | C | D | R* | NA** | Total |
|  | A | 243 | 6 | 100 | 8 | 80 | 437 |
|  | C | 2 | 20 | 10 | 3 | 3 | 38 |
|  | D | 30 | 5 | 401 | 5 | 101 | 542 |
|  | G | 1 | 0 | 0 | 0 | 0 | 1 |
|  | R* | 4 | 1 | 2 | 1 | 0 | 8 |
|  | NA** | 20 | 4 | 49 | 0 | 335 | 408 |
|  | Total | 300 | 36 | 562 | 17 | 519 | 1434*** |
| There were 1099 (77%) participants with sequence information in one or both genetic regions. Of those participants, 842 (59%) participants had viral sequence data for both *gag* and *env* genes and are highlighted in grey. *Recombinant viral sequence **Virus not amplifiable in *gag/env* gene region. ***Total number of ART naïve HIV-1 seropositive participants in study. | | | | | | | |
|  |  |  |  |  |  |  |  |
