# Supplementary material for: The Role of Viral Introductions in Sustaining Community-Based HIV Epidemics in Rural Uganda: Evidence from Spatial Clustering, Phylogenetics, and Egocentric Transmission Models
Source: PLoS Med. 2014 Mar 4;11(3):e1001610. doi: 10.1371/journal.pmed.1001610 (PMC3942316; doi:10.1371/journal.pmed.1001610)
Supplement: Table S3 — Summary of HIV sequences obtained from 189 HIV-1-incident participants in RCCS R13. Table includes the HIV-1 group M subtype assignment of isolated viruses in gag and env genes. (DOCX) [file pmed.1001610.s016.docx]

| **Table S3. Summary of HIV sequences obtained from 189 HIV-1 incident participants in RCCS R13** | | | | | | | |
| --- | --- | --- | --- | --- | --- | --- | --- |
| ***env* Subtype (N=153)** | ***gag* Subtype (N=139)** | | | | | |  |
|  |  | A | C | D | R* | NA** | Total |
|  | A | 45 | 0 | 15 | 2 | 15 | 77 |
|  | C | 0 | 2 | 2 | 0 | 0 | 4 |
|  | D | 6 | 0 | 52 | 1 | 10 | 69 |
|  | R* | 1 | 0 | 2 | 0 | 0 | 3 |
|  | NA** | 3 | 0 | 8 | 0 | 24 | 35 |
|  | Total | 55 | 2 | 79 | 3 | 49 | 188*** |
| There were 164 (87%) participants with sequence information in one or both genetic regions. Of those participants, 128 (68%) participants had viral sequence data for both *gag* and *env* genes and are highlighted in grey. *Recombinant viral sequence. **Virus not amplifiable in *gag/env* gene region. ***Total number of ART naïve HIV-1 incident cases in study. One participant was on ART at the time of study visit and was excluded from the viral sequence analysis. | | | | | | | |
|  |  |  |  |  |  |  |  |
|  |  |  |  |  |  |  |  |
|  |  |  |  |  |  |  |  |
|  |  |  |  |  |  |  |  |
