# Supplementary material for: The Role of Viral Introductions in Sustaining Community-Based HIV Epidemics in Rural Uganda: Evidence from Spatial Clustering, Phylogenetics, and Egocentric Transmission Models
Source: PLoS Med. 2014 Mar 4;11(3):e1001610. doi: 10.1371/journal.pmed.1001610 (PMC3942316; doi:10.1371/journal.pmed.1001610)
Supplement: Table S4 — Summary phylogenetic data (HIV subtype, genetic pairwise distance, and phylogenetic clustering results) for the 105 epidemiologically linked incident couples with phylogenetic data in gag and/or env gene regions. (DOCX) [file pmed.1001610.s017.docx]

| **Table S4**. **Summary phylogenetic data (HIV subtype, genetic pairwise distance, and phylogenetic clustering) for the 105 epidemiologically linked incident couples with phylogenetic data in either or both the *gag* and *env* gene regions**. | | | | | | |
| --- | --- | --- | --- | --- | --- | --- |
| HIV-1 serostatus (Partner 1:Partner 2) | HIV-1 *gag* subtype (Partner 1:Partner 2) | *gag* genetic distance | Phylogenetically clusters in *gag* | HIV-1 *env* subtype (Partner 1:Partner 2) | *env* genetic distance | Phylogenetically clusters in *env* |
| Incident:Incident | D:D | 0.0000 | yes | A:C | 0.0910 | no |
| Incident:Incident | A:A | 0.0000 | yes | A:A | 0.0086 | no |
| Incident:Incident | A:A | 0.0089 | yes | A:A | 0.0000 | yes |
| Incident:Incident | A:A | 0.0036 | yes | A:A | 0.0000 | yes |
| Incident:Incident | D:D | 0.0036 | yes | D:D | 0.0043 | yes |
| Incident:Incident | A:A | 0.0018 | yes | A:A | 0.0108 | yes |
| Incident:Incident | - | - | - | A:A | 0.0259 | no |
| Incident:Prevalent | D:D | 0.0036 | yes | - | - | - |
| Incident:Prevalent | A:A | 0.0053 | no | A:A | 0.0045 | yes |
| Incident:Prevalent | D:D | 0.0036 | yes | A:A | 0.0095 | yes |
| Incident:Prevalent | A:A | 0.0053 | yes | - | - | - |
| Incident:Prevalent | D:D | 0.0018 | yes | D:D | 0.0043 | yes |
| Incident:Prevalent | D:D | 0.0000 | yes | D:D | 0.0043 | yes |
| Incident:Prevalent | A:A | 0.0036 | yes | A:A | 0.0086 | yes |
| Incident:Prevalent | A:A | 0.0036 | yes | - | - | - |
| Incident:Prevalent | D:D | 0.0036 | yes | C:C | 0.0065 | yes |
| Incident:Prevalent | - | - | - | A:A | 0.0918 | no |
| Incident:Prevalent | - | - | - | D:D | 0.0510 | no |
| Incident:Prevalent | - | - | - | D:D | 0.0108 | yes |
| Incident:Prevalent | - | - | - | A:A | 0.0195 | yes |
| Prevalent:Incident | A:A | 0.0838 | no | A:A | 0.1240 | no |
| Prevalent:Incident | A:A | 0.0053 | no | A:A | 0.0086 | no |
| Prevalent:Incident | A:A | 0.0515 | no | A:A | 0.0130 | yes |
| Prevalent:Incident | D:D | 0.0107 | yes | D:D | 0.0426 | no |
| Prevalent:Incident | D:D | 0.0018 | yes | A:A | 0.0094 | yes |
| Prevalent:Incident | D:D | 0.0558 | no | A:D | 0.0653 | no |
| Prevalent:Incident | D:D | 0.0036 | yes | A:A | 0.0000 | yes |
| Prevalent:Incident | D:D | 0.0126 | no | D:D | 0.0043 | no |
| Prevalent:Incident | D:D | 0.0144 | no | D:D | 0.0086 | yes |
| Prevalent:Incident | - | - | - | D:D | 0.0131 | no |
| Prevalent:Incident | - | - | - | D:D | 0.0086 | no |
| Prevalent:Incident | - | - | - | A:A | 0.0086 | yes |
| Prevalent:Prevalent | A:A | 0.0053 | yes | - | - | - |
| Prevalent:Prevalent | A:A | 0.0090 | no | A:A | 0.0197 | no |
| Prevalent:Prevalent | D:D | 0.0309 | no | D:D | 0.0263 | no |
| Prevalent:Prevalent | A:A | 0.0126 | yes | A:A | 0.0173 | yes |
| Prevalent:Prevalent | A:A | 0.0234 | no | A:A | 0.0265 | no |
| Prevalent:Prevalent | D:D | 0.0617 | no | - | - | - |
| Prevalent:Prevalent | A:C | 0.1467 | no | A:C | 0.1475 | no |
| Prevalent:Prevalent | A:A | 0.0107 | yes | A:A | 0.0307 | no |
| Prevalent:Prevalent | D:D | 0.0198 | no | - | - | - |
| Prevalent:Prevalent | D:D | 0.0217 | no | D:D | 0.0132 | no |
| Prevalent:Prevalent | D:D | 0.0144 | no | A:A | 0.0262 | no |
| Prevalent:Prevalent | D:D | 0.0071 | yes | D:D | 0.0199 | no |
| Prevalent:Prevalent | D:D | 0.0089 | yes | D:D | 0.0086 | yes |
| Prevalent:Prevalent | D:D | 0.0071 | no | D:D | 0.0152 | yes |
| Prevalent:Prevalent | A:A | 0.0272 | no | A:A | 0.0362 | no |
| Prevalent:Prevalent | A:A | 0.0036 | yes | - | - | - |
| Prevalent:Prevalent | D:D | 0.0126 | no | D:D | 0.0353 | no |
| Prevalent:Prevalent | D:D | 0.0126 | yes | D:D | 0.1480 | no |
| Prevalent:Prevalent | A:D | 0.1106 | no | A:A | 0.0720 | no |
| Prevalent:Prevalent | D:D | 0.0145 | no | - | - | - |
| Prevalent:Prevalent | D:D | 0.0161 | no | D:D | 0.0086 | yes |
| Prevalent:Prevalent | D:D | 0.0071 | yes | D:D | 0.0444 | no |
| Prevalent:Prevalent | D:D | 0.0107 | yes | D:D | 0.0195 | yes |
| Prevalent:Prevalent | D:D | 0.0235 | no | D:D | 0.0421 | no |
| Prevalent:Prevalent | A:A | 0.0036 | yes | A:A | 0.0262 | no |
| Prevalent:Prevalent | D:D | 0.0347 | no | - | - | - |
| Prevalent:Prevalent | D:D | 0.0463 | no | D:D | 0.0556 | no |
| Prevalent:Prevalent | C:C | 0.0144 | no | D:D | 0.0108 | yes |
| Prevalent:Prevalent | A:D | 0.1174 | no | A:D | 0.1344 | no |
| Prevalent:Prevalent | D:D | 0.0089 | yes | D:D | 0.0306 | no |
| Prevalent:Prevalent | A:A | 0.0125 | yes | A:A | 0.0306 | no |
| Prevalent:Prevalent | A:A | 0.0071 | yes | A:A | 0.0086 | yes |
| Prevalent:Prevalent | D:D | 0.0054 | yes | A:A | 0.0195 | yes |
| Prevalent:Prevalent | D:D | 0.0018 | yes | D:D | 0.0087 | yes |
| Prevalent:Prevalent | A:D | 0.1207 | no | A:A | 0.0825 | no |
| Prevalent:Prevalent | D:D | 0.0108 | no | - | - | - |
| Prevalent:Prevalent | A:A | 0.0125 | yes | A:A | 0.0177 | yes |
| Prevalent:Prevalent | D:D | 0.0018 | yes | D:D | 0.0043 | yes |
| Prevalent:Prevalent | A:D | 0.1083 | no | A:D | 0.1455 | no |
| Prevalent:Prevalent | D:D | 0.0089 | yes | D:D | 0.0218 | yes |
| Prevalent:Prevalent | A:C | 0.1112 | no | A:C | 0.1404 | no |
| Prevalent:Prevalent | D:D | 0.0273 | no | D:D | 0.0351 | no |
| Prevalent:Prevalent | D:D | 0.0125 | yes | D:D | 0.0262 | no |
| Prevalent:Prevalent | A:A | 0.0772 | no | A:A | 0.0679 | no |
| Prevalent:Prevalent | D:D | 0.0143 | no | D:D | 0.0418 | no |
| Prevalent:Prevalent | D:D | 0.0216 | no | - | - | - |
| Prevalent:Prevalent | D:D | 0.0180 | no | - | - | - |
| Prevalent:Prevalent | D:D | 0.0253 | no | - | - | - |
| Prevalent:Prevalent | D:D | 0.0107 | yes | D:D | 0.0306 | no |
| Prevalent:Prevalent | A:A | 0.0216 | no | A:A | 0.0151 | yes |
| Prevalent:Prevalent | D:D | 0.0180 | no | D:D | 0.0220 | no |
| Prevalent:Prevalent | A:D | 0.1165 | no | - | - | - |
| Prevalent:Prevalent | A:D | 0.1047 | no | A:A | 0.0768 | no |
| Prevalent:Prevalent | A:A | 0.0615 | no | A:A | 0.0899 | no |
| Prevalent:Prevalent | A:A | 0.0089 | yes | A:A | 0.0198 | yes |
| Prevalent:Prevalent | - | - | - | D:D | 0.0533 | no |
| Prevalent:Prevalent | - | - | - | D:D | 0.0329 | no |
| Prevalent:Prevalent | - | - | - | D:D | 0.0129 | yes |
| Prevalent:Prevalent | - | - | - | D:D | 0.0374 | no |
| Prevalent:Prevalent | - | - | - | D:D | 0.0217 | yes |
| Prevalent:Prevalent | - | - | - | A:A | 0.0329 | no |
| Prevalent:Prevalent | - | - | - | A:A | 0.0261 | no |
| Prevalent:Prevalent | - | - | - | A:A | 0.0400 | no |
| Prevalent:Prevalent | - | - | - | D:D | 0.0108 | yes |
| Prevalent:Prevalent | - | - | - | C:D | 0.1825 | no |
| Prevalent:Prevalent | - | - | - | A:D | 0.1186 | no |
| Prevalent:Prevalent | - | - | - | A:D | 0.1799 | no |
| Prevalent:Prevalent | - | - | - | D:D | 0.0261 | no |
| Prevalent:Prevalent | - | - | - | A:A | 0.0327 | no |
| Prevalent:Prevalent | - | - | - | D:D | 0.0965 | no |
| Prevalent:Prevalent | - | - | - | D:D | 0.1082 | no |
| Prevalent:Prevalent | - | - | - | A:A | 0.0602 | no |
| Prevalent:Prevalent | - | - | - | A:A | 0.0535 | no |
