# Supplementary material for: The Role of Viral Introductions in Sustaining Community-Based HIV Epidemics in Rural Uganda: Evidence from Spatial Clustering, Phylogenetics, and Egocentric Transmission Models
Source: PLoS Med. 2014 Mar 4;11(3):e1001610. doi: 10.1371/journal.pmed.1001610 (PMC3942316; doi:10.1371/journal.pmed.1001610)
Supplement: Table S5 — Detailed summary data for each of the 95 phylogenetic clusters identified in maximum likelihood phylogenetic trees (HKY-85 model). (DOCX) [file pmed.1001610.s018.docx]

| **Table S5**. Composition of 95 phylogenetic clusters identified in maximum likelihood phylogenetic analyses (HKY-85) of 915 *gag* sequences and 1026 *env* sequences obtained from 1,099 HIV-infected participants in RCCS R13 | | | | | | | | |
| --- | --- | --- | --- | --- | --- | --- | --- | --- |
| Cluster ID | HIV-1 *gag* subtype | HIV-1 *env* subtype | Total Participants | Incident cases | Prevalent Cases | Households | Communities | Geographic Regions |
| 1 | A | A | 2 | 1 | 1 | 2 | 2 | 1 |
| 2 | A | - | 2 | 0 | 2 | 1 | 1 | 1 |
| 3 | A | A | 2 | 1 | 1 | 2 | 2 | 1 |
| 4 | A | - | 2 | 0 | 2 | 2 | 1 | 1 |
| 5 | A | A | 3 | 0 | 3 | 3 | 3 | 1 |
| 6 | A | - | 2 | 1 | 1 | 1 | 1 | 1 |
| 7 | A | A | 2 | 0 | 2 | 1 | 1 | 1 |
| 8 | A | A | 4 | 4 | 0 | 2 | 2 | 2 |
| 9 | A | A | 2 | 2 | 0 | 1 | 1 | 1 |
| 10 | A | A | 2 | 0 | 2 | 2 | 1 | 1 |
| 11 | A | - | 2 | 1 | 1 | 2 | 2 | 1 |
| 12 | A | A | 2 | 0 | 2 | 2 | 1 | 1 |
| 13 | A | - | 3 | 1 | 2 | 3 | 3 | 2 |
| 14 | A | A | 3 | 1 | 2 | 2 | 2 | 1 |
| 15 | A | - | 2 | 0 | 2 | 1 | 1 | 1 |
| 16 | A | A | 5 | 4 | 1 | 3 | 2 | 2 |
| 17 | A | D | 2 | 0 | 2 | 2 | 2 | 2 |
| 18 | A | - | 2 | 1 | 1 | 1 | 1 | 1 |
| 19 | A | - | 2 | 0 | 2 | 1 | 1 | 1 |
| 20 | A | - | 2 | 0 | 2 | 1 | 1 | 1 |
| 21 | A | A | 5 | 2 | 3 | 3 | 1 | 1 |
| 22 | A | - | 2 | 0 | 2 | 2 | 1 | 1 |
| 23 | A | A | 2 | 0 | 2 | 1 | 1 | 1 |
| 24 | A | - | 2 | 0 | 2 | 2 | 2 | 1 |
| 25 | A | A | 2 | 0 | 2 | 1 | 1 | 1 |
| 26 | A | A | 2 | 0 | 2 | 1 | 1 | 1 |
| 27 | A | A | 2 | 0 | 2 | 2 | 2 | 1 |
| 28 | D | - | 2 | 0 | 2 | 2 | 2 | 2 |
| 29 | D | D | 2 | 0 | 2 | 2 | 2 | 1 |
| 30 | D | - | 2 | 1 | 1 | 1 | 1 | 1 |
| 31 | D | - | 2 | 2 | 0 | 1 | 1 | 1 |
| 32 | D | A | 2 | 1 | 1 | 1 | 1 | 1 |
| 33 | D | A | 4 | 0 | 4 | 3 | 3 | 2 |
| 34 | D | A | 2 | 1 | 1 | 1 | 1 | 1 |
| 35 | D | - | 2 | 1 | 1 | 1 | 1 | 1 |
| 36 | D | - | 2 | 0 | 2 | 1 | 1 | 1 |
| 37 | D | D | 2 | 0 | 2 | 1 | 1 | 1 |
| 38 | D | - | 2 | 1 | 1 | 2 | 2 | 1 |
| 39 | D | D | 2 | 0 | 2 | 1 | 1 | 1 |
| 40 | D | D | 2 | 0 | 2 | 2 | 2 | 2 |
| 41 | D | - | 2 | 0 | 2 | 1 | 1 | 1 |
| 42 | D | D | 3 | 0 | 3 | 2 | 2 | 2 |
| 43 | D | - | 2 | 0 | 2 | 1 | 1 | 1 |
| 44 | D | D | 2 | 0 | 2 | 1 | 1 | 1 |
| 45 | D | A | 2 | 1 | 1 | 1 | 1 | 1 |
| 46 | D | A | 3 | 1 | 2 | 3 | 1 | 1 |
| 47 | D | A | 2 | 0 | 2 | 2 | 2 | 1 |
| 48 | D | - | 2 | 0 | 2 | 2 | 2 | 2 |
| 49 | D | - | 2 | 0 | 2 | 1 | 1 | 1 |
| 50 | D | D | 2 | 0 | 2 | 2 | 2 | 1 |
| 51 | D | D | 2 | 1 | 1 | 2 | 1 | 1 |
| 52 | D | A | 2 | 0 | 2 | 2 | 2 | 1 |
| 53 | D | C | 2 | 1 | 1 | 1 | 1 | 1 |
| 54 | D | D | 2 | 0 | 2 | 2 | 2 | 1 |
| 55 | D | D | 2 | 1 | 1 | 1 | 1 | 1 |
| 56 | D | - | 2 | 1 | 1 | 2 | 2 | 2 |
| 57 | D | - | 2 | 0 | 2 | 1 | 1 | 1 |
| 58 | D | D | 2 | 2 | 0 | 1 | 1 | 1 |
| 59 | D | D | 3 | 0 | 3 | 2 | 2 | 2 |
| 60 | D | D | 2 | 1 | 1 | 1 | 1 | 1 |
| 61 | D | D | 2 | 2 | 0 | 2 | 2 | 2 |
| 62 | D | - | 2 | 0 | 2 | 1 | 1 | 1 |
| 63 | D | - | 3 | 1 | 2 | 3 | 2 | 1 |
| 64 | C | - | 2 | 0 | 2 | 2 | 2 | 2 |
| 65 | C | C | 3 | 1 | 2 | 3 | 1 | 1 |
| 66 | - | A | 2 | 0 | 2 | 2 | 2 | 2 |
| 67 | - | A | 2 | 1 | 1 | 1 | 1 | 1 |
| 68 | - | A | 3 | 0 | 3 | 3 | 3 | 2 |
| 69 | - | A | 2 | 1 | 1 | 1 | 1 | 1 |
| 70 | - | A | 2 | 1 | 1 | 2 | 2 | 2 |
| 71 | - | A | 2 | 0 | 2 | 2 | 2 | 2 |
| 72 | - | A | 2 | 0 | 2 | 1 | 1 | 1 |
| 73 | - | A | 2 | 1 | 1 | 1 | 1 | 1 |
| 74 | - | A | 2 | 0 | 2 | 2 | 2 | 1 |
| 75 | - | A | 2 | 1 | 1 | 2 | 1 | 1 |
| 76 | - | A | 2 | 0 | 2 | 2 | 1 | 1 |
| 77 | - | A | 2 | 0 | 2 | 2 | 2 | 1 |
| 78 | - | A | 2 | 0 | 2 | 2 | 2 | 1 |
| 79 | - | D | 2 | 0 | 2 | 2 | 2 | 1 |
| 80 | - | D | 2 | 0 | 2 | 1 | 1 | 1 |
| 81 | - | D | 2 | 1 | 1 | 2 | 1 | 1 |
| 82 | - | D | 2 | 0 | 2 | 2 | 2 | 2 |
| 83 | - | D | 2 | 0 | 2 | 1 | 1 | 1 |
| 84 | - | D | 2 | 0 | 2 | 1 | 1 | 1 |
| 85 | - | D | 2 | 1 | 1 | 1 | 1 | 1 |
| 86 | - | D | 2 | 1 | 1 | 1 | 1 | 1 |
| 87 | - | D | 2 | 0 | 2 | 2 | 1 | 1 |
| 88 | - | D | 2 | 0 | 2 | 1 | 1 | 1 |
| 89 | - | D | 2 | 0 | 2 | 1 | 1 | 1 |
| 90 | - | D | 2 | 0 | 2 | 1 | 1 | 1 |
| 91 | - | D | 2 | 0 | 2 | 2 | 2 | 1 |
| 92 | - | D | 2 | 0 | 2 | 2 | 1 | 1 |
| 93 | - | D | 2 | 2 | 0 | 2 | 1 | 1 |
| 94 | - | C | 2 | 1 | 1 | 2 | 2 | 1 |
| 95 | - | C | 2 | 0 | 2 | 2 | 1 | 1 |
