# Supplementary material for: The Role of Viral Introductions in Sustaining Community-Based HIV Epidemics in Rural Uganda: Evidence from Spatial Clustering, Phylogenetics, and Egocentric Transmission Models
Source: PLoS Med. 2014 Mar 4;11(3):e1001610. doi: 10.1371/journal.pmed.1001610 (PMC3942316; doi:10.1371/journal.pmed.1001610)
Supplement: Table S6 — Sensitivity analyses of phylogenetic clustering results to choice of evolutionary model and bootstrap and genetic distance thresholds. Phylogenetic cluster analyses were conducted at 70%, 80%, 90%, and 99% bootstrap thresholds, with and without genetic distance cutoffs under the HKY-85 and GTR+I+G models of evolution. We present the cluster summary data shown in Table 2 under these different evolutionary models and genetic distance and bootstrap threshold criteria. (DOCX) [file pmed.1001610.s019.docx]

| **Table S6. Sensitivity analyses of phylogenetic clustering results to the evolutionary model and bootstrap and genetic distance thresholds.** | | | | | | | | |
| --- | --- | --- | --- | --- | --- | --- | --- | --- |
|  | | | Total clusters | Cluster size distribution | Clusters containing only incident cases | Household clusters^a^ | Intra-community clusters^b^ | Cross community clusters^c^ |
| Model of nucleotide evolution | Bootstrap threshold (%) | Genetic distance threshold | No. | No. of Participants in cluster (frequency) | No. of Clusters (% of clusters) | No. of Clusters (% of clusters) | No. of Clusters (% of clusters) | No. of Clusters (% of clusters) |
| HKY-85 | 99 | yes | 39 | 2 (34), 3 (4), 5 (1) | 4 (10.2) | 12 (30.1) | 9 (23.0) | 18 (46.1) |
|  | 90 | yes | 95 | 2 (82), 3 (9), 4 (2), 5 (2) | 6 (6.3) | 42 (44.2) | 15 (15.8) | 38 (40.0) |
|  | 80 | yes | 109 | 2 (94), 3 (10), 4 (3), 5(2) | 5 (4.6) | 48 (44.0) | 18 (16.5) | 43 (39.4) |
|  | 70 | yes | 112 | 2 (97), 3 (10), 4 (3), 5 (2) | 5 (4.4) | 49 (43.8) | 19 (17.0) | 44 (39.2) |
| HKY-85 | 99 | no | 64 | 2 (54), 3 (7), 4 (1), 5 (1), 6 (1) | 5 (7.8) | 24 (37.5) | 9 (14.0) | 31 (48.4) |
|  | 90 | no | 108 | 2 (84), 3 (16), 4 (4), 5 (3), 6 (1) | 6 (5.6) | 42 (38.9) | 17 (15.7) | 49 (45.3) |
|  | 80 | no | 137 | 2 (107), 3 (22), 4 (4), 5 (3), 7 (1) | 6 (4.8) | 50 (36.5) | 25 (18.2) | 62 (45.2) |
|  | 70 | no | 158 | 2 (122), 3 (27), 4 (4), 5 (3), 7 (1), 9 (1) | 6 (3.7) | 54 (34.1) | 27 (27.0) | 77 (48.0) |
| GTR+I+G | 99 | yes | 39 | 2 (34), 3 (4), 5 (1) | 4 (10.2) | 13 (33.3) | 8 (20.5) | 18 (46.1) |
|  | 90 | yes | 92 | 2 (80), 3 (8), 4 (2), 5 (1) | 6 (6.5) | 40 (44.6) | 14 (15.2) | 37 (40.2) |
|  | 80 | yes | 105 | 2 (91), 3 (9), 4 (3), 5 (2) | 5 (4.7) | 48 (45.8) | 17 (16.2) | 40 (38.1) |
|  | 70 | yes | 109 | 2 (95), 3 (9), 4 (3), 5 (2) | 5 (4.6) | 49 (45.0) | 18 (16.5) | 42 (38.5) |
| GTR+I+G | 99 | no | 40 | 2 (35), 3 (4), 5 (1) | 4 (10.0) | 13 (32.5) | 8 (20.0) | 19 (47.5) |
|  | 90 | no | 103 | 2 (82), 3 (14), 4 (4), 5 (2), 6 (1) | 6 (5.8) | 40 (38.8) | 16 (15.5) | 47 (45.6) |
|  | 80 | no | 134 | 2 (104), 3 (21), 4 (5), 5 (3), 7 (1) | 5 (3.7) | 51 (38.1) | 23 (17.2) | 60 (44.8) |
|  | 70 | no | 150 | 2 (113), 3 (27), 4 (5), 5 (4), 7 (1) | 6 (4.0) | 53 (35.3) | 26 (17.3) | 71 (47.3) |
| ^a^ Refers to clusters of two individuals who share the same household ^b^ Refers to clusters of two more individuals who spanned households but shared the same community ^c^ Refers to clusters of two or more individuals who spanned households and communities | | | | | | | | |
