# Supplementary material for: The Role of Viral Introductions in Sustaining Community-Based HIV Epidemics in Rural Uganda: Evidence from Spatial Clustering, Phylogenetics, and Egocentric Transmission Models
Source: PLoS Med. 2014 Mar 4;11(3):e1001610. doi: 10.1371/journal.pmed.1001610 (PMC3942316; doi:10.1371/journal.pmed.1001610)
Supplement: Table S7 — Numbers of recent sexual partners self-reported by 9,520 HIV-seronegative and -incident participants in egocentric analysis by gender and marital status of the study participant. (DOCX) [file pmed.1001610.s020.docx]

| **Table S7. Numbers of recent sexual partners self-reported by 9,520 HIV seronegative and incident participants in egocentric analysis by gender and marital status of the study participant.** | | | | | |
| --- | --- | --- | --- | --- | --- |
|  | **Females (n=5368)** | | | |  |
|  | Unmarried-never married | Unmarried-previously married | Married - not polygamous | Married - polygamous* | Total |
|  | N (%) | N (%) | N (%) | N (%) | N (%) |
| 1 partner | 573 (94.1) | 755 (92.9) | 2893 (97.3) | 938 (96.5) | 5159 (96.1) |
| 2 partners | 34 (5.6) | 53 (6.5) | 79 (2.7) | 31 (3.2) | 197 (3.7) |
| 3 partners | 2 (0.3) | 5 (6.1) | 2 (0.0) | 3 (0.3) | 12 (0.2) |
| 4 partners | 0 (0.0) | 0 (0.0) | 0 (0.0) | 0 (0.0) | 0 |
| Total | 0 (11.3) | 813 (15.1) | 2974 (55.4) | 972 (18.1) | 5368 |
|  | **Males (n=5162)** | | | |  |
|  | Unmarried-never married | Unmarried-previously married | Married - not polygamous | Married - polygamous | Total |
|  | N (%) | N (%) | N (%) | N (%) | N (%) |
| 1 partner | 655 (71.4) | 211 (64.9) | 1582 (64.2) | 10 (2.2) | 2458 (59.2) |
| 2 partners | 175 (19.1) | 68 (20.9) | 669 (27.2) | 283 (63.3) | 1195 (28.8) |
| 3 partners | 77 (8.4) | 39 (12.0) | 192 (7.8) | 135 (29.8) | 441 (10.6) |
| 4 partners | 10 (1.1) | 7 (2.1) | 20 (0.8) | 21 (4.7) | 58 (1.4) |
| Total | 917 (22.1) | 325 (78.2) | 2463 (59.3) | 447 (10.8) | 4152 |
| * Married - polygamous for females refers to a female in a marital relationship with a man that has multiple wives. | | | | | |
