# Supplementary material for: The Role of Viral Introductions in Sustaining Community-Based HIV Epidemics in Rural Uganda: Evidence from Spatial Clustering, Phylogenetics, and Egocentric Transmission Models
Source: PLoS Med. 2014 Mar 4;11(3):e1001610. doi: 10.1371/journal.pmed.1001610 (PMC3942316; doi:10.1371/journal.pmed.1001610)
Supplement: Table S8 — Summary of self-reported sexual partner data from 9,520 HIV-seronegative and -incident participants in egocentric analysis by gender of the study participant and geographic location of the sexual partner. (DOCX) [file pmed.1001610.s021.docx]

| **Table S8. Summary of self-reported sexual partner data from 9,520 HIV seronegative and incident participants in egocentric analysis by gender of the study participant and geographic location of the sexual partner.** | | | | | | | | | |
| --- | --- | --- | --- | --- | --- | --- | --- | --- | --- |
| **Sexual partners of known HIV serostatus (N=5043)*** | | | | | | | | | |
|  | Female | | | |  | Male | | | |
|  | Household | Community | Extra-community | Total |  | Household | Community | Extra-community | Total |
|  | N (%) | N (%) | N (%) | N (%) |  | N (%) | N (%) | N (%) | N (%) |
| Stable | 2346 (99.9) | 82 (97.6) | 11(91.2) | 2439 (99.8) | Stable | 2483 (99.9) | 87 (85.2) | 11 (91.2) | 2581 (99.3) |
| Unstable | 1 (0.1) | 2 (2.3) | 1 (8.8) | 4 (0.2) | Unstable | 3 (0.1) | 15 (14.8) | 1 (8.8) | 19 (0.7) |
| Total | 2347 (96.1) | 84 (3.4) | 12 (0.5) | 2443 | Total | 2486 (95.6) | 102 (3.9) | 12 (0.5) | 2600 |
| **Sexual partners of unknown HIV serostatus (N=6949)** | | | | | | | | | |
|  | Female | | | |  | Male | | | |
|  | Household | Community | Extra-community | Total |  | Household | Community | Extra-community | Total |
|  | N (%) | N (%) | N (%) | N (%) |  | N (%) | N (%) | N (%) | N (%) |
| Stable | 1729 (99.2) | 2 (0.3) | 12 (1.6) | 1743 (55.4) | Stable | 959 (98.7) | 12 (0.8) | 9 (0.7) | 980 (25.8) |
| Unstable | 15 (0.8) | 616 (99.7) | 772 (98.4) | 1403 (44.6) | Unstable | 13 (1.3) | 1526 (99.2) | 1284 (99.3) | 2823 (74.2) |
| Total | 1744 (55.8) | 618 (19.5) | 784 (24.7) | 3146 | Total | 972 (25.7) | 1538 (40.4) | 1293 (33.9) | 3803 |
| *83 incident cases occurred among individuals in which the HIV-status of at least one of their reported sexual partners was known. | | | | | | | | | |
