# Supplementary material for: The Role of Viral Introductions in Sustaining Community-Based HIV Epidemics in Rural Uganda: Evidence from Spatial Clustering, Phylogenetics, and Egocentric Transmission Models
Source: PLoS Med. 2014 Mar 4;11(3):e1001610. doi: 10.1371/journal.pmed.1001610 (PMC3942316; doi:10.1371/journal.pmed.1001610)
Supplement: Table S9 — Comparison of demographics and sexual behaviors (percent distribution) between RCCS study population (RCCS R13, 2008–2009) and the surveyed population in the 2011 Ugandan Demographic and Health Survey. (DOCX) [file pmed.1001610.s022.docx]

| **Table S9. Comparison of demographics and sexual behaviors (% distribution) between the RCCS study population (RCCS R13, 2008-2009) and the surveyed population in the 2011 Ugandan Demographic and Health Survey (DHS)** | | | | | | |
| --- | --- | --- | --- | --- | --- | --- |
|  | **RCCS R13, 2008-2009** | | **Ugandan DHS, 2011** | | **Ugandan DHS, 2011** | |
|  |  |  | **Central Region 1*** | | **All of Uganda** | |
|  | **Females** | **Males** | **Females** | **Males** | **Females** | **Males** |
|  | N=8188 | N=6406 | N=767 | N=188 | n=8674 | N=2295 |
|  | % (95% CI) | % (95% CI) | Weighted % | Weighted % | Weighted % | Weighted % |
| **Age distribution (15-49 years old)** |  |  |  |  |  |  |
| 15-19 | 17.4 (16.6-18.2) | 21.8 (20.7-22.8) | 24.0 (19.5-29.3) | 21.7 (16.2-28.3) | 23.6 (22.6-24.7) | 25.5 (23.3-27.7) |
| 20-24 | 17.5 (16.7-18.4) | 15.7 (14.8-16.6) | 17.8 (14.9-21.1) | 13.6 (9.2-19.6) | 18.9 (17.9-19.7) | 14.7 (13.0-16.5) |
| 25-29 | 20.6 (19.7-21.4) | 16.8 (15.9-17.8) | 16.3 (13.1-20.0) | 24.0 (16.7-33.1) | 18.1 (17.1-19.2) | 16.6 (14.8-18.6) |
| 30-34 | 17.9 (17.0-18.7) | 16.2 (15.3-17.2) | 13.5 (10.8-16.9) | 15.3 (10.7-21.5) | 12.5 (11.7-13.4) | 14.9 (13.0-17.0) |
| 35-39 | 11.7 (11.0-12.4) | 13.7 (12.9-14.6) | 11.1 (8.7-14.2) | 9.5 (5.6-15.8) | 11.8 (11.1-12.6) | 12.3 (10.9-14.0) |
| 40-44 | 7.8 (7.3-8.5) | 9.3 (8.6-10.0) | 7.9 (6.1-10.2) | 7.5(4.3-12.9) | 8.4 (7.7-9.2) | 8.8 (7.5-10.3) |
| 45-49 | 7.1 (6.5-7.6) | 6.4 (5.8-7.0) | 9.4 (7.7-11.4) | 8.4 (4.4-15.4) | 6.7 (6.2-7.4) | 7.2 (6.1-8.6) |
| **Education** |  |  |  |  |  |  |
| No education | 6.2 (5.7-6.8) | 3.4 (3.0-3.9) | 9.2 (6.5-12.9) | 5.6 (2.6-11.7) | 12.9 (11.6-14.3) | 4.5 (3.6-5.8) |
| Primary | 61.5 (60.4-62.6) | 65.7 (64.5-66.9) | 54.4 (48.7-59.9) | 65.0 (57.8-71.7) | 59.4 (57.5-61.3) | 60.0 (57.3-63.0) |
| Secondary | 26.7 (25.7-27.7) | 24.7 (23.6-25.8) | 30.5 (25.4-36.3) | 22.7 (17.4-29.1) | 22.5 (20.8-24.2) | 26.9 (24.4-29.5) |
| Higher than secondary | 5.6 (5.1-6.1) | 6.2 (5.6-6.8) | 5.9 (3.8-9.1) | 6.7 (3.6-12.4) | 5.2 (4.5-6.1) | 8.4 (7.1-9.9) |
| **Currently married or in cohabitating union** | 62.7 (61.6-63.8) | 57.3 (0.56-0.59) | 58.5 (53.8-63.1) | 60.1 (53.4-66.5) | 62.5 (61.0-63.9) | 58.3 (55.6-60.9) |
| **Polygyny (married men)** | - | 15.2 (14.3-16.1) | - | 16.1 (10.2-25.6) | - | 16.8 (14.7-19.2) |
| **No. of sex partners in the last year** |  |  |  |  |  |  |
| None | 18.0 (17.2-18.9) | 21.4 (20.4-22.4) | 26.1 (22.6-30.0) | 25.5 (19.5-32.6) | 27.3 (26.1-28.6) | 26.7 (24.5-29.1) |
| 1 | 77.3 (76.4-78.2) | 44.3 (43.1-45.5) | 70.4 (66.8-73.7) | 46.0 (39.6-52.3) | 71.0 (69.7-72.2) | 53.9 (51.3-56.5) |
| 2 | 4.2 (3.8-4.7) | 24.0 (22.9-25.0) | 3.1 (1.9-5.0) | 23.7 (18.4-29.9) | 1.6 (1.3-1.9) | 16.7 (14.9-18.7) |
| ≥3 | 0.5 (0.4-0.7) | 10.3 (9.6-11.0) | 0.4 (0.1-1.7) | 4.9 (2.5-9.5) | 0.5 (0.1-0.3) | 2.7 (2.1-3.5) |
| **Males circumcised** | - | 39.4 (38.2-40.6) | - | 23.0 (16.0-31.9) | - | 26.8 (23.4-30.6) |
| **HIV prevalence** †** | 14.2 (13.5-15.0) | 9.7 (9.0-10.4) | 12.5 (11.6-15.9) | 8.4 (6.6-10.4) | 8.3 (7.4-9.9) | 6.1 (5.6-6.6) |
| *Central Region 1 is the geographic sub-region within the Ugandan DHS that contains the Rakai District. Central region 1 also includes Masaka District to the north of Rakai. **Information obtained from 2011 DHS AIDS Indicator Survey Report † Approximately 19-22% of total HIV infected persons in DHS were taking ART (18% in Central Region 1) compared to 22.0% in Rakai. | | | | | | |
